# Development of an antisense oligonucleotide targeting EFEMP1 in models relevant to macular degeneration

**DOI:** 10.1016/j.omtn.2026.103024

**Published:** 2026-07-21

**Authors:** William P. Miller, Said Arevalo-Alquichire, Paula Perez-Corredor, Rose Lin, Anil Upreti, Jeysson Sanchez-Suarez, Tim E. Vanderleest, Claudia Marino, Donovan Spencer, Matthew Scribner, Jebgy Vargas, Andres Muriel-Torres, Xinyao Hu, Yvonne Adu-Rutledge, Bryan A. Kaplan, Karim W. Barake, Audrey L. Gunawan, Aruvi Vijikumar, Reeya Shrestha, Anton Lennikov, Leo A. Kim, Joseph F. Arboleda-Velasquez, Andrew Peterson, Sudhir Agrawal, Vinod Vathipadiekal, Carlos Loya, Michael O’Hare

**Affiliations:** 1 Schepens Eye Research Institute of Mass Eye and Ear and the Department of Ophthalmology at Harvard Medical School, Boston, MA 02114, USA; 2 Department of Neurology, Sealy Institute for Drug Discovery and Mitchell Center for Neurodegenerative Diseases, The University of Texas Medical Branch, Galveston, TX, USA; 3 Mass Eye and Ear and the Department of Ophthalmology at Harvard Medical School, Boston, MA 02114, USA; 4 Aldebaran Therapeutics, Waltham, MA, USA; 5 Alloy Therapeutics, Waltham, MA, USA; 6 Wellcome-Wolfson Institute for Experimental Medicine, Queen’s University Belfast, Belfast, UK

**Keywords:** MT: oligonucleotides, therapies and applications, EFEMP1, antisense oligonucleotides, autsomal dominant drusen, age-related macular degeneration, angiogenesis

## Abstract

Antisense oligonucleotides (ASOs) are RNA-targeting therapeutics with broad potential for genetically defined and complex ocular diseases. The eye is particularly well suited for ASO delivery because of its compartmentalized anatomy, accessibility, and capacity for sustained intraocular drug retention. Autosomal dominant drusen (ADD), an inherited retinal dystrophy characterized by early drusen formation and secondary choroidal neovascularization (CNV), is caused by the EFEMP1 R345W mutation. Mutant EFEMP1 accumulates within the retinal pigment epithelium (RPE) and extracellular matrix, contributing to disease pathology. EFEMP1 is also elevated in the serum and RPE/choroid of patients with age-related macular degeneration (AMD), although its functional role in AMD remains incompletely defined. Here, we developed a biallelic EFEMP1-targeted ASO that achieved potent knockdown and modulated disease-relevant pathways in iPSC-derived RPE, human microvascular retinal endothelial cells (HMRECs), *ex vivo* human choroidal explants, and *in vivo* mouse models. EFEMP1 protein levels were increased in AMD tissue, and ASO-mediated EFEMP1 knockdown reduced complement factor 3 (C3), suppressed HMREC proliferation and migration, and inhibited angiogenic sprouting in choroidal explants. These findings support EFEMP1 as a pathogenic regulator of retinal degeneration and vascular dysfunction and establish EFEMP1-directed ASOs as a promising therapeutic strategy for ADD and AMD.

## Graphical abstract

**Figure:**
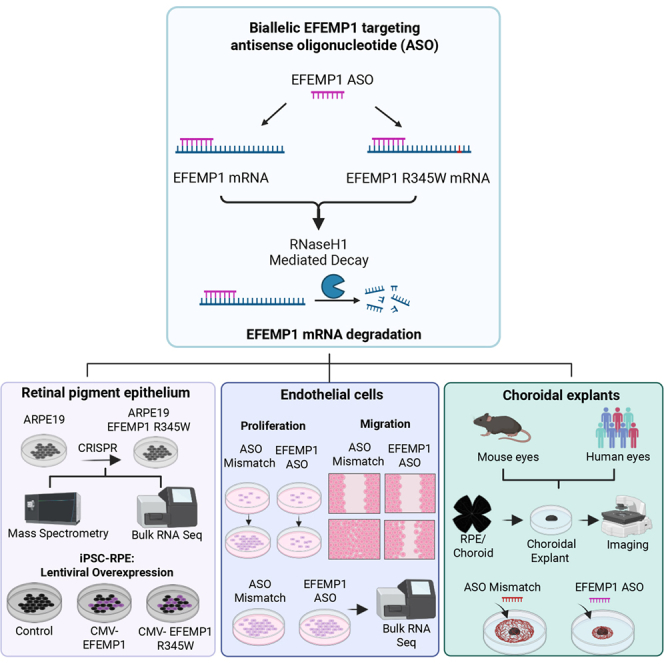

## Teaser

O’Hare and colleagues developed antisense oligonucleotides targeting EFEMP1 and demonstrated potent knockdown *in vitro*, *in vivo*, and *ex vivo* using choroidal explants from mouse and human eyes. EFEMP1 suppression modulated complement signaling, inhibited pathological angiogenesis, and identified EFEMP1 as therapeutic target for both autosomal dominant drusen and age-related macular degeneration.

## Introduction

EFEMP1 (also known as Fibulin-3) is an extracellular matrix (ECM) protein expressed in the retinal pigment epithelium (RPE) and choroid that has been implicated in ECM remodeling.^1^^,^^2^^,^^3^^,^^4^^,^^5^ EFEMP1 accumulation is a defining feature of autosomal dominant drusen (ADD), also known as Doyne honeycomb retinal dystrophy (DHRD) or Malattia Leventinese (ML), an inherited macular dystrophy characterized by the formation of numerous drusen.^6^ ADD causes progressive, irreversible loss of central visual acuity due to geographic atrophy and choroidal neovascularization (CNV).^7^ The disease is genetically defined by a non-conservative missense mutation, R345W, in EFEMP1. Efemp1 knockout has been reported to protect against the development of sub-RPE deposits without producing an associated retinal phenotype.^8^

Several EFEMP1 binding partners have been implicated in ocular disease, including complement factor H (CFH), a potent regulator of complement factor 3 (C3) activity.^2^^,^^9^ The proteolytic complement cascade can be activated through the classical, alternative, or lectin pathways, all of which converge at C3.^10^ Cleavage of C3 generates C3b, which is deposited on cell surfaces and ECM; C3 is also a constituent of drusen and contributes to drusen formation. Another EFEMP1 binding partner is TIMP3, a potent anti-angiogenic factor that competes with vascular endothelial growth factor (VEGF) for VEGFR2.^11^ Interestingly, mutations in TIMP3 cause Sorsby fundus dystrophy (SFD), which shares drusen formation with ADD but is characterized by a more severe CNV response.^12^

Although ADD often progresses more rapidly than age-related macular degeneration (AMD), the two diseases share several clinical and pathological features.^13^ AMD is a debilitating progressive eye disease, clinically characterized by drusen formation, geographic atrophy, and CNV, that affects more than 19.8 million people in the United States. Geographic atrophy often referred to as late-stage dry AMD, is characterized by progressive neurodegeneration and accumulation of extracellular drusen and basal deposits. These lipid-containing aggregates can impair RPE and photoreceptor health by disrupting nutrient transport, promoting inflammation, and increasing the risk of CNV.^14^^,^^15^^,^^16^^,^^17^^,^^18^ Despite recent FDA approvals of complement pathway inhibitors (Pegcetacoplan; Apellis Pharmaceuticals^19^/Avacincaptad pegol; Astellas Pharma^20^) for geographic atrophy, there still remains an urgent need for additional therapeutic options. In addition, complement inhibition is suspected of increasing the risk of retinal vasculitis.^21^

An emerging therapeutic strategy involves the use of antisense oligonucleotides (ASOs), which are versatile RNA-binding molecules that can effectively suppress mRNA translation, select for splice variants, or increase expression of preferred RNA transcripts.^22^ Over 25 years ago, Fomivirsen (branded as Vitravene; Novartis, Basel Switzerland), a first-generation ASO, was approved for weekly intravitreal injection for cytomegalovirus (CMV) retinitis, and was later withdrawn from market.^23^ However, the therapeutic potential of ASOs for ocular diseases is far from being fully realized. Several major breakthroughs in ASO chemistry have resulted in the development of new generations of gapmer, or, that are resistant to RNAse H-mediated decay increasing their durability.^24^ To date, 13 ASO formulations have been approved by the Food and Drug Administration (FDA) for a variety of indications.^25^

*EFEMP1* has been shown to be one of the top ten genes overexpressed in the RPE/choroid during aging.^26^ Previous studies have demonstrated that EFEMP1 is upregulated in the RPE/choroid in dry AMD and in the serum, RPE, and choroid of patients with wet AMD.^27^ Here, we evaluated the role of EFEMP1 in the development of pathologies associated with ADD and AMD, and assessed EFEMP1 targeting ASOs as a potential therapeutic strategy.

## Results

### CRISPR-Cas9 edited ARPE19 cells express EFEMP1^R345W^

**Figure 1:**
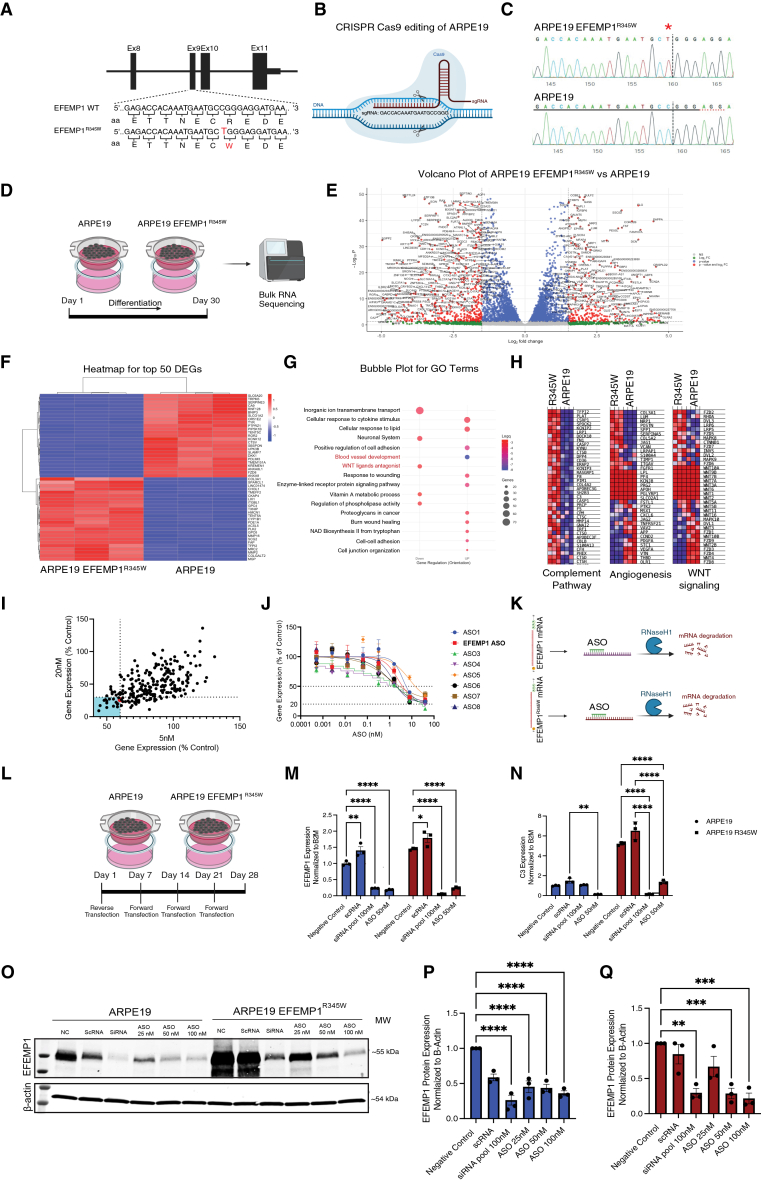
Development of an ARPE19 EFEMP1^R345W^ mutant cell line for screening of EFEMP1 targeting ASOs (A) Schematic of comparison of EFEMP1 and EFEMP1^R345W^ nucleotide sequence and associated amino acid change from arginine to tryptophan. (B) Schematic showing sgRNA-seq for CRISPR-Cas9 editing of ARPE19 cells. (C) Sanger sequencing of ARPE19 and ARPE19 R345W confirming successful nucleotide replacement. (D) Schematic showing ARPE19 and ARPE19 R345W cell differentiation across 30 days prior to bulk RNA sequencing. (E and F) Volcano plot showing significant gene changes in ARPE19 R345W vs. ARPE19 cells (*N* = 3 per group). (F) Heatmap of the top 50 DEGs from ARPE19 R345W and ARPE19 cells. (G) Bubble plot for Gene Ontology (GO) terms enriched in ARPE19 R345W cells. (H) Heatmap for complement, angiogenesis, and WNT signaling pathways identified by GSEA. (I) Graph showing two-dose screen (5 and 20 nM, in triplicate) of 247 ASOs directed at hEFEMP1 compared to untreated cell control. (J) Graphs showing dose-response (0.5 pM–40 nM) of top candidate ASOs. (K) Schematic of biallelic antisense oligonucleotides that target the EFEMP1 and EFEMP1^R345W^. (L) Schematic of transfection protocol of small interfering RNA (siRNA) pool and EFEMP1 targeted ASO in ARPE19 and ARPE19 R345W cells. (M and N) qPCR data of EFEMP1 mRNA expression after treatment with siRNA pool (100 nM), scRNA (100 nM), ASO (50 nM) in ARPE19 or ARPE19 R345W cells (*N* = 3 per group). (N) qPCR data of C3 mRNA expression after treatment with siRNA pool (100 nM), scRNA (100 nM), ASO (50 nM) in ARPE19 or ARPE19 R345W cells (*N* = 3 per group). (O) Western blotting showing ARPE19 EFEMP1^R345W^ cell line has increased expression of EFEMP1 relative to untreated wild type control. (P and Q) EFEMP1 targeting siRNA pool 100 nM, EFEMP1 directed ASOs 50 and 100 nM, substantially knockdown EFEMP1 (*N* = 3, biological replicates). Data were analyzed using a one-way ANOVA with Tukey’s post hoc test for multiple comparisons ∗*p* < 0.05; ∗∗*p* < 0.01; ∗∗∗*p* < 0.001; ∗∗∗∗*p* < 0.0001.

EFEMP1^R345W^ expressing ARPE19 cells were generated using CRISPR-Cas9 editing of exon 9 (Figure 1A). A single guide RNA sequence (sgRNA-seq) was used to direct CRISPR-Cas9 editing (Figure 1B), and the expected sequence alteration was confirmed by sequencing (Figure 1C). We then differentiated edited ARPE19 EFEMP1^R345W^ for 30 days to compare the transcriptomic profile with ARPE19 cells (Figure 1D). We carried out bulk RNA sequencing (RNA-seq) on ARPE19 and ARPE19 EFEMP1^R345W^ (or ARPE19 R345W) cells and show differentially expressed genes (DEGs) in a volcano plot (Figure 1E; bulk RNA-seq data are available in GEO:GSE289976). A heatmap of the top 50 DEGs including (upregulation of genes associated with ECM remodeling including *COL3A1*, *MMP2*, *MMP16*, and *COLGALT2*) (Figure 1F). Gene Ontology (GO) analysis for downregulated genes in ARPE19 R345W reveals enrichment in inorganic ion transmembrane transport, neuronal system, WNT ligand antagonists, vitamin A metabolic process, and regulation of phospholipase activity (full list of GO terms available in Table S1) while the upregulated genes associated with cellular response to cytokine stimulus, cellular response to lipid, positive regulation of cell adhesion, blood vessel development, response to wounding, enzyme-linked receptor protein signaling pathway, proteoglycans in cancer, burn wound healing, NAD biosynthesis II from tryptophan, cell-cell adhesion, and cell junction organization (Figure 1G). Gene set enrichment analysis (GSEA) identified complement pathway, angiogenesis and WNT signaling as some of the terms found by gene enrichment analysis. (Figure 1H). These data are consistent with the reported complement phenotype previously observed in ARPE19 R345W cells, indicating that we have developed a reliable model to screen drug candidates to reduce EFEMP1 expression. To identify a potent EFEMP1-targeted ASO, we first screened 247 drug-like candidates and identified EFEMP1-directed ASO candidates that efficiently reduce the mRNA expression of EFEMP1 *in vitro* by more than 50% relative to control (Figure 1I). The top 8 candidates were evaluated in an eight-concentration dose-response assay, leading to the selection of a lead candidate (EFEMP1 ASO; IC50 of 3.807 nM) that was taken forward for further investigations (Figure 1J). We then used the ARPE19 and ARPE19 R345W cells to assess ASO-mediated reduction of both EFEMP1 and EFEMP1^R345W^ expression (Figure 1K). We aimed to identify an ASO capable of reducing either EFEMP1 or EFEMP1 R345W expression *in vitro* by reverse transfection on day 1 and forward transfection up to 21 days (Figure 1L). We determined 50 nM of EFEMP1 ASO significantly reduced EFEMP1 and EFEMP1^R345W^ mRNA expression levels in ARPE19 and ARPE19 R345W cells, respectively (Figure 1M). While ARPE19 R345W cells demonstrated significantly (5×) increased levels of complement factor 3 (C3) compared to ARPE19 cells. We report that ASO knockdown of EFEMP1 significantly reduced C3 expression in both ARPE19 and ARPE19 R345W cells (Figure 1N). We further confirmed the reduction of EFEMP1 at the protein level after treatment with either scramble (sc)RNA (100 nM), a short interference (si)RNA pool (100 nM) that is comprised of four independent EFEMP1 targeting sequences or different concentrations of an EFEMP1 ASO (25, 50, and 100 nM) in ARPE19 or ARPE19 R345W cells (Figure 1O). The quantitative analysis by densitometry demonstrated significant reduction in EFEMP1 protein expression with EFEMP1 ASO 25, 50, and 100 nM (Figure 1P) and EFEMP1 R345W (50 and 100 nM) (Figure 1Q) in the ARPE-19 cultures. To assess ECM changes in ARPE19 R345W cells, we carried out mass spectrometry after decellularization of transwells after a month of culture of ARPE19 and ARPE19 R345W cells (Figure S1A). We report a 14.2-fold increase in EFEMP1 protein levels in ARPE19 R345W ECM (Figure S1B). Ingenuity pathway analysis (IPA) was used to highlight predicted networks of interest and indicates increased protein kinase b (AKT), phosphoinositide 3-kinase (PI3K) pathway activation as well as reduced APP-related pathways (Figures S1C–S1E). High temperature requirement protein A1 (HTRA1) is upregulated (5.95-fold) in the ECM of ARPE19 R345W cells. HTRA1 variants are highly significant genetic risk factors for AMD. The upregulation of HTRA1 in the ECM of ARPE19 R345W cells is striking (Figures S1B, S2A, and S2B). We confirmed protein level increases of HTRA1 using western blotting of whole cell lysates of ARPE19 R345W cells (Figure S2C).

### Lentiviral overexpression of EFEMP1 and EFEMP1^R345W^ is inhibited by EFEMP1-targeted ASOs in iPSC-RPE cells

**Figure 2:**
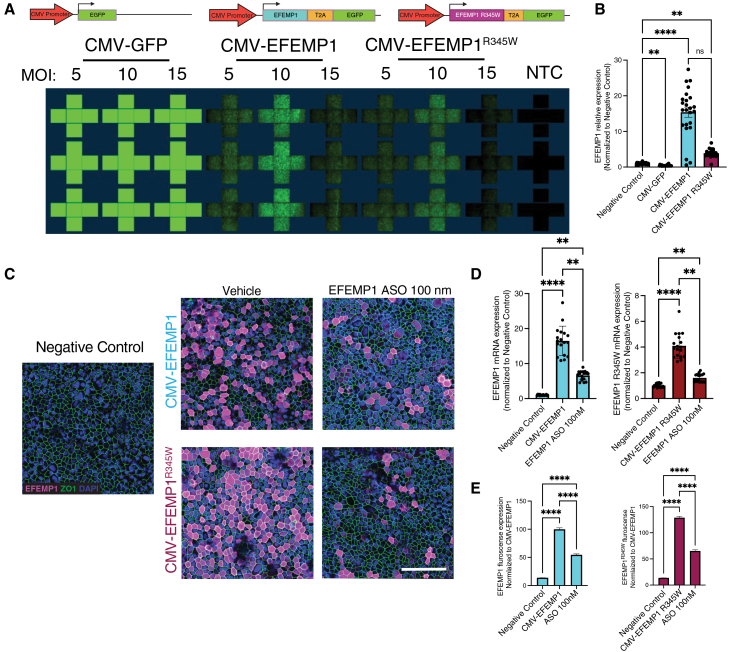
Lentiviral overexpression of EFEMP1 and EFEMP1 R345W in iPSC-RPE can be efficiently knocked down using a EFEMP1-targeted ASO (A) Comparison of lentiviral transduction efficiency of constructs expressing either EGFP, EFEMP1 with a T2A linkage to EGFP or EFEMP1^R345W^ with a T2A linkage. (B and C) qPCR of EFEMP1 expression in iPSC-RPE. (C) Representative confocal images in iPSC-RPE showing Lentiviral overexpression of either wild type EFEMP1 and EFEMP1R345W (*n* = 4, biological replicates). DAPI, blue nuclear stain; ZO-1, tight junction protein marked in green; EFEMP1, visualized in magenta (×40, scale bars, 100 μm). (D) qPCR showing mRNA expression of EFEMP1 levels after EFEMP1 overexpression and effective of EFEMP1 targeted ASOs (*N* = 3, biological replicates). (E) EFEMP1 intensity quantified relative to total cells in field via watershed algorithm and automated fluorescence quantification in iPSC-RPE cells overexpressing EFEMP1 comparing EFEMP1 expression after treatment ASO 100 nM or a siRNA pool 100 nM. Data were analyzed using a one-way ANOVA with Tukey’s post hoc test for multiple comparisons. ∗∗*p* < 0.01; ∗∗∗∗*p* < 0.0001.

We tested a lentiviral CMV promoter-driven construct encoding either enhanced green fluorescent protein (EGFP) alone or EFEMP1 and EFEMP1^R345W^ that were conjugated to EGFP by a T2A linker (Figure 2A). We report robust EGFP expression after transduction of the CMV_EGFP construct at multiplicity of infection (MOI): 5, 10, or 15 in induced pluripotent stem cells (iPSC)-derived RPE cells. We report that CMV_EFEMP1 and CMV_EFEMP1 ^R345W^ (MOI:10) showed the strongest EGFP expression. Therefore, all future experiments were conducted under those parameters. qPCR revealed significant upregulation of EFEMP1 levels after treatment with CMV_EFEMP1 or CMV_EFEMP1^R345W^ in iPSC-RPE cells (Figure 2B**)**. We confirmed that the EFEMP1 ASO (100 nM) could significantly reduce EFEMP1 mRNA levels compared to the vehicle control or an ASO mismatch in iPSC-RPE cells (Figure S3). Immunofluorescence staining and confocal imaging of iPSC-RPE reveals robust overexpression of EFEMP1 and EFEMP1 R345W after lentiviral transduction compared to the negative control cells using an anti-EFEMP1 antibody (magenta) in human iPSC-derived RPE cells (Figure 2C). The successful reduction of EFEMP1- and EFEMP1 R345W-induced overexpression via an EFEMP1-directed ASO (100 nM) was confirmed by qPCR and fluorescence quantification using an automated method (Figures 2D and 2E; Figure S4). We report significant increases in C3 expression after EFEMP1 or EFEMP1 R345W overexpression. The EFEMP1 ASO significantly reduced C3 levels in both groups (Figures S5A and S5B).

### EFEMP1 knockdown inhibits angiogenic properties of HMRECs

**Figure 3:**
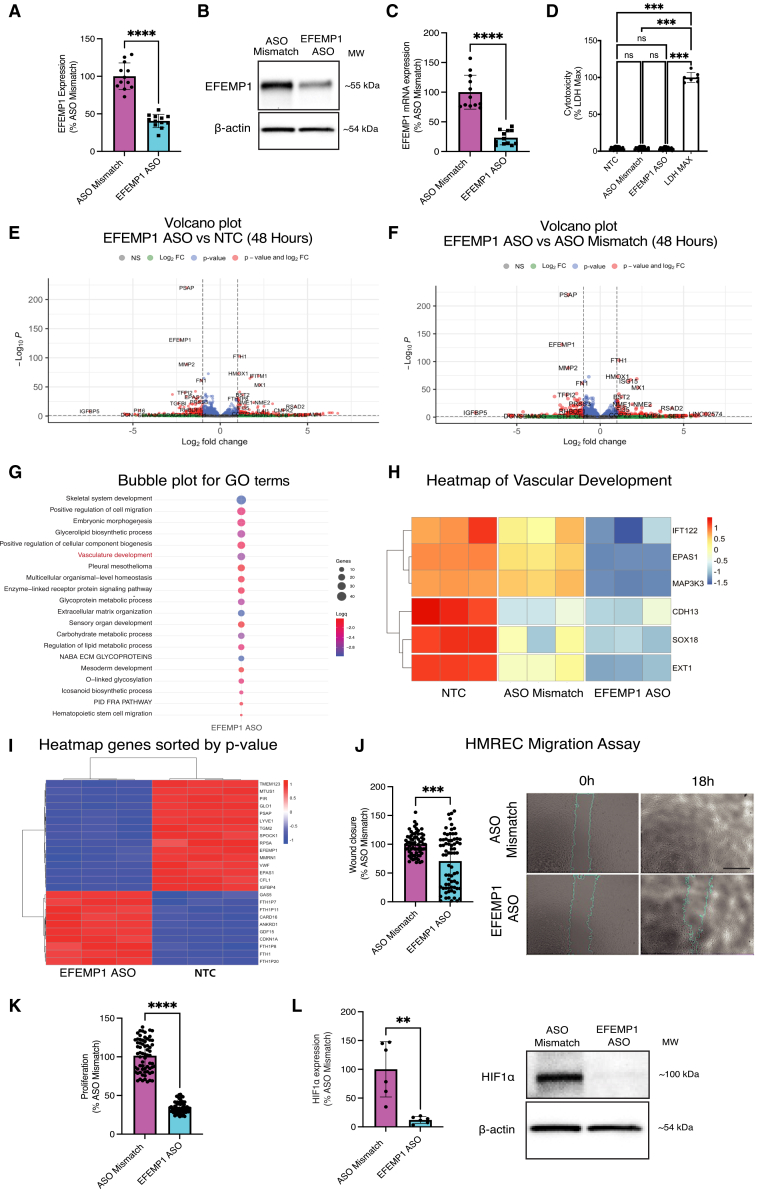
EFEMP1-directed ASOs reduced endothelial proliferation, migration and pathways relevant to vascular development (A and B) Western blotting of ASO mismatch (50 nM) vs. EFEMP1 ASO (50 nM) in human microvascular endothelial cells (HMRECs) (*N* = 3, biological replicates). (C and D) qPCR of EFEMP1 knockdown comparing ASO mismatch (50 nM) vs. EFEMP1 ASO (50 nM) in HMRECs. (D) LDH assay comparing ASO mismatch and EFEMP1 ASO (50 nM) compared to LDH max (one-way ANOVA; *p* < 0.001). (E) Volcano plot of bulk RNA-seq comparing EFEMP1 ASO and NTC in HMRECs (*n* = 3 per group). (F) Volcano plot of bulk RNA-seq comparing EFEMP1 ASO and ASO mismatch (*n* = 3 per group). (G) Bubble plot for Gene Ontology (GO) terms enriched in EFEMP1 ASO endothelial cells. (H) *Z* score heatmap displays vascular development genes from EFEMP1 ASO, ASO mismatch and NTC in HMRECs. (I) Heatmap of the top DEGs from EFEMP1 ASO and NTC in HMRECs. (J and K) Bar graph of migration assay comparing ASO mismatch and EFEMP1 ASO (50 nM) and representative bright field images at 0 and 18 h (*N* = 3 biological replicates, *n* = 50, technical replicates, scale bars, 750 μm). (K) cyQUANT proliferation assay shows significant reduction in proliferation after treatment with EFEMP1 ASO (50 nM). (L) Bar graph of HIF1α protein expression and representative western blot (*n* = 6). Data were analyzed by Mann-Whitney *U* test or Kruskal-Wallis with Dunn’s post hoc test for multiple comparisons. ∗∗*p* < 0.01; ∗∗∗*p* < 0.001; ∗∗∗∗*p* < 0.0001.

The transcriptomics analyses performed on RPE cell cultures identified genes from several pathways relevant to angiogenesis that were differentially regulated in response to the presence of the EFEMP1 R345W mutation (see Figure 1H**)**. Thus, as a subsequent investigation within our study, we evaluated the impact of EFEMP1 knockdown on endothelial cells to determine whether targeting EFEMP1 held potential antiangiogenic effects. To assess this, primary cultures of human microvascular retinal endothelial cells (HMRECS) were transfected with either our EFEMP1 ASO (EFEMP1 ASO), or a version of the EFEMP1 ASO that contains nucleotide substitutions in the core sequence to disrupt interaction with the target EFEMP1 sequence (ASO mismatch, Figure S3), or negative control. Relative to the ASO mismatch, 50 nM of EFEMP1 ASO was sufficient to significantly reduce EFEMP1 expression in HMRECs at the protein level by ∼60%, and at the mRNA level by ∼80% with no cytotoxicity as measured by LDH secretion. (Figures 3A–3D). After determining that the EFEMP1 ASO could be applied to HMRECs in culture, bulk transcriptomics analysis was performed on RNA isolated from non-treated cells (NTCs), cells transfected with ASO mismatch and cells transfected with EFEMP1 ASO 48 h after treatment (data available in GEO: GSE289976). Analysis of the transcriptomics data revealed several differentially regulated genes between the EFEMP1 ASO, and the control groups. Several prominent genes are downregulated after treatment with EFEMP1 ASO including PSAP, EFEMP1, MMP2, and CFH, while gene including HMOX1, FTH1, and CDKN1A were upregulated (Figures 3E and 3F). Further analysis to establish GO terms identified multiple pathways relevant to angiogenesis and other related pathologies (Figure 3G; Table S2). GSEA identified vasculature development as one of the enriched terms. We took the genes listed in the GSEA term vasculature development and display the identified DEGs in a heatmap (Figure 3H**)**. To complement our GO analysis, we performed GSEA to identify other relevant factors associated with the bulk RNA-seq data. This led to the identification of the gene EPAS1, commonly known as hypoxia-inducible factor 2α (HIF2α), which is more selectively expressed in endothelial cells relative to the ubiquitously expressed HIF1α variant. HIF2α, which is associated with vascular development, was significantly downregulated after treatment with the EFEMP1 ASO. Other relevant genes included IFT122, MAP3K3, CDH13, SOX18, CDKN1A, and EXT1 (Figure 3I). We then preformed functional studies to determine how EFEMP1 knockdown was influencing endothelial cell behavior. A scratch wound assay was performed 48 h post-transfection to assess the impact of the EFEMP1 ASO on endothelial cell migration. After 18 h, HMRECS transfected with the EFEMP1 ASO exhibited a significant reduction in migratory capacity relative to cells transfected with the ASO mismatch (Figure 3J). As an additional measure of angiogenic potential, EFEMP1 knockdown was concomitant with a significant decrease in cellular proliferation 24, 48, and 72 h after transfection (Figure 3K; Figure S6). HIF1α expression was evaluated 48 h post-transfection by western blotting and relative to the ASO mismatch, a significant reduction in HIF1α expression was observed at the protein level of ∼80% (Figure 3L**)**. We utilized *in silico* sequence-complementarity analysis using the EFEMP1 ASO sequence. Candidate off-target genes were explored using three complementary approaches: full 20-mer mismatch scanning, central gapmer-core matching, and DNA:RNA duplex free-energy ranking. EFEMP1 was excluded from off-target enrichment statistics because it represents the intended on-target transcript. The highest-priority off target candidates were RHBDF1 and VAC14, both of which contained two mismatches to the ASO target sequence, showed predicted DNA:RNA duplex stability, and were downregulated in the RNA-seq dataset. These computational predictions identify candidate sequence-dependent off-targets but do not prove direct RNase-H-mediated cleavage (Figures S7A and S7B).

### EFEMP1 knockdown inhibits growth in *ex vivo* choroidal explants derived from mouse and human tissue

To enhance the translational value of our findings, we first performed intravitreal injections of the EFEMP1 ASO in age-matched, 12-week-old C57BL/6J mice at doses including 1, 10, and 50 μg to validate the EFEMP1 knockdown *in vivo*. After 72 h, the eyes were enucleated, separating the anterior and posterior segments (Figure S8A). Posterior segments were then homogenized, and EFEMP1 mRNA levels were quantified at each of the doses tested. While the 1 μg dose showed no reduction in EFEMP1 mRNA expression relative to the vehicle control, significant decreases in EFEMP1 levels were obtained at the 10 and 50 μg doses. We then evaluated the duration of the knockdown effect. Mice injected with 15 μg of EFEMP1 ASO maintained significant EFEMP1 knockdown up to 30 days post-injection relative to vehicle controls (Figure S8B). We have shown strong EFEMP1 expression in retinal muller cells from adult C57BL/6 mice (Figure S9A). Retinal structure is preserved with no reactive gliosis (glial fibrillary acidic protein [GFAP] staining) in EFEMP1 knockout animals. GFAP expression is generally restricted to retinal astrocytes and nearly absent in Müller cells. Following retinal damage, disease, or stress, Müller cells become rapidly activated (reactive gliosis) and dramatically upregulate GFAP expression. These data suggest that EFEMP1 knockout is unlikely to have a drastic effect on muller cell health and overall retina structure (Figure S9B).

**Figure 4:**
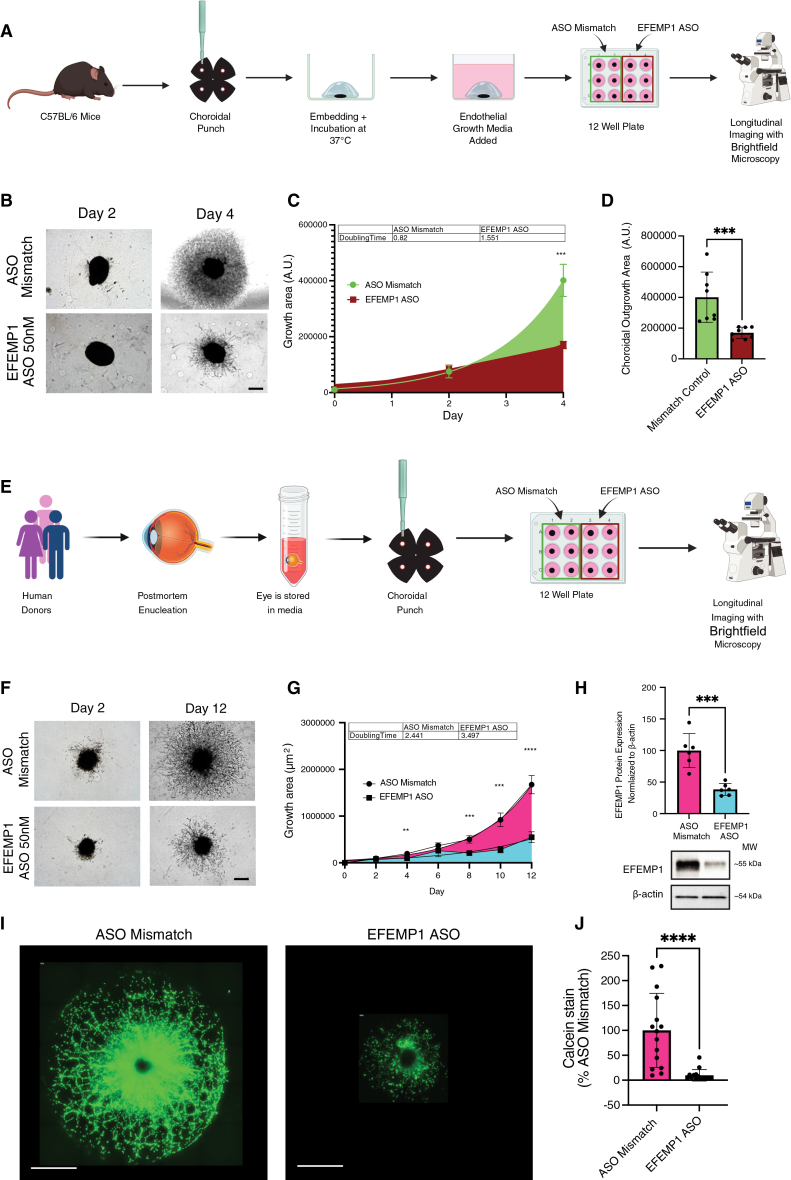
EFEMP1 knockdown reduces choroidal sprouting in both mouse and human tissue (A) Schematic of experimental design for mouse choroidal explant isolation, embedding, treatment and imaging. (B) Bright field images of mouse choroidal explants at day 2 and day 4 after transfection with ASO mismatch control or EFEMP1-targeted ASO (50 nM). (C) Chart showing the growth rate of mouse choroidal explants across time (*N* = 3, biological replicates, *n* = 8, technical replicates). (D–F) Bar graph of mouse choroidal outgrowth area treated with ASO mismatch or EFEMP1 ASO (50 nM; *n* = 8). (E) Schematic of human choroidal explant isolation, embedding, treatment and imaging. (F) Bright field images of human choroidal explants at day 2 and day 12 after transfection with ASO mismatch control or EFEMP1-targeted ASO (50 nM). (G and H) Chart showing the growth rate of human choroidal explants across time (*N* = 3, biological replicates, *n* = 12, technical replicates) (H) Representative western blot of EFEMP1 expression compared to β-actin. (I) Representative immunofluorescence image of calcein imaging in human choroidal explants (images were rescaled and framed on a black background). (J) Bar graph of calcein staining quantification comparing ASO mismatch control or EFEMP1 targeted ASO (50 nM) (*N* = 3, biological replicates, *n* = 12, technical replicates). Data were analyzed by multiple paired *t* tests or by Mann-Whitney *U* test. ∗∗∗*p* < 0.001; ∗∗∗∗*p* < 0.0001.

To test the antiangiogenic potential of the EFEMP1 ASO in mouse eyes, we next used mouse choroidal explants as a controlled *ex vivo* system to determine whether EFEMP1 knockdown suppresses angiogenic outgrowth in intact ocular tissue.^28^ In this assay, the RPE/choroid complex is removed from the eye and cultured outside the animal while preserving local interactions among resident choroidal endothelial cells, stromal cells, ECM, and neighboring ocular tissue components that are not captured in monoculture. Sprouting from the edge of the explant therefore provides a tissue-level readout of angiogenic outgrowth in a reproducible experimental system. Mouse eyes were enucleated, the RPE/choroid was isolated, and 1 mm punches were generated from the tissue before embedding in a matrix containing basement membrane extract (Figure 4A). Choroidal explants were transfected with 50 nM of ASO mismatch or EFEMP1 ASO and bright field images were taken every other day over 4 days. The quantification of the total growth area indicated a significant reduction in the growth of EFEMP1 ASO-transfected choroids relative to controls (Figures 4B and 4C**)**. Calcein imaging was also performed on the final day to allow for growth quantification in 3 dimensions while also demonstrating sprout viability (Figure 4D**)**. These data indicated that sprouts consisted of live-cells and confirmed the inhibition in growth caused by the EFEMP1 assay. We then extended this approach to postmortem human choroidal explants as a complementary translational model. In this system, RPE/choroid tissue is isolated from donor eyes after death, embedded in basement membrane extract, and cultured to allow resident human choroidal cells to migrate and sprout into the surrounding matrix. Although the tissue is no longer connected to systemic circulation, this model preserves human ECM, resident choroidal cell populations, and donor-specific tissue features, while still allowing controlled *ex vivo* treatment with ASO mismatch or EFEMP1 ASO. (Figure 4E**)**. Under the same conditions, explant outgrowth transfected with EFEMP1 ASO 50 nM was also significantly reduced in human tissue as determined by quantification using brightfield microscopy (Figures 4F and 4G**)**. Successful transfection and knockdown of EFEMP1 in the human choroidal explants was also confirmed by western blotting (Figure 4H**)**. Calcein imaging further substantiated a significant reduction in outgrowth of EFEMP1 ASO-transfected choroids relative to ASO mismatch controls (Figure 4I**)**.

### EFEMP1 is upregulated in AMD and EFEMP1 knockdown reduces AMD choroidal explant growth

**Figure 5:**
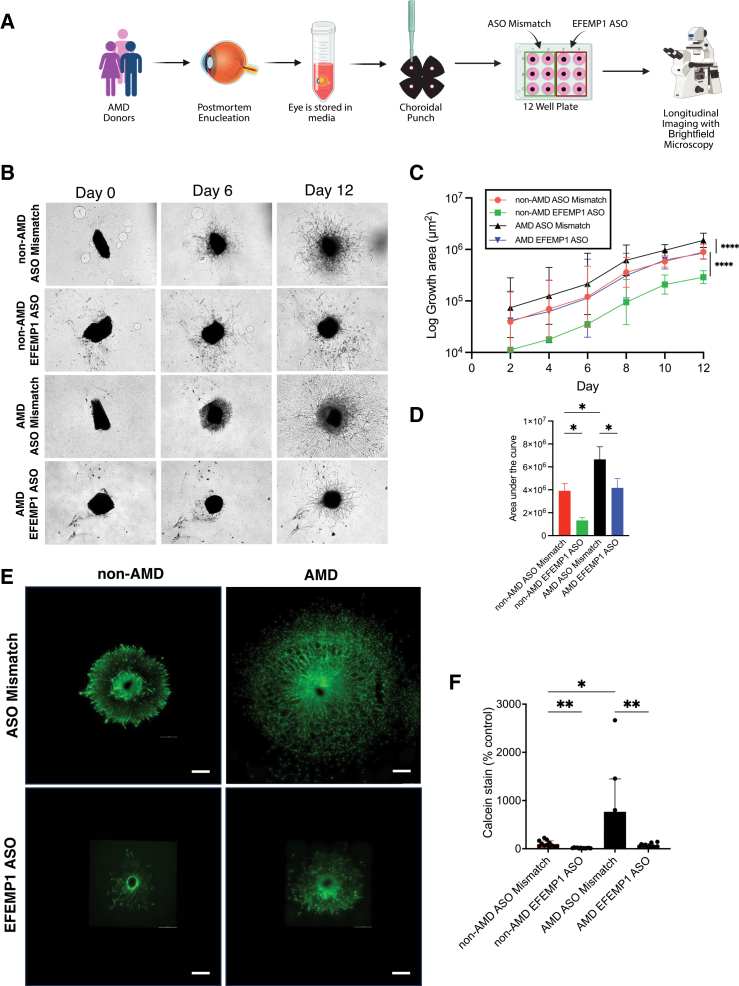
EFEMP1 ASOs reduce AMD choroidal explant growth rates (A) Schematic of experimental design for AMD donor choroidal explant isolation, embedding, treatment and imaging. (B) Bright field images comparing non-AMD and AMD choroidal explants at 0, 6, and 12 days between ASO mismatch and EFEMP1 ASO (50 nM), scale bars, 200 μm. (C and D) Growth rates of non-AMD and AMD choroidal explants with ASO mismatch and EFEMP1 ASO (50 nM). (D) Bar graph comparing area under the curve from non-AMD and AMD choroidal explants with ASO mismatch and EFEMP1 ASO (50 nM). (E) Calcein immunofluroscence comparing ASO mismatch and EFEMP1 ASO (50 nM), (images were rescaled and framed on a black background), (scale bars, 1 mm). (F) Bar graph comparing calcein staining between ASO mismatch and EFEMP1 ASO (50 nM), (*N* = 3 biological; *n* = 12 technical). Data were analyzed by multiple paired *t* tests or by Mann-Whitney *U* test. ∗∗*p* < 0.01; ∗∗∗*p* < 0.001; ∗∗∗∗*p* < 0.0001.

To assess the levels of EFEMP1 and its binding partner TIMP3 in AMD, we dissected the RPE/choroid from enucleated eyes obtained postmortem from patient donors and compared expression in AMD and non-AMD derived tissue (for demographics, see Table S3). This provided a disease-relevant extension of the human explant model and enabled assessment of whether EFEMP1 knockdown suppresses outgrowth in both non-AMD and AMD RPE/choroid tissue. Immunofluorescence staining revealed clear increases in EFEMP1 protein staining (green) in the RPE of the AMD sample compared to the non-AMD (healthy control) eye. TIMP3 (magenta) staining is in close proximity to staining for EFEMP1 and is associated with cell junctions in the AMD eyes (Figure S10A). Western blotting of the RPE/choroid confirms the upregulation of EFEMP1 in AMD (Figure S10B). Bright field images show reduced growth of non-AMD and AMD choroidal explants at 0, 6, and 12 days after EFEMP1 ASO treatment (Figures 5A and 5B). We determined that treatment with 50 nM EFEMP1 ASO significantly reduced the growth rates of AMD choroidal explants compared to AMD explants treated with ASO mismatch (Figure 5C**)**. We compared area under the curve (Figure 5D**)** We assessed non-AMD and AMD choroidal explants using Calcein imaging and confirmed a significant reduction in outgrowth after EFEMP1 ASO treatment relative to ASO mismatch controls (Figures 5E and 5F**)**.

## Discussion

The potential of EFEMP1-targeted ASOs therapy in the treatment of ADD and AMD represents an exciting and novel paradigm against these debilitating ocular conditions. ADD and wet AMD share abnormal CNV as a common pathological phenotype and subsequent retinal damage, which is primarily treated with anti-VEGF therapies, which aim to inhibit pathological blood vessel growth.^27^ However, while anti-VEGF agents are effective, they often require frequent injections and may not fully address the underlying molecular mechanisms driving disease progression.^29^ This has led to increasing interest in exploring alternative therapeutic strategies that target other key players in the pathogenesis of ADD and wet AMD, such as EFEMP1.

Furthermore, as a secreted ECM protein, EFEMP1 plays a critical role in the regulation of ECM remodeling, complement factor expression, and angiogenesis.^27^ These processes are impacted during both ADD and AMD progression. We propose that targeting EFEMP1 may have several advantages over the current FDA-approved therapies such as pegcetacoplan and avacincapted pegol that target either C3 or C5 alone.^30^^,^^31^ Previous literature reports indicated that EFEMP1 modulation impacts several complement factors implicated in AMD pathophysiology, including C3, CFH, and CFB.^2^^,^^32^ We found highly significant upregulation of C3 in EFEMP1^R345W^ cells and downregulation of C3 in response to EFEMP1 knockdown. Furthermore, EFEMP1-targeted ASOs reduced CFH expression in HMRECs after 48 h. C3 expression, activation, and subsequent complement pathway activation are strongly associated with drusen formation and disease progression in AMD.^33^ C3-convertase (C3bBb) is formed through complement factor B and factor D activity. This drives an amplification loop by cleaving more C3 into C3b. Importantly, this cascade can be halted by CFH-mediated recruitment of complement factor I, which cleaves C3b and inactivates it. EFEMP1 R345W mice develop sub-RPE deposits reminiscent of drusen after and introducing C3 or CFB knockout alleles into these mice prevents sub-RPE deposit formation,^3^ indicating a pathological link between EFEMP1, complement activity, sub-RPE deposition development, and disease progression.

Interestingly, high-temperature requirement protein A1 (HTRA1) accumulates in the ECM of ARPE19 R345W cells by over 20-fold compared to ARPE19 wild-type (WT) cells. Variants of HTRA1 are associated with AMD risk, and alternations in HTRA1 function have been reported in other macular dystrophy’s including late onset retinal degeneration (L-ORD).^34^ HTRA1 was recently found to cleave EFEMP1; however, the implications of cleavage have not been explored. It was further reported that HTRA1 cleaves both EFEMP1 and EFEMP1 R345W with similar efficiency in cell-free systems; however, in the context of ADD EFEMP1 R345W accumulations are robust and may require greater levels of serine protease activity to break down EFEMP1 that has accumulated beneath the RPE.^35^

EFEMP1 modulation also impacts other factors expression including ECM remodeling factors such as MMP2 and COL3A1 that have recently received attention in the context of drusen formation for Sorsby’s fundus dystrophy and AMD.^36^ We show clear evidence that MMP2 is upregulated in ARPE19 R345W cells consistent with previous reports^2^; however, we now provide evidence that EFEMP1 knockdown reduces MMP2 expression significantly in HMRECs. We propose that EFEMP1 regulates MMP2 expression through altered Notch signaling levels as EFEMP1 contains a Delta, Serrate, Lagg (DSL) domain and Notch signaling is a known downstream effector of EFEMP1.^37^ Supporting the relationship between Notch signaling and EFEMP1, GSEA revealed Notch signaling as an enriched pathway in ARPE19 R345W with an upregulation of Notch2, DLL1, and JAG1. Furthermore, HMRECs treated with the EFEMP1 ASO had altered Notch signaling with a downregulation in genes, including JAG1 and HES1.

In the context of angiogenesis, EFEMP1 knockdown drastically reduces endothelial cell proliferation and migration in HMRECs and choroidal explants from both mouse and human eyes. GO analysis highlighted vascular development to be downregulated after EFEMP1 ASO treatment at 48 h. We found that a major gene downregulated within the vascular development pathways is EPAS1 (HIF2α), which has previously been found to be upregulated in correlation with EFEMP1 upregulation. Further, EFEMP1 was suggested to be a downstream effector of HIF2α.^38^ Our data suggest that EFEMP1 downregulation may be directly affecting HIF2α expression or alternatively via a feedback loop mechanism. Alterations in other genes associated with hypoxia after EFEMP1 ASO treatment was found by GSEA. Given the robust reduction in proliferation and migration, we also assessed the levels of HIF1α, a variant that is more ubiquitously expressed than HIF2α. We found that indeed HIF1α protein levels were significantly reduced at 48 h. Given that HIF1α levels were not altered at the transcriptomic level, we postulate that during normal oxygen levels, excess prolyl hydroxylase (PHD) enzymes are free to bind HIF1α due to the dramatic reduction in EPAS1 expression. PHD enzymes typically catalyze the hydroxylation of two conserved proline residues in both HIF-2α and HIF-1α.^39^ Similarly, increased hydroxylation of HIF-1α in the absence of strong expression of HIF2α by Von Hippel-Lindau (pVHL) protein could be predicted. Typically, the binding of HIF-α proteins to pVHL results in polyubiquitination and degradation by the 26S proteasome.^40^ Reductions in the levels of HIF1α and HIF2α have been widely reported to have potent anti-proliferative effects.^41^^,^^42^^,^^43^ These data indicate that the anti-angiogenic effects of EFEMP1 knockdown may be beneficial in a range of ocular pathologies associated with aberrant angiogenesis including retinopathy of prematurity and diabetic retinopathy in addition to neovascular AMD.

EFEMP1 has been shown to be upregulated in the retinal and choroidal tissues of AMD patients.^27^ Herein, we confirmed this upregulation of EFEMP1 in human RPE/choroid from donors with AMD, and report EFEMP1 ASOs can significantly reduce choroidal explant growth capacity from human eyes with or without either dry or wet AMD. The choroidal explant sprouting assay has been used with mouse choroidal tissue and found to be a robust and reproducible system. In this study, we adapted the methodology for human choroidal explants, which display a longer duration than the mouse system and exhibit more consistent results. This enabled testing of EFEMP1 ASO in both mouse and human tissue, increasing its translational value.

ASOs are short, synthetic nucleic acid sequences designed to bind to specific RNA targets and modulate gene expression.^44^ By binding to the mRNA of EFEMP1, ASOs can reduce EFEMP1 expression through RNAseH1 recruiting promoting EFEMP1 mRNA degradation, potentially reducing the pathological effects associated with its overexpression. This approach offers several advantages over traditional therapies. Unlike anti-VEGF agents, which target downstream effectors of angiogenesis,^29^ EFEMP1-targeted ASOs address a key upstream factor involved in the regulation of angiogenic signaling pathways, potentially providing an opportunity for earlier intervention and a means of controlling CNV development. Furthermore, ASOs are able to offer a sustained therapeutic effect, reducing the need for frequent injections and improving patient compliance.^45^

Several challenges and considerations exist when evaluating the clinical use of EFEMP1-targeted ASOs in ADD and AMD. One significant issue is the delivery and stability of the ASOs within the retinal tissues. We have shown significant knockdown in the posterior segment of the eye; however, future studies should explore the tissue distribution of the ASO. Furthermore, cationic polymers or lipid nanoparticles could be assessed as potential carriers for ASO studies in the eye, to improve delivery of ASOs to retinal cells at therapeutic concentrations. Additionally, the safety profile of EFEMP1-targeted ASOs must be thoroughly assessed. While ASOs generally have a favorable safety profile compared to small molecule drugs, long-term effects, and potential off-target interactions, there have been reports of ASO-associated lens events in several clinical trials.^46^ These effects may be chemistry or sequence-dependent, but careful attention will need to be given to any off-target effects *in vivo*.

An alternative approach could focus on an allele-specific knockdown of EFEMP1 R345W. However, for single missense variants that differ by a single nucleotide, this can be challenging as the mutation location may not be optimal for ASO binding activity and reduces the number of possible candidate sequences. It is also unclear if the knockdown of a single allele would be sufficient to reduce pathology during clinically relevant ADD once the drusen has begun to form. Given the high levels of EFEMP1, both intracellularly and extracellularly, it could be informative in future studies to understand the relative abundance of EFEMP1 and EFEMP1 R345W. In support of a biallelic approach, patients with biallelic variants in EFEMP1 that result in a near-complete loss of gene expression have been reported to have normal retinal structure, suggesting retinal knockdown of EFEMP1 may not be associated with pathology.^47^ There have been reports of myopia; however, the causality has not been determined to date, this may be a developmental abnormality that may not occur in adult knockdown; however, further research will be needed to determine this. Furthermore, EFEMP1 knockout mice have been found to have normal retinal structure and have been found to protective from sub-RPE deposit formation.^8^

Several limitations of this study should be acknowledged, including the use of ARPE19 cells as a model for screening ASOs. ARPE19 cells provide versatility for *in vitro* genetic manipulation and production of a stable cell culture and act in this study as a research tool to screen a library of EFEMP1-targeted ASOs that would be challenging to perform in primary cells. ADD patient-derived iPSC-RPE would be considered the gold standard *in vitro* model and are not commercially available and were not possible to obtain for this study due to their rarity. However, it is unlikely that using these cells would impact our identification of potent EFEMP1-targeted ASOs that we have now validated *in vitro*, in human iPSC-RPE, primary endothelial cells, in mouse tissue *ex vivo*, and *in vivo* and finally in *ex vivo* human choroidal tissue from AMD donors. Long-term iPSC-RPE culture studies exploring the impact of EFEMP1 overexpression would be beneficial to understand its role in oxidative stress pathways. Future studies should now explore the potential of a combination of EFEMP1-targeted ASOs with anti-VEGF therapy, which may lead to prolonged efficacy while reducing frequency of administration. This is based on the notion that EFEMP1 overexpression may underpin several early pathways that modulate the development of new blood vessels. These data have highlighted a potential role of RPE cells in mediating neovascularization in ADD; future studies should investigate the cross talk between mutant RPE cells with endothelial cells.

In conclusion, we have identified and tested a potent EFEMP1-targeted ASO that offers a promising new avenue for treating ADD and potentially AMD. While preclinical data *in vitro*, *ex vivo*, and *in vivo* are encouraging, further research using *in vivo* models of ADD and AMD-related pathology will be required to assess the full potential of EFEMP1-targeted ASOs and determine the safety, efficacy, and optimal *in vivo* dose. If successful, this approach could add to the existing treatment options, potentially improving outcomes for patients with these debilitating diseases.

## Materials and methods

### Cell culture

ARPE19 cells were purchased from ATCC (CRL-2302). RPE media was prepared as previously described.^48^ Briefly, minimum essential medium alpha (MEM α) (Thermo Fisher Scientific; 12571-063) was supplemented with 1:100 N2 Medium Supplement (Gibco; 17502048), 1:100 glutamine (Lonza; BEBP17-605E), 1:100 non-essential amino acid solution (Gibco; 11140050), 20 μg/L hydrocortisone (Millipore Sigma; H6909), 250 mg/L taurine (Millipore Sigma; T0625) and 0.013 μg/L of triiodo-thyronin (Millipore Sigma; T5516) with, or without 5% heat-inactivated fetal bovine serum (FBS) (HyClone; SH30071.03) were indicated. About 100 μg/mL Normocin (InvivoGen; ant-nr-1) was used as an antibiotic and antifungal agent. For bulk RNA-seq, qPCR and western blotting ARPE19 and ARPE19 R345W cells were grown and differentiated in 6-well transwells for 30 days.

iPSC-RPE (Cellular Dynamics International; R1101) were cultured in media composed of MEM α, 5% KnockOut SR∗ (Thermo Fisher Scientific; 10828-028), 55 nM Hydrocortisone (Millipore Sigma, H6909), 14 pg/mL Triiodo-L-thyronine (T3) (Millipore Sigma; T5516), 1% *N*-2 Supplement (Gibco; 17502048), and 250 μg/mL Taurine (Sigma; T0625) final concentration. For cell culture, plates are coated with 2.5 μg/mL vitronectin (STEMCELL Technologies; 07180), cells are plated at a concentration of 0.5 × 10^6^ cells/mL. Media is replaced every 2 days RPE are matured per manufacturers guidelines.

Primary HMRECs were purchased at P3 (Cell Systems; ACBRI 18) and cultured at 37°C in 5% CO2 in endothelial cell growth basal medium (EBM-2) (Lonza, CC-3156) supplemented with the Endothelial Growth Media (EGM)-2 SingleQuot kit (Lonza CC-4176) consisting of 0.04% hydrocortisone, 0.4% human fibroblast growth factor B (hFGF-B), 0.1% VEGF, 0.1% recombinant analog of insulin-like growth factor (R3 IGF-1), 0.1% ascorbic acid, 0.1% human epidermal growth factor (hEGF), 0.1% gentamicin/amphotericin solution (GA-1000) and 0.1% heparin. The media was also supplemented with 2% FBS (Atlanta Biologicals) 1% penicillin/streptomycin (Thermo Fisher Scientific; 15140122), and 1% L-glutamine (Lonza; BEBP17-605E). Transfections were performed at P7 in media omitting antibiotics for 48 h prior to being returned to normal media.

### CRISPR-Cas9 gene-editing of ARPE19s

CRISPR-Cas9 technology was utilized to introduce a specific point mutation at codon 345 of the EFEMP1 gene through a homology-directed repair approach. This mutation, R345W (arginine to tryptophan), has been associated with ADD, making it a valuable model for studying pathophysiological mechanisms in RPE cells.^49^ The EFEMP1 gene at the precise location of interest using the sgRNA-seq 5′-GACCACAAATGAATGCCGGG-3′ and the single-stranded oligodeoxynucleotide (ssODN): 5′–A∗T∗ATAAATGAGTGTGAGACCACAAATGAATGCTGGGAGGATGAAATGTGTTGGAATTATCATG∗G∗C – 3′. The sgRNA-seq is then introduced into ARPE19 cells, a human retinal pigment epithelial cell line, along with the Cas9 endonuclease. Following successful editing, the mutant cells are selected and validated using techniques such as PCR and sequencing to confirm the presence of the EFEMP1 R345W mutation. These mutant ARPE19 cells can then be used to study the molecular and cellular effects of the mutation, providing insights into retinal disease mechanisms and potential therapeutic strategies.

### ASOs synthesis

ASOs were synthesized using solid-phase phosphoramidite chemistry, incorporating phosphorothioate (PS) backbone and 2′-O-methoxyethyl (2′-MOE) sugar modifications. The ASOs were purified using reversed-phase high-performance liquid chromatography (HPLC) followed by desalting. ASOs utilized were endotoxin-free with greater than 80% purity and produced by Axolabs GmbH. The 20-nucleotide 5–10–5 gapmer ASOs (EFEMP1 ASO sequence: 5′-CTGTCAGCATCTCCAGGTCC-3′) and ASO mismatch (5′-CTGTCAGCCTCTTCAGGTCC-3′) were synthesized with a fully PS backbone. The central ten DNA nucleotides were flanked by five 2′MOE-modified ribonucleotides at each terminus. This configuration enables RNase H-mediated cleavage of the target RNA and confers enhanced nuclease resistance and metabolic stability. All 247 sequences and associated data can be accessed in patent (methods and compositions for targeting Efemp1: WO2023154964A1).

### RNA-seq library preparation and sequencing

RNA-seq analyses were performed for each condition with three biological replicates, each consisting of 100,000 cells. Total RNA was extracted from using the RNeasy Plus Mini Kit (QIAGEN; 74134), following the manufacturer’s instructions. RNA quality was assessed using a Tapestation and samples with an RNA integrity number (RIN) > 8 were used for sequencing. RNA libraries were constructed by Simches Research Center, Next Generation Sequencing Core, Massachusetts General Hospital (Boston, MA, USA) using a NEBNext Ultra II Directional RNA Library Prep Kit (Ipswich, MA, USA). The library was sequenced on the Illumina NextSeq 2000 sequencer platform. Sequences consisted of 50 bp paired-end reads to a minimum depth of 30 million reads per sample.

### RNA-seq data analysis

Raw read sequences were analyzed for quality using FastQC^50^ and MultiQC.^51^ Adapters and low-quality bases were trimmed using Cutadapt 3.4^52^ and Trim Galore 0.6.5-1 with the parameters -q 20 --phred33 --length 20 Babraham Bioinformatics-Trim Galore! Available online: https://www.bioinformatics.babraham.ac.uk/projects/trim_galore accessed in August 2024. Human genome GRCh38 was indexed using Hisat2 (2.1.0–4),^53^ incorporating splice junctions from Gencode GTF gencode.v.46.primary_assembly.annotation.gtf file.^54^ Stringtie 2.1.5^55^ and gencode GTF annotation gencode. v.46.primary_assembly.annotation.gtf was used to generate gene counts. DESeq2 1.34.0^56^ was used to determine differential expression. DEGs were defined by an adjusted *p* value ≤0.05, and log_2_ fold change (LFC) ≥ 1.5. For DEGs, the *p* value was adjusted using Benjamin-Hochberg correction individually for each pairwise comparison. Metascape, was used for GO analysis.^57^ All figures were generated in the R 4.4.1 environment.

### GSEA

The normalized count matrix obtained from DESeq2 was used for GSEA v.4.3.2.^51^^,^^58^ GSEA was performed using 1,000 permutations and gene set permutations with gene set size filters: min = 15 and max = 500. Both the Hallmark gene set and Reactome C2 gene set were used for analysis.

### Lentiviral processing and transduction

iPSc-derived RPE cells were plated and 24 h after seeding, the cells were incubated (MOI:5, 10, and 15) with lentiviral constructs under a CMV promoter the transductions were carried out in the presence of 8 μg/mL polybrene to enhance cellular uptake. After the transduction mix was added to the cells. Ater 24 h of treatment, the transduction is replaced with fresh media. Transduction efficiency was assessed using the EVOS M7000 imaging system (Thermo Fisher Scientific, Waltham, MA, USA) using the green fluorescent protein (GFP) channel at 20× magnification.

### RNA isolation and RT-qPCR

Unless otherwise stated, cells for quantitative reverse-transcription PCR (RT-qPCR) analysis were treated for 48 h before being lysed. RNA was extracted using RNeasy Micro Kits (QIAGEN; 74004) according to the manufacturer’s instructions. Transcription into complementary DNA was performed using the iScript cDNA synthesis kit (Bio-Rad; 1708890), following the manufacturers’ protocol, and probed using SsoAdvanced Universal SYBR Green Supermix (Bio-Rad; 1725274) in 384-well plates (Bio-Rad, HSP3805). Logarithm validated premixed primers for selected genes (Table S4) were purchased from Integrated DNA Technologies (Coralville, IA, USA). Quantification Cycle (*C*_*q*_) values were determined for control and experimental samples. Changes in mRNA expression were normalized to housekeeping genes including beta2 microtubulin (B2M), mRNA expression using the 2^−ΔΔ*Cq*^ calculations using CFX Maestro Software 2.3 (Bio-Rad; 12013758). RNA was extracted and purified using a RNeasy Plus Micro kit (QIAGEN; 74034). Briefly, eye posterior chamber in RLT buffer supplemented with 3% of β-mercaptoethanol (Millipore Sigma; 444203) was lysed with a hand homogenizer. Samples were centrifuged at maximum speed and supernatant was collected for RNA purification as per manufacturer instructions. RNA concentration was measured by Nanodrop.

### Western blotting

A total of 20 μg of cell lysates were prepared in 4 μL of 1 M DTT (Millipore Sigma; 646563) and 10 μL Laemmli buffer (Boston BioProducts; BP-110R) to a final volume of 40 μL and denatured with heat for 5 min at 95°C. Samples were fractionated for 70 V for 15 min and further for 100 V for 1 h using Criterion precast 4%–20% gels (Bio-Rad; 5671093) and Tris-Glycine-Sodium Dodecyl Sulfate (Tris-Glycine-SDS) running buffer (Bio-Rad; 610771EDU). For multiplexing applications, proteins were transferred to polyvinylidene fluoride membranes (PVDF) (Thermo Fisher Scientific; IB23001), using the iBlot 2 dry transfer system (Thermo Fisher Scientific; IB21002S). Total protein levels for this application were evaluated using Revert 700 Total Protein Stain (Thermo Fisher Scientific; NC1683155) according to the manufacturer’s instructions. Membranes were blocked for 1 h with Intercept Blocking Tris-Buffered Saline (TBS) Buffer (LI-CORbio; 927–60,001) for 2 h. Membranes were then incubated in primary antibodies in blocking buffer for either 2 h at room temperature or 18 h at 4°C (Table S5). Blots were washed thrice with TBS-Tween 20 (TBS-T) buffer for 10 min (Thermo Fisher Scientific; 28360) and incubated with secondary antibodies in blocking buffer for 1 h at room temperature (Table S5). Immunoreactive bands were detected using the Odyssey Infrared Imaging System (LI-CORbio; Lincoln, NE, USA) and visualized on the Image Studio Acquisition Software (v.2.1; LI-CORbio). For blots analyzed using chemiluminescence, blots were transferred to PVDF using the *trans*-blot Turbo transfer system (Bio-Rad; 1704150) and *trans*-blot Turbo RTA Midi PVDF Transfer Kit (Bio-Rad, 1704273) according to the manufacturer’s instructions. Membranes were reversibly stained to assess protein loading (Thermo Fisher Scientific; 24585), blocked in 5% milk in TBS for 1 h at repetition time (RT) and incubated overnight at 4°C in the appropriate primary antibodies in blocking buffer (Table S5). Blots were incubated with the corresponding HRP-conjugated secondary antibodies (Table S5) at RT and washed as described above. Blots were developed using Clarity ECl Western Substrate (Bio-Rad; 1705061) and visualized using an iBright FL1500 imaging system (Thermo Fisher Scientific; A44241).

### Immunocytochemistry

iPSC-RPE cells were seeded on glass slides (Mat-Tek; CCS-4), 40,000 cells per well. Cells were reverse transfected with EFEMP1-targeted ASOs, in serum free media and incubated for 2 h. The media was exchanged with 500 μL growth media and incubated for 24 h at 37°C. Cells were washed with ice-cold PBS, fixed in 4% paraformaldehyde (PFA) for 5 min, washed in PBS and incubated in permeabilization and blocking buffer (PB) consisting of 1× PBS, 1% Normal Donkey Serum (Millipore Sigma; D9663) and 0.05% Triton X-100 (Millipore Sigma; T8787) overnight. Cells were then incubated with primary antibody (Table S5) diluted in blocking buffer overnight at 4°C. Samples were washed in PBS and incubated with the corresponding secondary antibody (Table S5) for 2 h at room temperature, and nuclei were stained with DAPI (1:5,000) for 15 min. Finally, samples were mounted with Vectashield mounting media (Vector Laboratories; H-1000). Images were acquired with a Leica SP8 confocal microscope (Leica Camera AG, Wetzlar, Germany).

### Watershed cell segmentation

Image processing was performed using MATLAB R2021a. The watershed transform was applied to Gaussian filtered images, which we also imposed local minima on to avoid over-segmentation. Images of the cell membranes were smoothed using a Gaussian filter with a sigma of 3 pixels. Local minima were generated via the H-minima transform (using the MATLAB function imextendedmin with a minimum depth of 4 intensity units).

### Human choroidal explants

The Schepens Eye Research Institute Institutional Review Board reviewed the experiments conducted on postmortem human eye material and deemed them exempt from institutional review board (IRB) approval (REDCap ID#910). This exemption was granted as the research does not involve human subjects. The postmortem donor eye globes were procured from the Minnesota Lions Gift of Sight Eye Bank (Saint Paul, MN, USA). Cultrex Basement Membrane Extract (BME) (R&D Systems; 3432-010-01) was thawed on ice for 24 h prior to the experiment. Postmortem human eyes, provided by the Lions Gift of Sight Eye Bank, were obtained within 24 h and immediately dissected. Eyes were cut circumferentially about 1.0 mm posterior to the limbus and the cornea, lens, iris, retina, conjunctiva, and sclera were removed leaving the RPE/choroid behind. The remaining RPE/choroid was transferred to explant culture medium composed of media composed of MCDB 131 (Thermo Fisher Scientific; 10372-019), EGM 2 SingleQuot Kit supplement and Growth factors (Lonza; CC-4176), 12% FBS (Atlanta Biologicals; S11150), 1% Penicillin/Streptomycin (Lonza; 17-602E), and 1% L-glutamine (Lonza; BE17-605E/U1). Using a 1 mm biopsy punch (Thermo Fisher Scientific; 12-460-401), 1 mm circular punches were generated circumferentially, equidistant from the optic disk. Punches were then embedded in 30 μL domes of Cultrex BME in 24-well tissue culture plates (CellTreat; 229124) and allowed to set for 45 min at 37°C, 5% CO_2_. Around 500 μL of media was gently added to each explant, incubating at 37°C, 5% CO_2._ Upon sprouting, growth medium was exchanged for media omitting antibiotics. Transfections were performed using 25 nM ASO mismatch control (M2) or EFEMP1 ASO diluted in optiMEM reduced serum media (ThermoFisher Scientific; 31985070). Diluted constructs were packaged using Lipofectamine RNAiMAX Transfection reagent (Thermo Fisher Scientific; 13778150) in optiMEM, and administered to the explants for 48 h before the media was exchanged with normal growth medium. Following transfection, the outgrowth of the explants was monitored by bright field microscopy using a bright-field microscope (Olympus CKX53, Olympus, Tokyo, Japan). Media was changed and bright field images were taken every other day for 12 consecutive days. Transfections were repeated between days 6 and 7 to maintain the knockdown effect. To further observe 3D outgrowth and demonstrate viability, explants were also stained with 1 mM Calcein AM cell-permeable dye (Thermo Fisher Scientific; C34852) on day 12. Stained explants were observed using contiguous rectangular regions at 10× magnification with a confocal microscope (Leica SP8, Leica, Wetzlar, Germany). Images from each region were taken at a depth of ∼6–8 μm. Bright field and fluorescent images were quantified using FIJI-ImageJ2 software (National Institutes of Health, Bethesda, MD, USA) and the SWIFT-Choroid computerized quantification method.^28^

### Mouse choroidal explants

Mouse choroidal explants were prepared using an established *ex vivo* choroidal sprouting assay with minor modifications.^28^^,^^59^ Eyes were enucleated from C57BL/6J mice and immediately transferred to ice-cold sterile PBS. Under a dissecting microscope, the anterior segment, lens, retina, and vitreous were carefully removed to isolate the posterior eyecup containing the RPE/choroid/sclera complex. The RPE/choroid was separated and cut into 1 mm circular punches using a sterile biopsy punch. Mouse explants were then treated as described earlier.

### Intravitreal injections

Experiments utilizing live vertebrates were reviewed and approved by the Institutional Animal Care and Use Committee (Schepens Eye Research Institute) and carried out according to the National Institutes of Health Guide for the Care and use of Laboratory Animals. All studies were performed in compliance with ARRIVE guidelines. Mice were anesthetized by intraperitoneal (i.p.) injection of a ketamine/xylazine mixture. A single intravitreal injection of either ASO mismatch or EFEMP1 ASO animals was administered. Animals were euthanized at predefined timepoints and samples were collected for further analysis.

### Statistics

Western blots were quantified using ImageJ software v.13 (National Institutes of Health). Results are expressed as mean ± standard error of the mean (SEM). Statistical analyses were performed using GraphPad Prism v.10.1.2 software, with *p* values < 0.05 defined as statistically significant. Residuals of raw data were initially tested for normality to determine whether to apply parametric or nonparametric tests. Parametric data were analyzed overall with the Student’s *t* test or one-way or two-way ANOVA followed by Tukey’s test for multiple comparisons unless stated otherwise. Rank correlation was measured by Pearsons’s rank correlation coefficient. Nonparametric data were analyzed by Mann-Whitney *U* test, Kruskal-Wallis or Friedman’s test conducted with Dunn’s post hoc test for multiple comparisons. Rank correlation for nonparametric data was performed using Spearman’s rank correlation coefficient.

## Data and code availability

The bioinformatics data discussed in this publication have been deposited in NCBIs Gene Expression Omnibus^60^ and are accessible through GEO Series accession number GSE289976 (https://www.ncbi.nlm.nih.gov/geo/query/acc.cgi?acc=GSE289976). All code or data will be made available upon reasonable request to the authors.

## Acknowledgments

We would like to thank the 10.13039/100007745Massachusetts Lions Eye Research Fund for supporting this research through the young investigator program awarded to M.O.H. We would also like to acknowledge the Schepens Eye Research Institute (SERI) morphology core for their services (supported by 10.13039/100000053National Eye Institute core grant P30EYE003790) and thank the Enterprise Research Infrastructure & Services (ERIS) at 10.13039/100007806Mass General Brigham for their in-depth support and for the provision of computational resources.

## Author contributions

Project conceptualization was performed by A.P., V.V., C.L., and M.O.H.; methodology for experimental approaches was established by W.P.M., S.A.-A., P.P.-C., A.L., S.A., C.L., and M.O.H.; investigations were carried out by W.P.M., S.A.-A., P.P.-C., R.L., A.U., J.S.-S., T.E.V., C.M., D.S., M.S., J.V., A.M.-T., X.H., Y.A.-R., B.A.K., K.W.B., A.L.G., A.V., R.S., and M.O.H.; visualization of experimental endpoints was performed M.O.H., W.P.M., S.A.-A., P.P.-C., R.L., A.U., J.S.-S., T.E.V., D.S., M.S., J.V., B.A.K., K.W.B., A.L.G., A.V., R.S., A.L., and M.O.H.; funding was acquired by M.O.H.; project administration was the responsibility of M.O.H. and C.L.; research was supervised by M.O.H.; the manuscript was written by W.P.M., S.A.-A., P.P.-C., and M.O.H.; the manuscript was reviewed and edited by W.P.M., S.A.-A., P.P.-C., C.M., A.L., L.A.K., J.F.A.-V., A.P., S.A., V.V., C.L., and M.O.H.

## Declaration of interests

A.P., S.A., and C.L. are co-founders and shareholders of Aldebaran Therapeutics.

## Supplemental information

Document S1. Figures S1–S10 and Tables S3–S5, and Supplemental Materials and Methods

Table S1. ARPE19 vs. ARPE19 R345W GO terms

Table S2. HMREC GO terms

Document S2. Article plus supplemental information
