# Supplementary material for: Development of an antisense oligonucleotide targeting EFEMP1 in models relevant to macular degeneration
Source: Mol Ther Nucleic Acids. 2026 Jul 21;37(3):103024. doi: 10.1016/j.omtn.2026.103024 (PMC13469845; doi:10.1016/j.omtn.2026.103024)
Supplement: Supplementary file 1 [file mmc1.pdf]

OMTN, Volume *37*

Supplemental information

Development of an antisense oligonucleotide targeting EFEMP1 in models relevant to macular degeneration

William P. Miller, Said Arevalo-Alquichire, Paula Perez-Corredor, Rose Lin, Anil Upreti, Jeysson Sanchez-Suarez, Tim E. Vanderleest, Claudia Marino, Donovan Spencer, Matthew Scribner, Jebgy Vargas, Andres Muriel-Torres, Xinyao Hu, Yvonne Adu-Rutledge, Bryan A. Kaplan, Karim W. Barake, Audrey L. Gunawan, Aruvi Vijikumar, Reeya Shrestha, Anton Lennikov, Leo A. Kim, Joseph F. Arboleda-Velasquez, Andrew Peterson, Sudhir Agrawal, Vinod Vathipadiekal, Carlos Loya, and Michael O'Hare

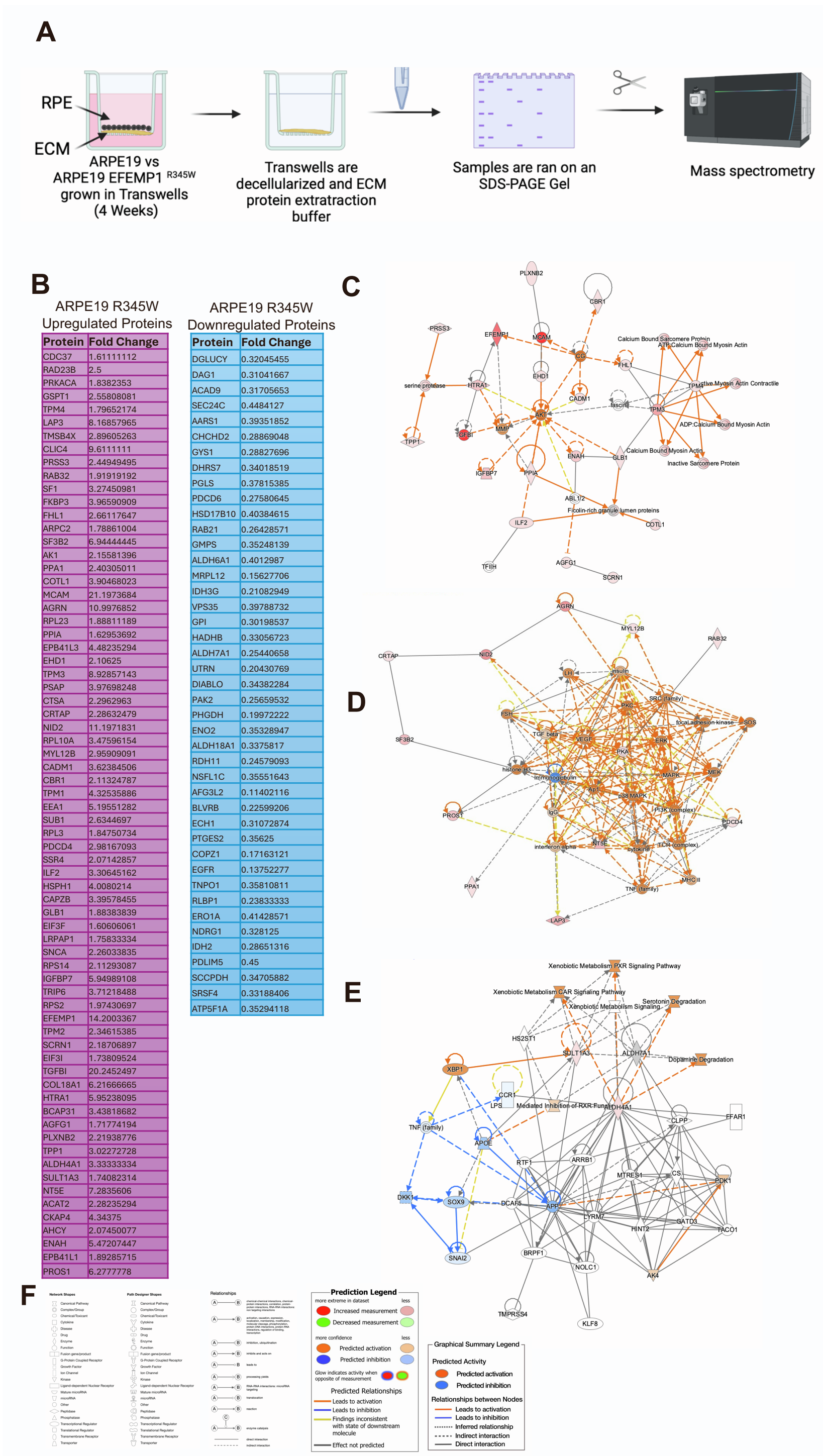

ARPE19 R345W Upregulated Proteins

Protein	Fold Change
CDC37	1.61111112
RAD23B	2.5
PRKACA	1.8382353
GSPT1	2.55808081
TPM4	1.79652174
LAP3	8.16857965
TMSB4X	2.89605263
CLIC4	9.6111111
PRSS3	2.44949495
RAB32	1.91919192
SF1	3.27450981
FKBP3	3.96590909
FHL1	2.66117647
ARPC2	1.78861004
SF3B2	6.94444445
AK1	2.15581396
PPA1	2.40305011
COTL1	3.90468023
MCAM	21.1973684
AGRN	10.9976852
RPL23	1.88811189
PPIA	1.62953692
EPB41L3	4.48235294
EHD1	2.10625
TPM3	8.92857143
PSAP	3.97698248
CTSA	2.2962963
CRTAP	2.28632479
NID2	11.1971831
RPL10A	3.47596154
MYL12B	2.95909091
CADM1	3.62384506
CBR1	2.11324787
TPM1	4.32535886
EEA1	5.19551282
SUB1	2.6344697
RPL3	1.84750734
PDCD4	2.98167093
SSR4	2.07142857
ILF2	3.30645162
HSPH1	4.0080214
CAPZB	3.39578455
GLB1	1.88383839
EIF3F	1.60606061
LRPAP1	1.75833334
SNCA	2.26033835
RPS14	2.11293087
IGFBP7	5.94989108
TRIP6	3.71218488
RPS2	1.97430697
EFEMP1	14.2003367
TPM2	2.34615385
SCRN1	2.18706897
EIF3I	1.73809524
TGFBI	20.2452497
COL18A1	6.21666665
HTRA1	5.95238095
BCAP31	3.43818682
AGFG1	1.71774194
PLXNB2	2.21938776
TPP1	3.02272728
ALDH4A1	3.33333334
SULT1A3	1.74082314
NT5E	7.2835606
ACAT2	2.28235294
CKAP4	4.34375
AHCY	2.07450077
ENAH	5.47207447
EPB41L1	1.89285715
PROS1	6.2777778

ARPE19 R345W Downregulated Proteins

Protein	Fold Change
DGLUCY	0.32045455
DAG1	0.31041667
ACAD9	0.31705653
SEC24C	0.4484127
AARS1	0.39351852
CHCHD2	0.28869048
GYS1	0.28827696
DHRS7	0.34018519
PGLS	0.37815385
PDCD6	0.27580645
HSD17B10	0.40384615
RAB21	0.26428571
GMPS	0.35248139
ALDH6A1	0.4012987
MRPL12	0.15627706
IDH3G	0.21082949
VPS35	0.39788732
GPI	0.30198537
HADHB	0.33056723
ALDH7A1	0.25440658
UTRN	0.20430769
DIABLO	0.34382284
PAK2	0.25659532
PHGDH	0.19972222
ENO2	0.35328947
ALDH18A1	0.3375817
RDH11	0.24579093
NSFL1C	0.35551643
AFG3L2	0.11402116
BLVRB	0.22599206
ECH1	0.31072874
PTGES2	0.35625
COPZ1	0.17163121
EGFR	0.13752277
TNPO1	0.35810811
RLBP1	0.23833333
ERO1A	0.41428571
NDRG1	0.328125
IDH2	0.28651316
PDLIM5	0.45
SCCPDH	0.34705882
SRSF4	0.33188406
ATP5F1A	0.35294118

Figure S1: Proteomic analysis of extracellular matrix from ARPR19 and ARPE19 R345W cells. A) Schematic of the experimental design for extracellular matrix processing of ARPE19 and

ARPE19 R345W cells for mass spectrometry analysis. **B**) Lists of proteins over 1.5-fold increased (red) in ARPE19 R345W and downregulated below 0.5-fold (blue). (n=2 per group) **C-E) I**ngenuity Pathway Analysis (IPA) networks of interest **F)** Key for IPA analysis.

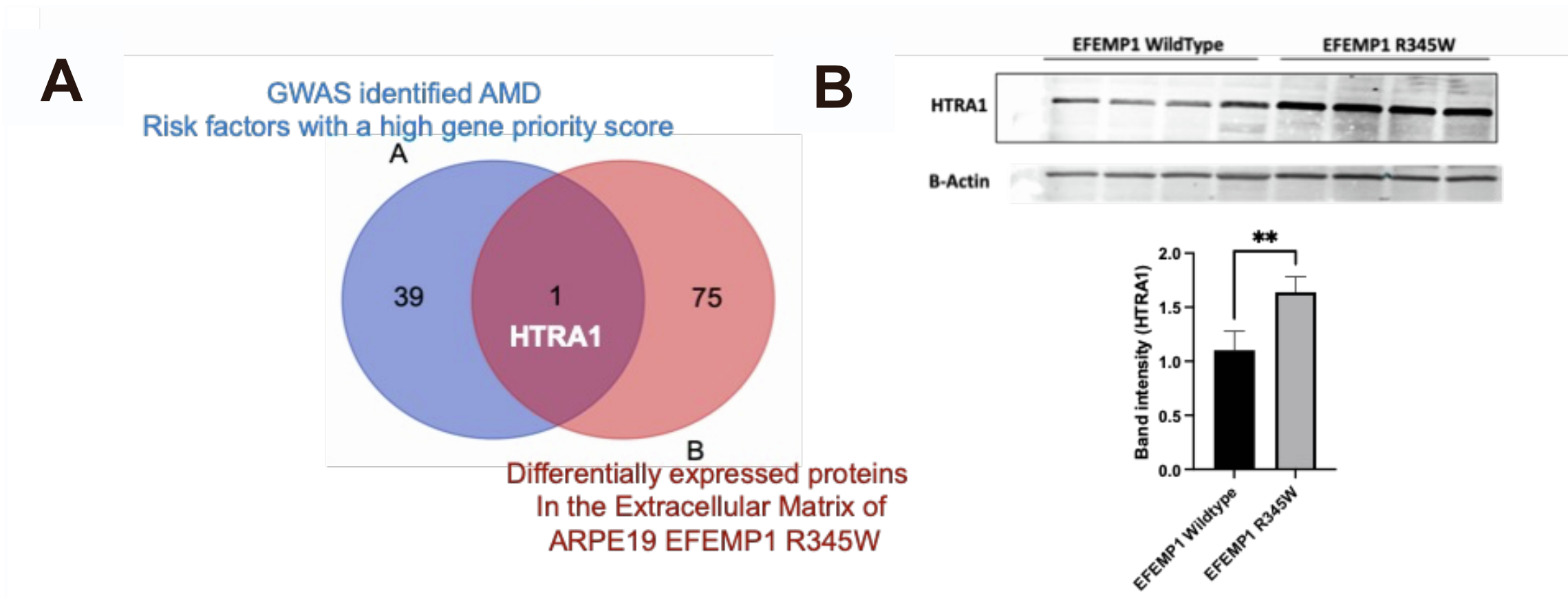

Figure S2: HTRA1 is upregulated in the extracellular matrix of ARPE19 R345W cells. A) Venn diagrham of GWAS identified risk factors with a high gene prioirty score compared with top changing proteins in the extracellular matrix of the ARPE19 R345W cells; HTRA1 is the only common target shared. **C)** HTRA1 protein levels are changing significantly in ARPE19 R345W cells (n=4), Students T-test, p<0.01 **.

A

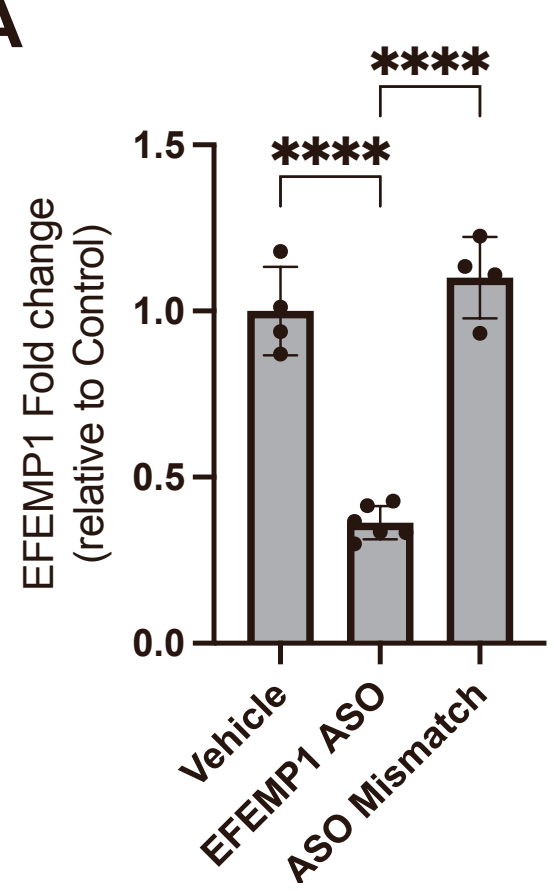

Figure S3: A) Antisense oligonucleotide with two mismatched nucleotides shows no effect on EFEMP1 expression in iPSC-RPE cells (n=4-6), one-way ANOVA, p<0.0001 ****.

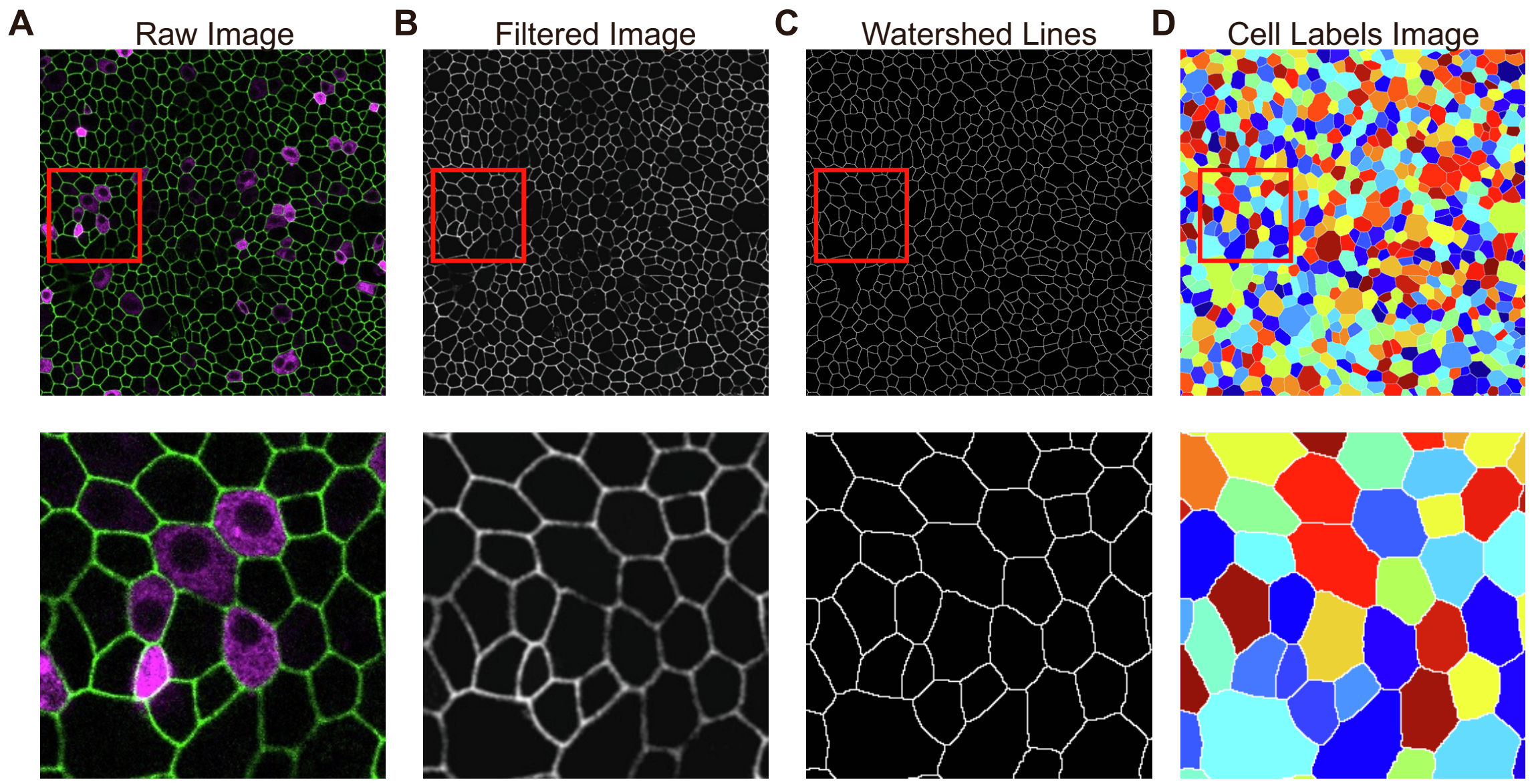

Figure S4: Image segmentation methodology. A) Raw 2-channel image of both Zo-1 (green) and EFEMP1 (magenta). **B)** The raw Zo-1 image was Gaussian filtered and local minima were suppressed using an h-minima transform. **C)** The watershed transform was applied to the filtered image to find the boundary lines of the cells (binary image). **D)** The watershed result was used to uniquely label each cell, shown here through assigning each cell a unique color. **A'-D')** Zoomed-in images of **(A-D)** for the ROI outlined by the red boxes.

A

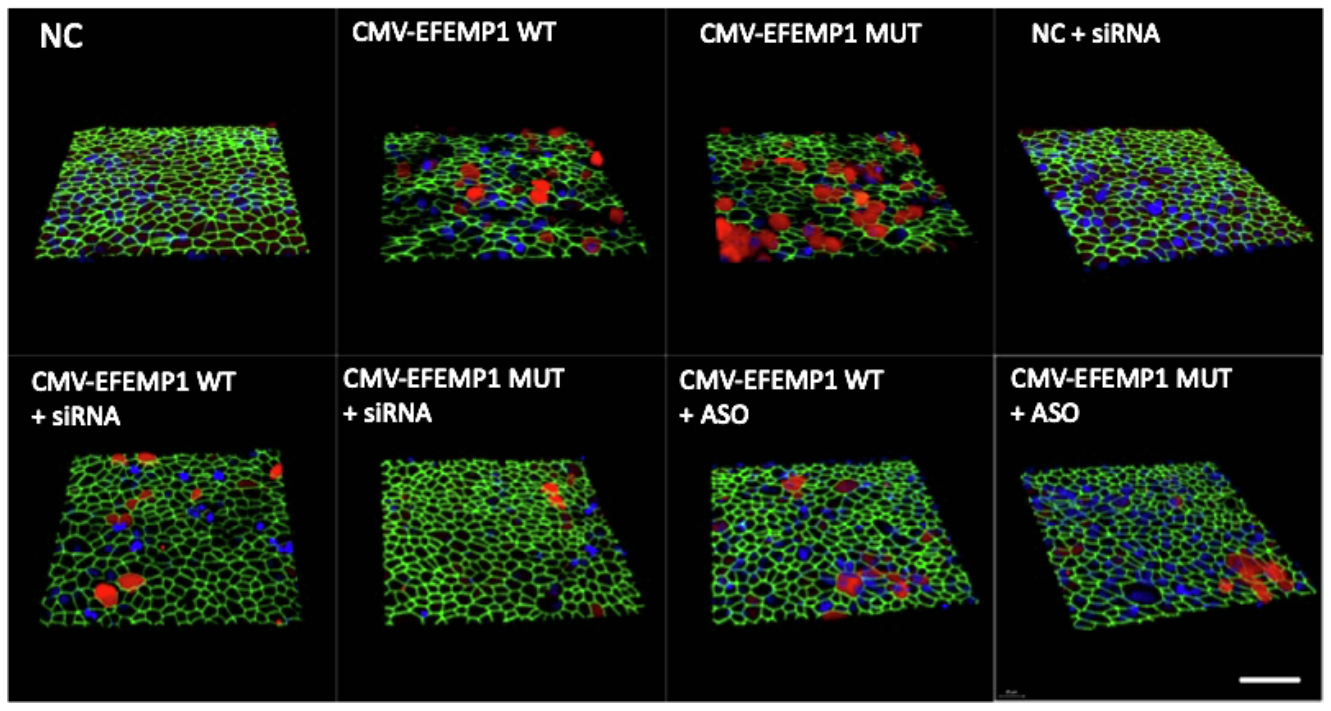

B

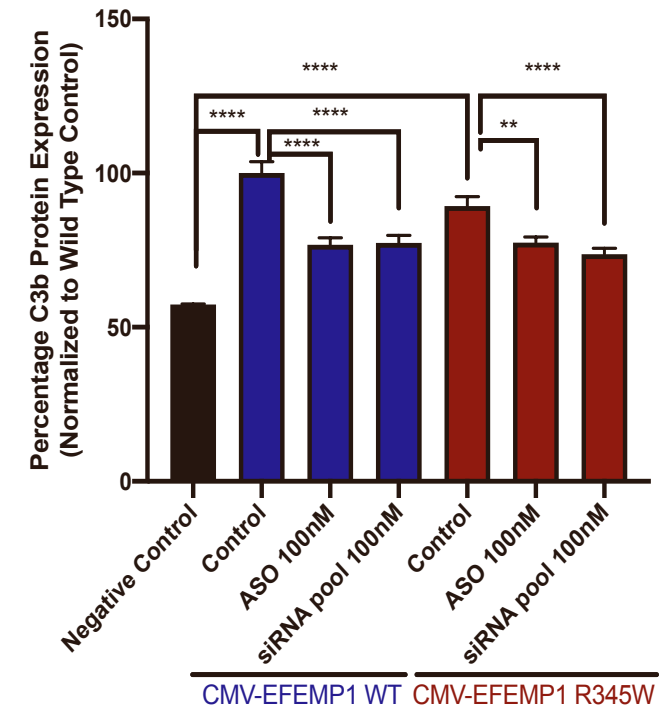

Figure S5: Representative 3D confocal images in iPSC-RPE showing after Lentiviral overexpression of either wild type EFEMP1 and EFEMP1R345W (N=4). A) C3/C3b increased significantly after overexpression of EFEMP1 (x63). iPSC-RPE cells overexpressing EFEMP1 were treated with an ASO Gapmer 100nM or a pool of EFEMP1 siRNAs 100nM to measure knockdown of EFEMP1 and the effect on C3/C3b. C3/C3b: marked in red Scale bar 40μm B) Data was analyzed using a one-way ANOVA. (**) $p < 0.01$, (****) $p < 0.0001$

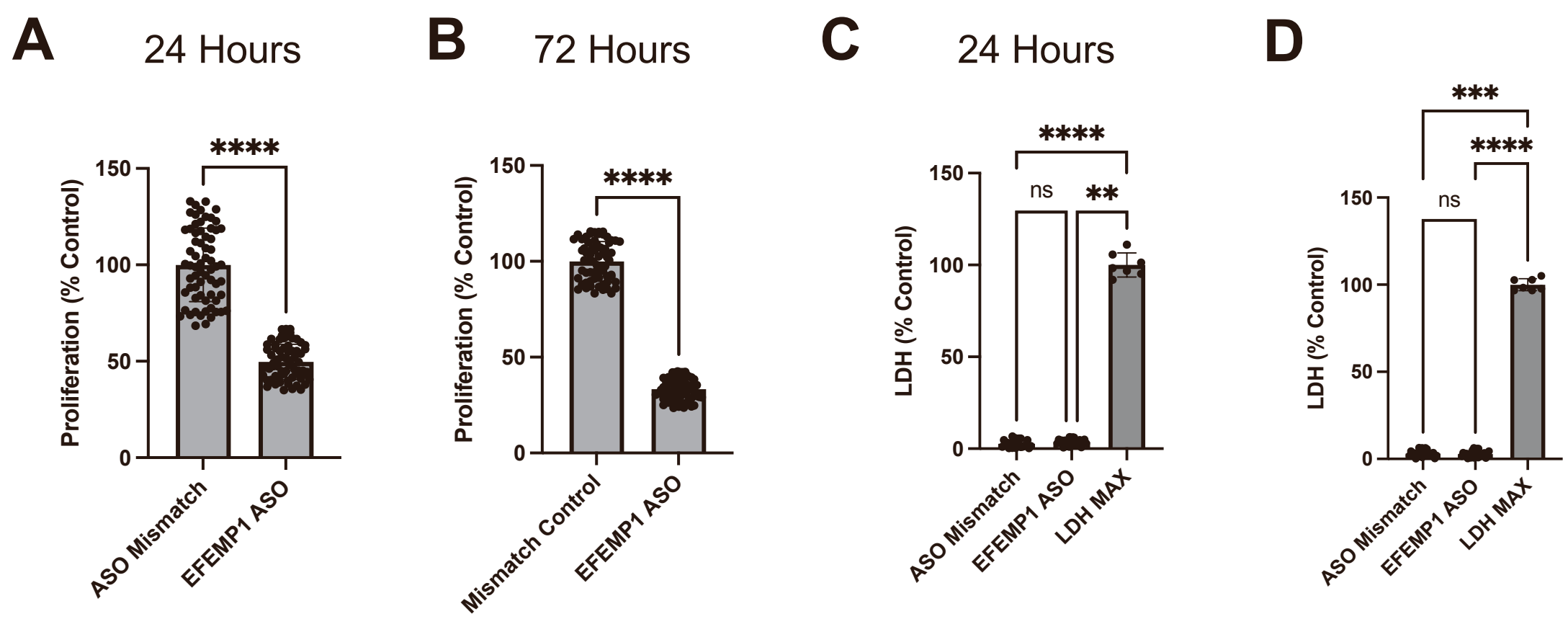

Figure S6: Proliferation in HMRECs at **A)** 24 and **B)** 72 hours after EFEMP1 ASO transfection measured by cyQUANT proliferation assay (n=24), Mann-Whitney test, $p<0.0001$ ****. LDH assay shows no cytoxicty of EFEMP1 ASO at **C)** 24 hours and **D)** 72 hours. (n=8-24), Kushal Wallis test, $p<0.01$ **, $p<0.001$***, $p<0.0001$ ****.

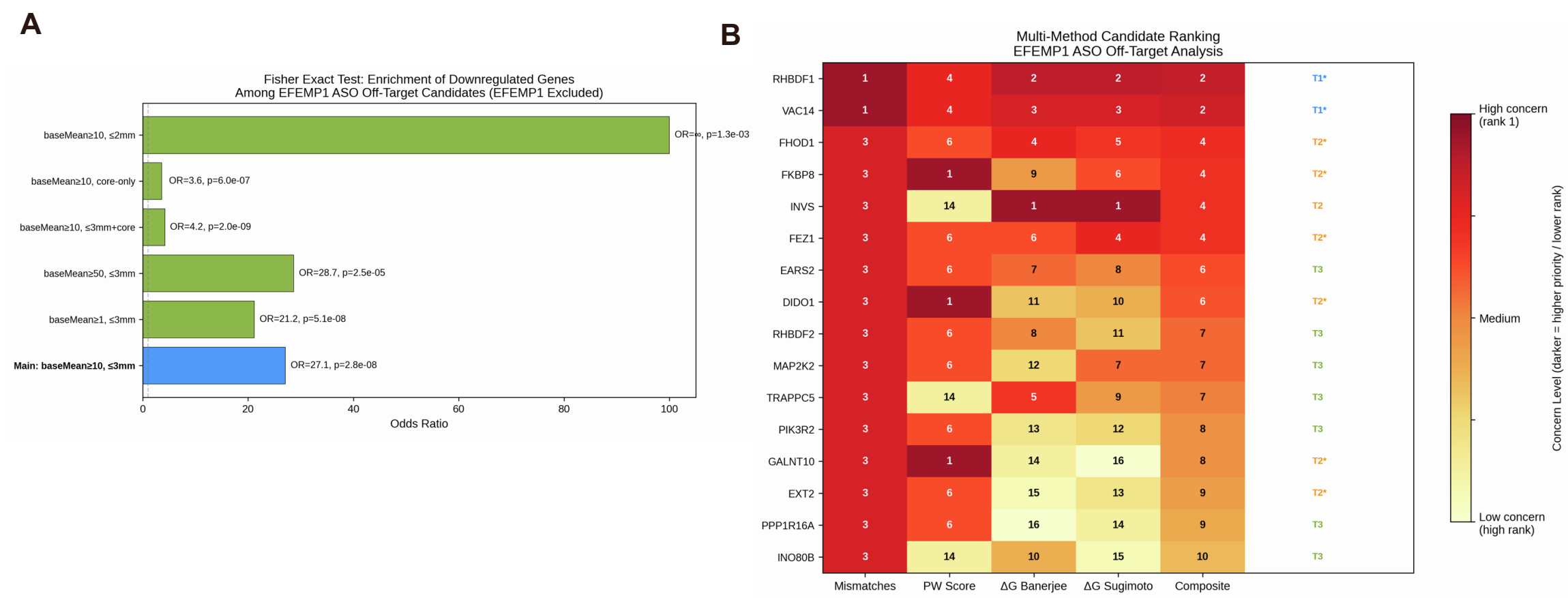

Figure S7: In silico assessment of potential EFEMP1 ASO off-target effects.

A) Fisher exact test enrichment analysis evaluating whether predicted EFEMP1 ASO off-target candidate sets were overrepresented among genes significantly downregulated after EFEMP1 ASO treatment compared with ASO mismatch control. Candidate sets were defined by full 20-mer sequence similarity, central gapmer-core matching, and combined criteria. EFEMP1 was excluded from all off-target enrichment analyses because it represents the intended on-target transcript. The primary expressed-gene background was defined as genes with DESeq2 baseMean ≥10 and non-missing differential-expression statistics. **B**) Multi-method prioritization of candidate off-target genes based on full-length mismatch count, gap/wing position-weighted mismatch score, predicted DNA:RNA duplex free energy, and RNA-seq differential expression. RHBDF1 and VAC14 were the highest-priority candidates because they had only two mismatches to the EFEMP1 ASO target sequence, relatively stable predicted DNA:RNA duplexes, and significant downregulation. These computational predictions identify candidate sequence-dependent off-targets but do not prove direct RNase-H-mediated cleavage.

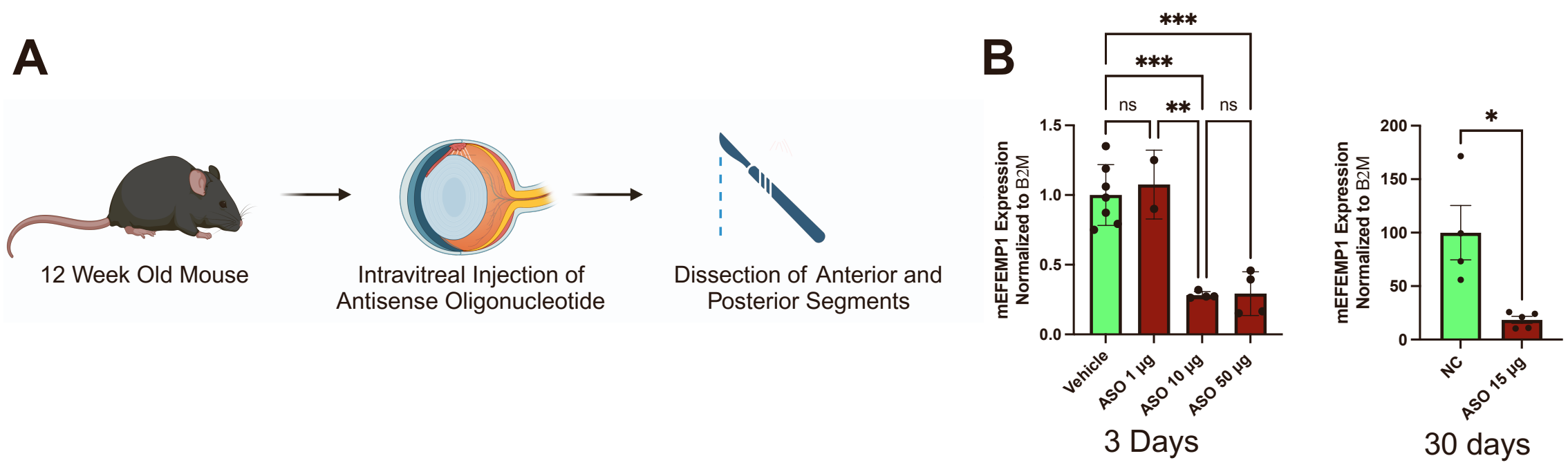

Figure S8: A) Schematic of ASO intravitreal injection in C57BL6/J mice with EFEMP1 targeted ASO and dissection of the eye into anterior and posterior segments for molecular analysis. **B)** qPCR showing EFEMP1 mRNA expression in the mouse posterior segment at 3 days after IVT injection of EFEMP1 targeted ASOs at 1, 10 and 50µg or after 30 days at 15µg. (n=2-6) one-way ANOVA or Students T-test, $p<0.05$ *, $p<0.01$ **, $p<0.001$ ***.

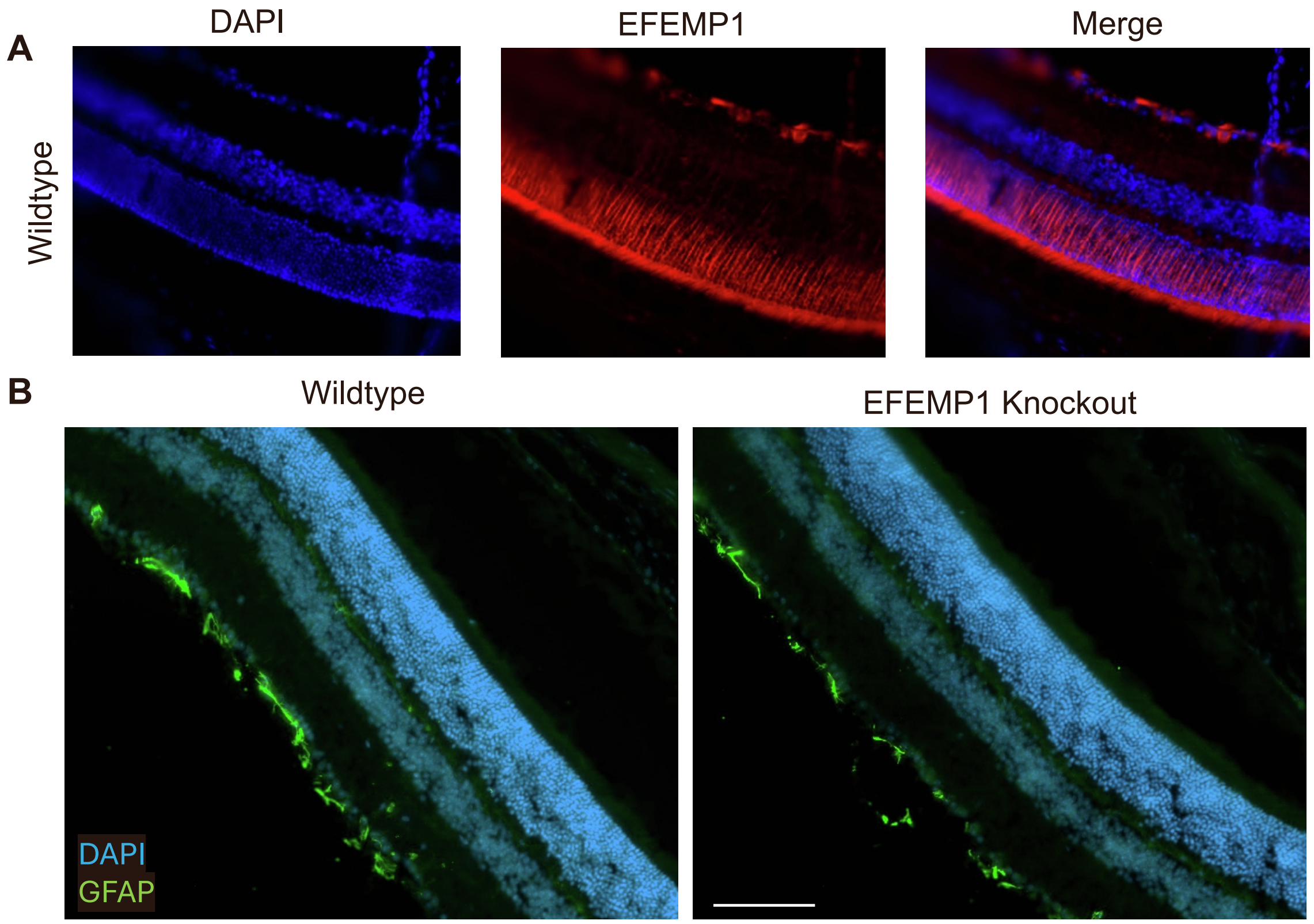

Figure S9: A) Representative images showing EFEMP1 expression in muller cells of retina in C57BL6J mice using Leica Sp8 confocal imaging**. B)** Representative images of Glial Fibrillary Acidic Protein (GFAP; green) and DAPI (blue) staining in the retina of wild type and EFEMP1 knockout mice. (Scale 100μM)

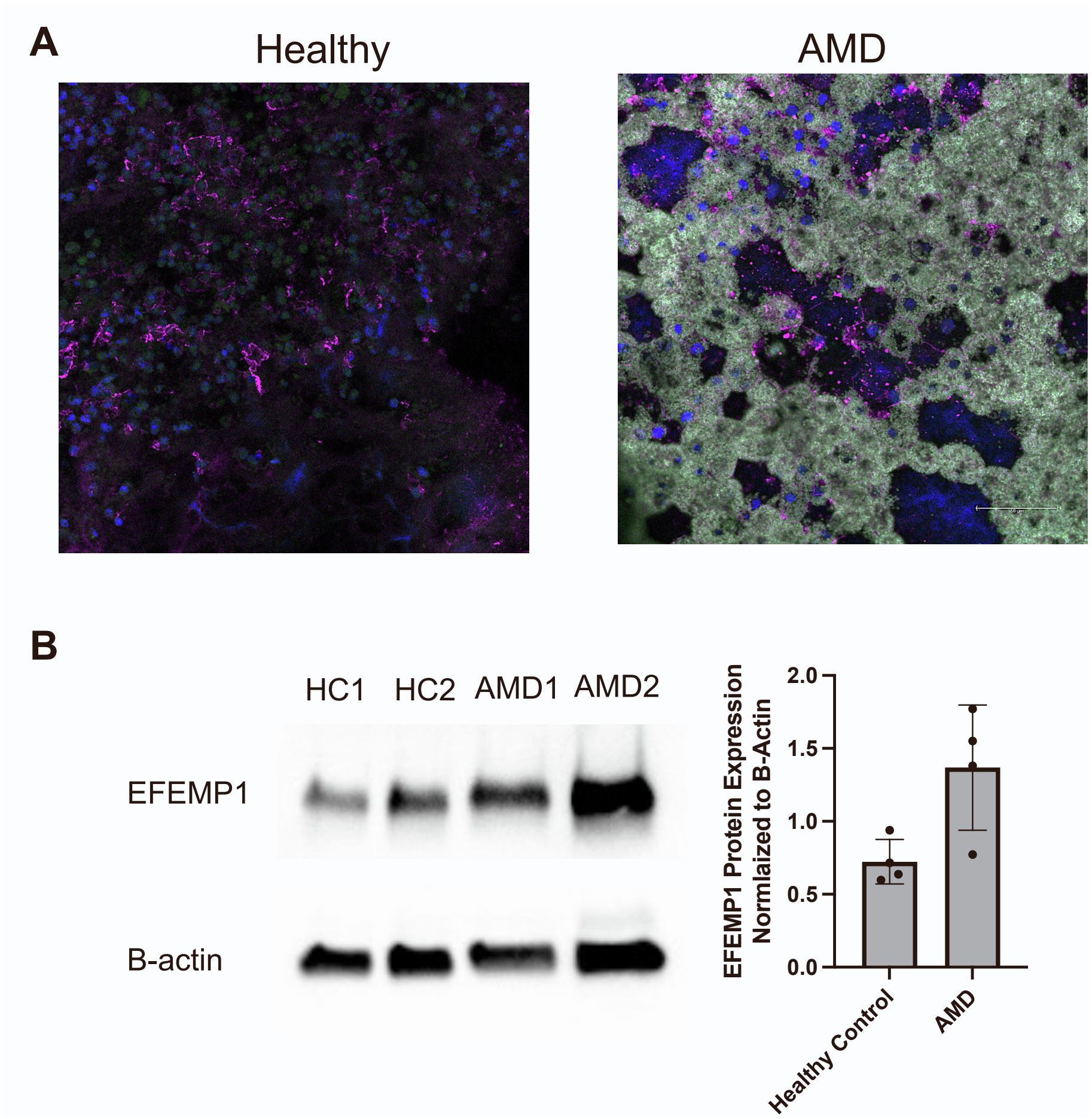

Figure S10: EFEMP1 is overexpressed in AMD tissue. A) Immunofluroscence staining of healthy donor RPE/choroids and AMD RPE/choroids with antibodys against EFEMP1 (Green), TIMP3 (magenta) and DAPI (blue). Scale Bar: 50μm **B)** Western blotting of healthy and AMD RPE/choroid lystaes showing increased EFEMP1 levels in AMD eyes (n=4).

Table S1: ARPE19 vs ARPE19 R345W GO terms**.**

Table S2: HMREC GO terms.

Table S3. Post-mortem RPE/Choroid Tissue Donor Demographics

Patient #	Age	Gender	Cause of Death	Past Ocular History
**1**	53	M	Anoxic Brain Injury	Healthy
**2**	66	F	COPD	Healthy
**3**	58	M	Cardiac arrest	Healthy
**4**	68	F	End stage COPD	Healthy
**5**	80	F	Heart Failure	AMD
**6**	85	M	Metastatic small cell ancer	AMD
**7**	80	F	Heart Failure	AMD

Table S4. Summary of forward and reverse primer sequences.

Target	Species	Forward	Reverse
Efemp1	Mouse	5' CAT GAG CAT CCG ATC TGA CA 3'	5' GGC TTC TTT GTC GTA GGT AGA 3'
B2m (House keeping)	Mouse	5' TGG TCT TTC TGG TGC TTG TC 3'	5' GGG TGG AAC TGT GTT ACG TAG 3'
EFEMP1	Human	5' CAA CCC TTC CCA CCG TAT C 3'	5' CAC ACT TGG TCT GCT CTA CAG 3'
C3	Human	5' GCC TTT GTT CTC ATC TCG CT 3'	5' AGT CTC CTG CTT TAG TGA TGC 3'
B2m (House keeping)	Human	5' GGA CTG GTC TTT CTA TCT CTT GT 3'	5' ACC TCC ATG ATG CTG CTT AC 3'

Table S5. Summary of primary and secondary antibodies sources and dilutions.

Target	Primary Antibody	Company (Cat No.)	Dilution (application)
EFEMP1	Rabbit mAb	Abcam (ab246547)	1:250 (ICC), 1:1000 (WB)
EFEMP1	Mouse mAb	Santa Cruz (sc-33722)	1:200 (IF)
β-actin	Mouse mAb	Cell Signalling (3700S)	1:1000 (WB)
β-actin	Rabbit mAb	Cell Signalling (4970S)	1:1000 (WB)
HIF1α	Rabbit mAb	Cell Signalling (36169S)	1:1000 (WB)
TIMP3	Rabbit pAb	ThermoFisher Scientific (710404)	1:200 (IF)
**Secondary Antibody**		**Company (Cat No.)**	**Dilution (application)**
IRDye 800CW anti-mouse		Licor (926-32212)	1:2000 (WB)
IRDye 680R anti-rabbit		Licor (926-88071)	1:2000 (WB)
Anti-rabbit IgG HRP-conjugated		R&D Systems (HAF008)	1:2000 (WB)
Donkey anti-rabbit AlexaFluor 647		ThermoFisher Scientfic (A-31573)	1:1000 (ICC/IF)
Donkey anti-mouse AlexaFluor 594		ThermoFisher Scientfic (A-21203)	1:1000 (ICC/IF)

Supplemental Materials and Methods:

Extracellular matrix processing: We compared the extracellular matrix composition of ARPE19 and ARPE19 R345W after 4 weeks of differentiation in transwells. The transwells were decellularized by incubating them with sterile 0.5% Triton X-100 (Sigma; T8787-50ML) + 20 mM NH_4OH in PBS (Thermofischer; AM9624) 1.5 ml was added to the bottom chamber and 0.5 ml to the apical chamber) for 5 min at 37 °C, followed by three washes with PBS. The extracellular matrix was collected in M-PER (Thermofischer; 78503) buffer and then prepared in native gel buffer and ran on a poly-acrylamide gel followed by silver staining (Thermofischer; 24612).

Mass spectrometry: Proteins from the extracellular matrix were extracted using M-PER Mammalian Protein Extraction Reagent (Thermo Fisher Scientific; 78501) according to the manufacturer's instructions. Samples in solution were subsequently processed and analyzed as a fee-for-service at the Taplin Biological Mass Spectrometry Facility (Harvard Medical School, Boston, MA, USA). Prior to mass spectrometric analysis, detergent was removed from protein samples using detergent removal spin columns. Peptide and protein identification was then performed by searching acquired spectra against a reference database of known peptide sequences using Sequest (Thermo Fisher Scientific), with standard parameters for precursor and fragment ion mass tolerance, and a false discovery rate (FDR) threshold of ≤1% applied at the peptide and protein levels.

In silico EFEMP1 ASO off-target analysis: Potential sequence-dependent off-target effects of the EFEMP1 ASO were evaluated using an in silico transcriptome-complementarity analysis. The EFEMP1 ASO sequence, 5′-CTGTCAGCATCTCCAGGTCC-3′, was analyzed against GENCODE v46 transcript sequences. Because the ASO binds RNA in an antiparallel orientation, the corresponding sense transcript target motif, 5′-GGACCTGGAGATGCTGACAG-3′, was used as the query sequence for transcriptome scanning. Transcript sequences were represented in DNA alphabet for computational analysis. For each

transcript, a 20-nucleotide sliding window was advanced one nucleotide at a time across the sense transcript sequence, and the number of mismatches between each window and the query motif was counted. Each transcript was assigned the minimum mismatch count observed across all windows. Gene-level candidate status was then assigned using the best, lowest mismatch count observed across any transcript associated with that gene. EFEMP1 was excluded from all off-target enrichment analyses because it represents the intended on-target transcript.

Three candidate sets were defined. Set A included genes with full 20-mer complementarity allowing ≤2 mismatches. Set B included genes with full 20-mer complementarity allowing ≤3 mismatches. Set C included genes with an exact match to the central 10-mer gapmer core, independent of flanking sequence context. Set A and Set B were considered the primary sequence-similarity candidate sets because they preserve near-complementarity across the full ASO target sequence. Set C was included as a lower-confidence sensitivity set because exact matching to a 10-mer sequence can occur more frequently by chance, especially when the flanking wing regions contain multiple mismatches. However, Set C was retained because the central DNA gap is the region expected to support RNase-H-mediated cleavage in a gapmer ASO.

To account for the 5-10-5 gapmer architecture of the EFEMP1 ASO, a position-weighted mismatch score was calculated for each candidate. The ASO was modeled as containing a 5-nucleotide 5′ wing, a 10-nucleotide central DNA gap, and a 5-nucleotide 3′ wing. The central DNA gap, corresponding to positions 6–15 using 1-indexed coordinates, is expected to support RNase-H-mediated cleavage of the target RNA, whereas the flanking wing regions primarily contribute to duplex stability and nuclease resistance. The position-weighted score was calculated as:

PW score = (gap mismatches × 3.0) + (wing mismatches × 1.0)

where gap positions were defined as positions 6–15 and wing positions as positions 1–5 and 16–20. The 3:1 weighting was used as a heuristic prioritization scheme to reflect the greater expected functional

importance of gap-region complementarity for RNase-H-mediated cleavage. Lower position-weighted scores were interpreted as higher-priority predicted off-target candidates.

DNA:RNA duplex free energy was estimated to rank predicted off-target candidates by the expected stability of ASO:RNA hybrid formation. This analysis was used as a secondary prioritization approach and not as definitive evidence of direct off-target cleavage. Two published nearest-neighbor parameter sets were used. Banerjee et al. 2020 was used as the primary DNA:RNA thermodynamic model because it was measured under lower-salt conditions closer to physiological ionic strength. Sugimoto et al. 1995 was used as a sensitivity analysis because it is a classic DNA:RNA hybrid thermodynamic reference set. Mismatch-associated free-energy penalties were incorporated from Watkins et al. 2011 where applicable. For mismatch classes or sequence contexts not represented in the available parameter set, approximate mismatch penalties were applied and interpreted cautiously.

The ASO was modeled as a DNA strand hybridized antiparallel to the RNA transcript sequence. Watson-Crick RNA:DNA base pairs were defined as A:T, U:A, G:C, and C:G. For each candidate site, predicted DNA:RNA duplex ΔG was calculated at 37°C. Relative ΔG was calculated as:

$$\Delta\Delta G = \Delta G_{off\text{-}target} - \Delta G_{on\text{-}target}$$

More negative ΔG values indicate more stable predicted DNA:RNA duplexes, whereas smaller $\Delta\Delta G$ values indicate off-target candidates closer in predicted binding stability to the intended EFEMP1 target site. Candidate genes were prioritized based on concordance across full-length mismatch count, position-weighted mismatch score, DNA:RNA ΔG, and observed RNA-seq downregulation.

To test whether predicted off-target candidates were enriched among downregulated genes, Fisher exact tests were performed using expressed genes as the background. The primary expressed-gene background was defined as genes with DESeq2 baseMean ≥ 10 and non-missing differential-expression statistics. Genes were classified as significantly downregulated if adjusted P value <0.05 and log2 fold change ≤ -0.58 in EFEMP1 ASO-treated HMRECs compared with ASO mismatch-treated controls.

Sensitivity analyses were performed using alternative expressed-gene thresholds and candidate definitions, including full 20-mer $\leqslant 2$ mismatches, full 20-mer $\leqslant 3$ mismatches, core-only exact matches, and combined candidate sets.
