# Supplementary material for: Development of an antisense oligonucleotide targeting EFEMP1 in models relevant to macular degeneration
Source: Mol Ther Nucleic Acids. 2026 Jul 21;37(3):103024. doi: 10.1016/j.omtn.2026.103024 (PMC13469845; doi:10.1016/j.omtn.2026.103024)
Supplement: Supplementary file 2 [file mmc4.pdf]

Molecular Therapy
Nucleic Acids
Original Article

Development of an antisense oligonucleotide targeting EFEMP1 in models relevant to macular degeneration

William P. Miller,[1,7] Said Arevalo-Alquichire,[1,7] Paula Perez-Corredor,[1,7] Rose Lin,[1] Anil Upreti,[1] Jeysson Sanchez-Suarez,[1] Tim E. Vanderleest,[1] Claudia Marino,[1,2] Donovan Spencer,[1] Matthew Scribner,[1] Jebgy Vargas,[1] Andres Muriel-Torres,[1] Xinyao Hu,[1] Yvonne Adu-Rutledge,[1] Bryan A. Kaplan,[1] Karim W. Barake,[1] Audrey L. Gunawan,[1] Aruvi Vijikumar,[1] Reeya Shrestha,[1] Anton Lennikov,[1] Leo A. Kim,[3] Joseph F. Arboleda-Velasquez,[1] Andrew Peterson,[4] Sudhir Agrawal,[4] Vinod Vathipadiekal,[5] Carlos Loya,[4] and Michael O'Hare[1,6]

[1]Schepens Eye Research Institute of Mass Eye and Ear and the Department of Ophthalmology at Harvard Medical School, Boston, MA 02114, USA; [2]Department of Neurology, Sealy Institute for Drug Discovery and Mitchell Center for Neurodegenerative Diseases, The University of Texas Medical Branch, Galveston, TX, USA; [3]Mass Eye and Ear and the Department of Ophthalmology at Harvard Medical School, Boston, MA 02114, USA; [4]Aldebaran Therapeutics, Waltham, MA, USA; [5]Alloy Therapeutics, Waltham, MA, USA; [6]Wellcome-Wolfson Institute for Experimental Medicine, Queen's University Belfast, Belfast, UK

Antisense oligonucleotides (ASOs) are RNA-targeting therapeutics with broad potential for genetically defined and complex ocular diseases. The eye is particularly well suited for ASO delivery because of its compartmentalized anatomy, accessibility, and capacity for sustained intraocular drug retention. Autosomal dominant drusen (ADD), an inherited retinal dystrophy characterized by early drusen formation and secondary choroidal neovascularization (CNV), is caused by the EFEMP1 R345W mutation. Mutant EFEMP1 accumulates within the retinal pigment epithelium (RPE) and extracellular matrix, contributing to disease pathology. EFEMP1 is also elevated in the serum and RPE/choroid of patients with age-related macular degeneration (AMD), although its functional role in AMD remains incompletely defined. Here, we developed a biallelic EFEMP1-targeted ASO that achieved potent knockdown and modulated disease-relevant pathways in iPSC-derived RPE, human microvascular retinal endothelial cells (HMRECs), *ex vivo* human choroidal explants, and *in vivo* mouse models. EFEMP1 protein levels were increased in AMD tissue, and ASO-mediated EFEMP1 knockdown reduced complement factor 3 (C3), suppressed HMREC proliferation and migration, and inhibited angiogenic sprouting in choroidal explants. These findings support EFEMP1 as a pathogenic regulator of retinal degeneration and vascular dysfunction and establish EFEMP1-directed ASOs as a promising therapeutic strategy for ADD and AMD.

INTRODUCTION

EFEMP1 (also known as Fibulin-3) is an extracellular matrix (ECM) protein expressed in the retinal pigment epithelium (RPE) and choroid that has been implicated in ECM remodeling.[1–5] EFEMP1 accumulation is a defining feature of autosomal dominant drusen (ADD), also known as Doyne honeycomb retinal dystrophy (DHRD) or Malattia Leventinese (ML), an inherited macular dystrophy characterized by the formation of numerous drusen.[6] ADD causes progressive, irreversible loss of central visual acuity due to geographic atrophy and choroidal neovascularization (CNV).[7] The disease is genetically defined by a non-conservative missense mutation, R345W, in EFEMP1. Efemp1 knockout has been reported to protect against the development of sub-RPE deposits without producing an associated retinal phenotype.[8]

Several EFEMP1 binding partners have been implicated in ocular disease, including complement factor H (CFH), a potent regulator of complement factor 3 (C3) activity.[2,9] The proteolytic complement cascade can be activated through the classical, alternative, or lectin pathways, all of which converge at C3.[10] Cleavage of C3 generates C3b, which is deposited on cell surfaces and ECM; C3 is also a constituent of drusen and contributes to drusen formation. Another EFEMP1 binding partner is TIMP3, a potent anti-angiogenic factor that competes with vascular endothelial growth factor (VEGF) for VEGFR2.[11] Interestingly, mutations in TIMP3 cause Sorsby fundus dystrophy (SFD), which shares drusen formation with ADD but is characterized by a more severe CNV response.[12]

Received 15 December 2025; accepted 17 July 2026;
https://doi.org/10.1016/j.omtn.2026.103024.

[7]These authors contributed equally

Correspondence: Carlos Loya, Aldebaran Therapeutics, Waltham, MA, USA.
E-mail: carlos@aldebarantx.com
Correspondence: Michael O'Hare, Wellcome-Wolfson Institute for Experimental Medicine, Queen's University Belfast, Belfast, UK.
E-mail: m.ohare@qub.ac.uk

© 2026 The Authors.
Published by Elsevier Inc. on behalf of The American Society of Gene and Cell Therapy.
This is an open access article under the CC BY license (http://creativecommons.org/licenses/by/4.0/).

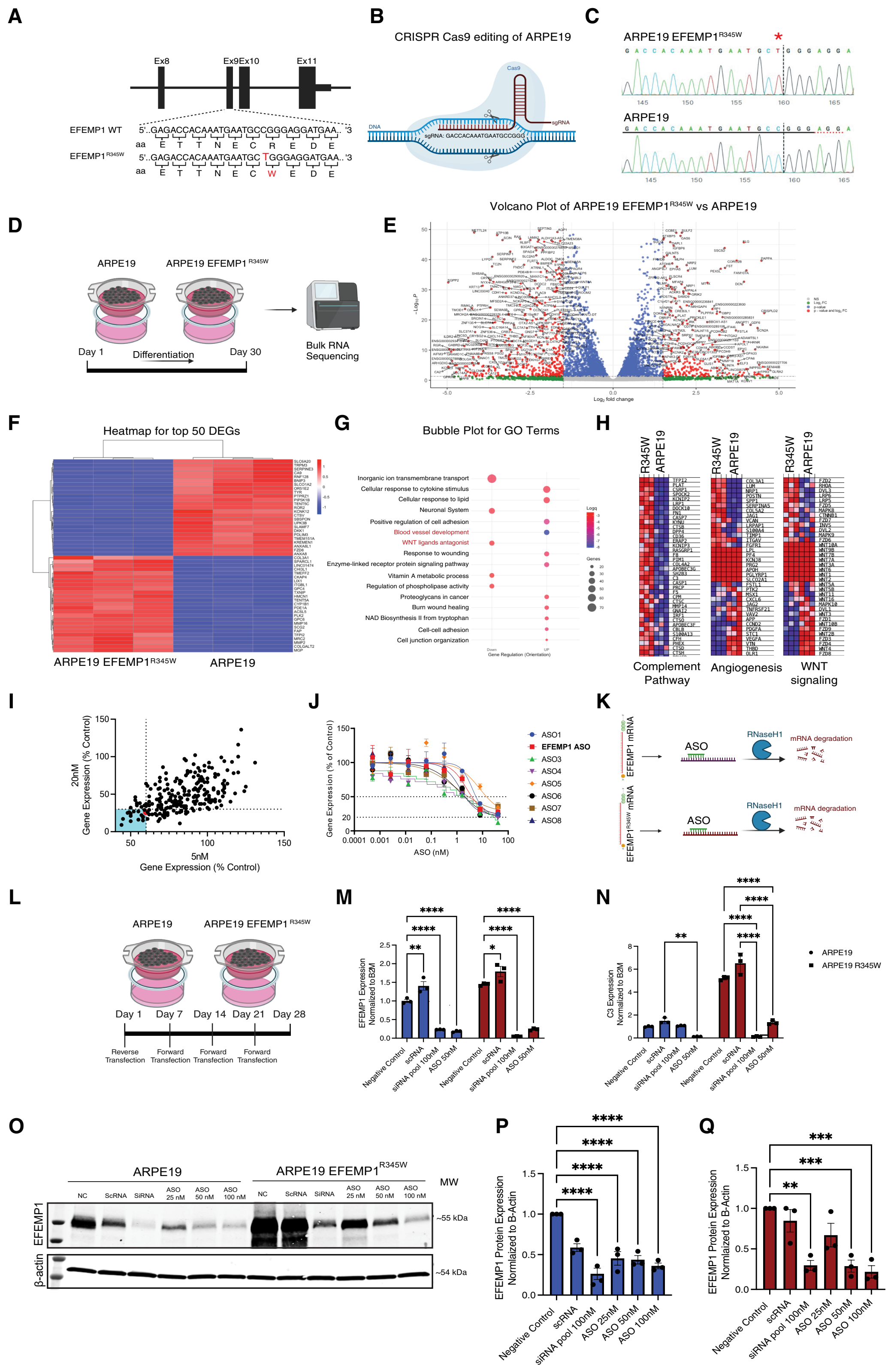

(legend on next page)

Although ADD often progresses more rapidly than age-related macular degeneration (AMD), the two diseases share several clinical and pathological features.[13] AMD is a debilitating progressive eye disease, clinically characterized by drusen formation, geographic atrophy, and CNV, that affects more than 19.8 million people in the United States. Geographic atrophy often referred to as late-stage dry AMD, is characterized by progressive neurodegeneration and accumulation of extracellular drusen and basal deposits. These lipid-containing aggregates can impair RPE and photoreceptor health by disrupting nutrient transport, promoting inflammation, and increasing the risk of CNV.[14–18] Despite recent FDA approvals of complement pathway inhibitors (Pegcetacoplan; Apellis Pharmaceuticals[19]/Avacincaptad pegol; Astellas Pharma[20]) for geographic atrophy, there still remains an urgent need for additional therapeutic options. In addition, complement inhibition is suspected of increasing the risk of retinal vasculitis.[21]

An emerging therapeutic strategy involves the use of antisense oligonucleotides (ASOs), which are versatile RNA-binding molecules that can effectively suppress mRNA translation, select for splice variants, or increase expression of preferred RNA transcripts.[22] Over 25 years ago, Fomivirsen (branded as Vitravene; Novartis, Basel Switzerland), a first-generation ASO, was approved for weekly intravitreal injection for cytomegalovirus (CMV) retinitis, and was later withdrawn from market.[23] However, the therapeutic potential of ASOs for ocular diseases is far from being fully realized. Several major breakthroughs in ASO chemistry have resulted in the development of new generations of gapmer, or, that are resistant to RNAse H-mediated decay increasing their durability.[24] To date, 13 ASO formulations have been approved by the Food and Drug Administration (FDA) for a variety of indications.[25]

EFEMP1 has been shown to be one of the top ten genes overexpressed in the RPE/choroid during aging.[26] Previous studies have demonstrated that EFEMP1 is upregulated in the RPE/choroid in dry AMD and in the serum, RPE, and choroid of patients with wet AMD.[27] Here, we evaluated the role of EFEMP1 in the development of pathologies associated with ADD and AMD, and assessed EFEMP1 targeting ASOs as a potential therapeutic strategy.

RESULTS

CRISPR-Cas9 edited ARPE19 cells express EFEMP1^{R345W}

EFEMP1^{R345W} expressing ARPE19 cells were generated using CRISPR-Cas9 editing of exon 9 (Figure 1A). A single guide RNA sequence (sgRNA-seq) was used to direct CRISPR-Cas9 editing (Figure 1B), and the expected sequence alteration was confirmed by sequencing (Figure 1C). We then differentiated edited ARPE19 EFEMP1^{R345W} for 30 days to compare the transcriptomic profile with ARPE19 cells (Figure 1D). We carried out bulk RNA sequencing (RNA-seq) on ARPE19 and ARPE19 EFEMP1^{R345W} (or ARPE19 R345W) cells and show differentially expressed genes (DEGs) in a volcano plot (Figure 1E; bulk RNA-seq data are available in GEO:GSE289976). A heatmap of the top 50 DEGs including (upregulation of genes associated with ECM remodeling including *COL3A1*, *MMP2*, *MMP16*, and *COLGALT2*) (Figure 1F). Gene Ontology (GO) analysis for downregulated genes in ARPE19 R345W reveals enrichment in inorganic ion transmembrane transport, neuronal system, WNT ligand antagonists, vitamin A metabolic process, and regulation of phospholipase activity (full list of GO terms available in Table S1) while the upregulated genes associated with cellular response to cytokine stimulus, cellular response to lipid, positive regulation of cell adhesion, blood vessel development, response to wounding, enzyme-linked receptor protein signaling pathway, proteoglycans in cancer, burn wound healing, NAD biosynthesis II from tryptophan, cell-cell adhesion, and cell junction organization (Figure 1G). Gene set enrichment analysis (GSEA) identified complement pathway, angiogenesis and WNT signaling as some of the terms found by gene enrichment analysis. (Figure 1H). These data are consistent with the reported complement phenotype previously observed in ARPE19 R345W cells, indicating that we have developed a reliable model to screen drug candidates to reduce EFEMP1 expression. To identify a potent EFEMP1-targeted ASO, we first screened 247 drug-like candidates and identified EFEMP1-directed ASO candidates that efficiently reduce the mRNA expression of EFEMP1 *in vitro* by more than 50% relative to control (Figure 1I). The top 8 candidates were evaluated in an eight-concentration dose-response assay, leading to the selection of a lead candidate (EFEMP1 ASO; IC50 of 3.807 nM) that was taken forward for further investigations (Figure 1J). We then used the ARPE19 and ARPE19 R345W cells to assess ASO-mediated reduction of both EFEMP1 and EFEMP1^{R345W} expression (Figure 1K). We aimed

Figure 1. Development of an ARPE19 EFEMP1^{R345W} mutant cell line for screening of EFEMP1 targeting ASOs

(A) Schematic of comparison of EFEMP1 and EFEMP1^{R345W} nucleotide sequence and associated amino acid change from arginine to tryptophan. (B) Schematic showing sgRNA-seq for CRISPR-Cas9 editing of ARPE19 cells. (C) Sanger sequencing of ARPE19 and ARPE19 R345W confirming successful nucleotide replacement. (D) Schematic showing ARPE19 and ARPE19 R345W cell differentiation across 30 days prior to bulk RNA sequencing. (E and F) Volcano plot showing significant gene changes in ARPE19 R345W vs. ARPE19 cells ($N = 3$ per group). (F) Heatmap of the top 50 DEGs from ARPE19 R345W and ARPE19 cells. (G) Bubble plot for Gene Ontology (GO) terms enriched in ARPE19 R345W cells. (H) Heatmap for complement, angiogenesis, and WNT signaling pathways identified by GSEA. (I) Graph showing two-dose screen (5 and 20 nM, in triplicate) of 247 ASOs directed at hEFEMP1 compared to untreated cell control. (J) Graphs showing dose-response (0.5 pM–40 nM) of top candidate ASOs. (K) Schematic of biallelic antisense oligonucleotides that target the EFEMP1 and EFEMP1^{R345W}. (L) Schematic of transfection protocol of small interfering RNA (siRNA) pool and EFEMP1 targeted ASO in ARPE19 and ARPE19 R345W cells. (M and N) qPCR data of EFEMP1 mRNA expression after treatment with siRNA pool (100 nM), scRNA (100 nM), ASO (50 nM) in ARPE19 or ARPE19 R345W cells ($N = 3$ per group). (N) qPCR data of C3 mRNA expression after treatment with siRNA pool (100 nM), scRNA (100 nM), ASO (50 nM) in ARPE19 or ARPE19 R345W cells ($N = 3$ per group). (O) Western blotting showing ARPE19 EFEMP1^{R345W} cell line has increased expression of EFEMP1 relative to untreated wild type control. (P and Q) EFEMP1 targeting siRNA pool 100 nM, EFEMP1 directed ASOs 50 and 100 nM, substantially knockdown EFEMP1 ($N = 3$, biological replicates). Data were analyzed using a one-way ANOVA with Tukey's post hoc test for multiple comparisons $^{*}p < 0.05$; $^{**}p < 0.01$; $^{***}p < 0.001$; $^{****}p < 0.0001$.

to identify an ASO capable of reducing either EFEMP1 or EFEMP1 R345W expression *in vitro* by reverse transfection on day 1 and forward transfection up to 21 days (Figure 1L). We determined 50 nM of EFEMP1 ASO significantly reduced EFEMP1 and EFEMP1^{R345W} mRNA expression levels in ARPE19 and ARPE19 R345W cells, respectively (Figure 1M). While ARPE19 R345W cells demonstrated significantly (5×) increased levels of complement factor 3 (C3) compared to ARPE19 cells. We report that ASO knockdown of EFEMP1 significantly reduced C3 expression in both ARPE19 and ARPE19 R345W cells (Figure 1N). We further confirmed the reduction of EFEMP1 at the protein level after treatment with either scramble (sc)RNA (100 nM), a short interference (si)RNA pool (100 nM) that is comprised of four independent EFEMP1 targeting sequences or different concentrations of an EFEMP1 ASO (25, 50, and 100 nM) in ARPE19 or ARPE19 R345W cells (Figure 1O). The quantitative analysis by densitometry demonstrated significant reduction in EFEMP1 protein expression with EFEMP1 ASO 25, 50, and 100 nM (Figure 1P) and EFEMP1 R345W (50 and 100 nM) (Figure 1Q) in the ARPE-19 cultures. To assess ECM changes in ARPE19 R345W cells, we carried out mass spectrometry after decellularization of transwells after a month of culture of ARPE19 and ARPE19 R345W cells (Figure S1A). We report a 14.2-fold increase in EFEMP1 protein levels in ARPE19 R345W ECM (Figure S1B). Ingenuity pathway analysis (IPA) was used to highlight predicted networks of interest and indicates increased protein kinase b (AKT), phosphoinositide 3-kinase (PI3K) pathway activation as well as reduced APP-related pathways (Figures S1C–S1E). High temperature requirement protein A1 (HTRA1) is upregulated (5.95-fold) in the ECM of ARPE19 R345W cells. HTRA1 variants are highly significant genetic risk factors for AMD. The upregulation of HTRA1 in the ECM of ARPE19 R345W cells is striking (Figures S1B, S2A, and S2B). We confirmed protein level increases of HTRA1 using western blotting of whole cell lysates of ARPE19 R345W cells (Figure S2C).

Lentiviral overexpression of EFEMP1 and EFEMP1^{R345W} is inhibited by EFEMP1-targeted ASOs in iPSC-RPE cells

We tested a lentiviral CMV promoter-driven construct encoding either enhanced green fluorescent protein (EGFP) alone or EFEMP1 and EFEMP1^{R345W} that were conjugated to EGFP by a T2A linker (Figure 2A). We report robust EGFP expression after transduction of the CMV_EGFP construct at multiplicity of infection (MOI): 5, 10, or 15 in induced pluripotent stem cells (iPSC)-derived RPE cells. We report that CMV_EFEMP1 and CMV_EFEMP1 ^{R345W} (MOI:10) showed the strongest EGFP expression. Therefore, all future experiments were conducted under those parameters. qPCR revealed significant upregulation of EFEMP1 levels after treatment with CMV_EFEMP1 or CMV_EFEMP1^{R345W} in iPSC-RPE cells (Figure 2B). We confirmed that the EFEMP1 ASO (100 nM) could significantly reduce EFEMP1 mRNA levels compared to the vehicle control or an ASO mismatch in iPSC-RPE cells (Figure S3). Immunofluorescence staining and confocal imaging of iPSC-RPE reveals robust overexpression of EFEMP1 and EFEMP1 R345W after lentiviral transduction compared to the negative control cells using an anti-EFEMP1 antibody (magenta) in human iPSC-derived RPE cells (Figure 2C). The successful reduction of EFEMP1- and EFEMP1 R345W-induced overexpression via an EFEMP1-directed ASO (100 nM) was confirmed by qPCR and fluorescence quantification using an automated method (Figures 2D and 2E; Figure S4). We report significant increases in C3 expression after EFEMP1 or EFEMP1 R345W overexpression. The EFEMP1 ASO significantly reduced C3 levels in both groups (Figures S5A and S5B).

EFEMP1 knockdown inhibits angiogenic properties of HMRECs

The transcriptomics analyses performed on RPE cell cultures identified genes from several pathways relevant to angiogenesis that were differentially regulated in response to the presence of the EFEMP1 R345W mutation (see Figure 1H). Thus, as a subsequent investigation within our study, we evaluated the impact of EFEMP1 knockdown on endothelial cells to determine whether targeting EFEMP1 held potential antiangiogenic effects. To assess this, primary cultures of human microvascular retinal endothelial cells (HMRECS) were transfected with either our EFEMP1 ASO (EFEMP1 ASO), or a version of the EFEMP1 ASO that contains nucleotide substitutions in the core sequence to disrupt interaction with the target EFEMP1 sequence (ASO mismatch, Figure S3), or negative control. Relative to the ASO mismatch, 50 nM of EFEMP1 ASO was sufficient to significantly reduce EFEMP1 expression in HMRECs at the protein level by ∼60%, and at the mRNA level by ∼80% with no cytotoxicity as measured by LDH secretion. (Figures 3A–3D). After determining that the EFEMP1 ASO could be applied to HMRECs in culture, bulk transcriptomics analysis was performed on RNA isolated from non-treated cells (NTCs), cells transfected with ASO mismatch and cells transfected with EFEMP1 ASO 48 h after treatment (data available in GEO: GSE289976). Analysis of the transcriptomics data revealed several differentially regulated genes between the EFEMP1 ASO, and the control groups. Several prominent genes are downregulated after treatment with EFEMP1 ASO including PSAP, EFEMP1, MMP2, and CFH, while gene including HMOX1, FTH1, and CDKN1A were upregulated (Figures 3E and 3F). Further analysis to establish GO terms identified multiple pathways relevant to angiogenesis and other related pathologies (Figure 3G; Table S2). GSEA identified vasculature development as one of the enriched terms. We took the genes listed in the GSEA term vasculature development and display the identified DEGs in a heatmap (Figure 3H). To complement our GO analysis, we performed GSEA to identify other relevant factors associated with the bulk RNA-seq data. This led to the identification of the gene EPAS1, commonly known as hypoxia-inducible factor 2α (HIF2α), which is more selectively expressed in endothelial cells relative to the ubiquitously expressed HIF1α variant. HIF2α, which is associated with vascular development, was significantly downregulated after treatment with the EFEMP1 ASO. Other relevant genes included IFT122, MAP3K3, CDH13, SOX18, CDKN1A, and EXT1 (Figure 3I). We then preformed functional studies to determine how EFEMP1 knockdown was influencing endothelial cell behavior. A scratch wound assay was performed 48 h post-transfection to assess the impact of the EFEMP1 ASO on endothelial

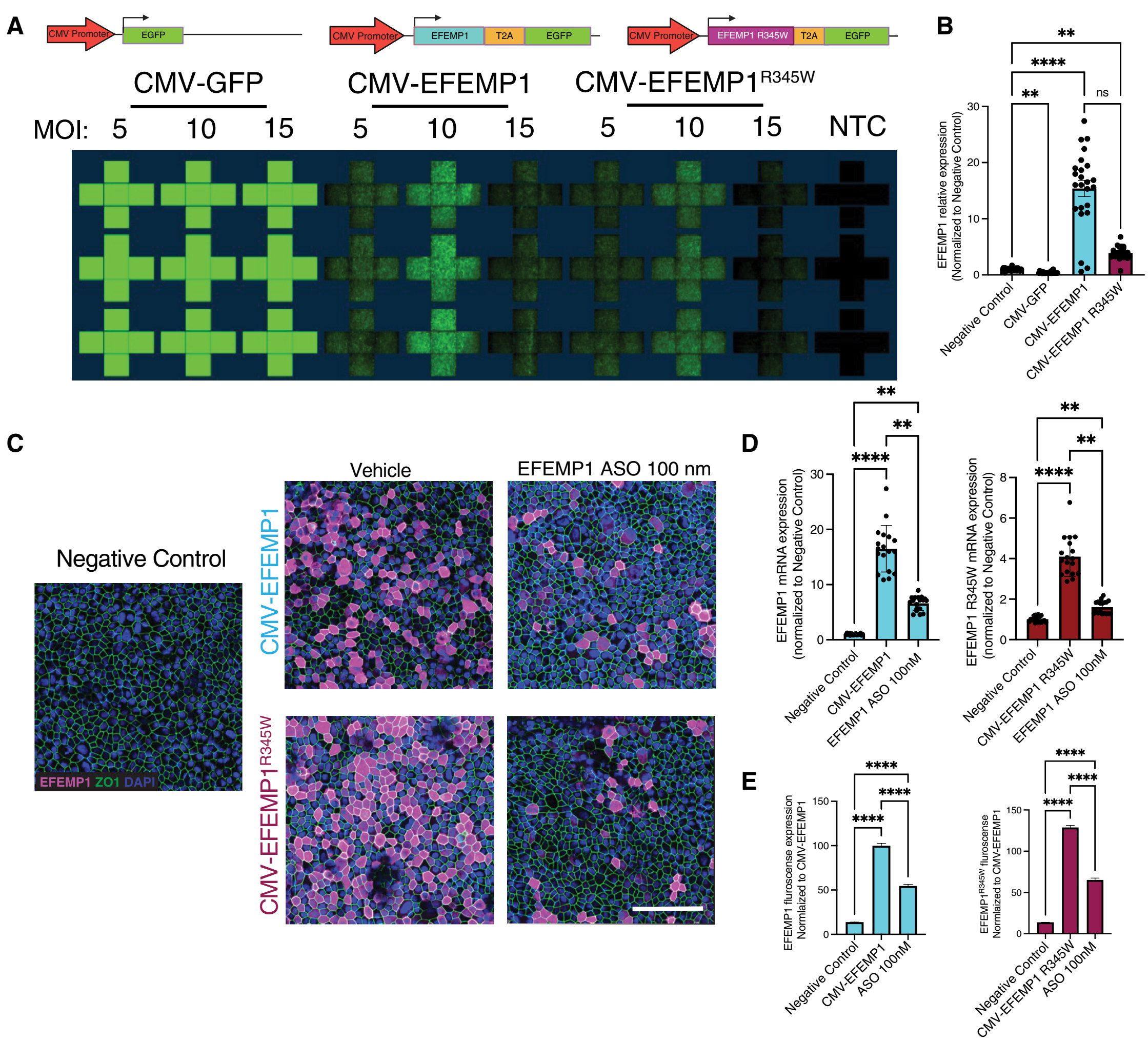

Figure 2. Lentiviral overexpression of EFEMP1 and EFEMP1 R345W in iPSC-RPE can be efficiently knocked down using a EFEMP1-targeted ASO

(A) Comparison of lentiviral transduction efficiency of constructs expressing either EGFP, EFEMP1 with a T2A linkage to EGFP or EFEMP1^{R345W} with a T2A linkage. (B and C) qPCR of EFEMP1 expression in iPSC-RPE. (C) Representative confocal images in iPSC-RPE showing Lentiviral overexpression of either wild type EFEMP1 and EFEMP1R345W (n = 4, biological replicates). DAPI, blue nuclear stain; ZO-1, tight junction protein marked in green; EFEMP1, visualized in magenta (×40, scale bars, 100 μm). (D) qPCR showing mRNA expression of EFEMP1 levels after EFEMP1 overexpression and effective of EFEMP1 targeted ASOs (N = 3, biological replicates). (E) EFEMP1 intensity quantified relative to total cells in field via watershed algorithm and automated fluorescence quantification in iPSC-RPE cells overexpressing EFEMP1 comparing EFEMP1 expression after treatment ASO 100 nM or a siRNA pool 100 nM. Data were analyzed using a one-way ANOVA with Tukey's post hoc test for multiple comparisons.

**$p < 0.01$; ****$p < 0.0001$.

cell migration. After 18 h, HMRECS transfected with the EFEMP1 ASO exhibited a significant reduction in migratory capacity relative to cells transfected with the ASO mismatch (Figure 3J). As an additional measure of angiogenic potential, EFEMP1 knockdown was concomitant with a significant decrease in cellular proliferation 24, 48, and 72 h after transfection (Figure 3K; Figure S6). HIF1α expression was evaluated 48 h post-transfection by western blotting and relative to the ASO mismatch, a significant reduction in HIF1α expression was observed at the protein level of ~80% (Figure 3L). We utilized *in silico* sequence-complementarity analysis using the EFEMP1 ASO sequence. Candidate off-target genes were explored using three complementary approaches: full 20-mer mismatch scanning, central gapmer-core matching, and DNA:RNA duplex free-energy ranking. EFEMP1 was excluded from off-target enrichment statistics because it represents the intended on-target transcript. The highest-priority off target candidates were RHBDF1 and VAC14, both of which contained two mismatches to the ASO target sequence, showed predicted DNA:RNA duplex stability, and were downregulated in the RNA-seq dataset. These computational predictions identify candidate sequence-dependent off-targets but do not prove direct RNase-H-mediated cleavage (Figures S7A and S7B).

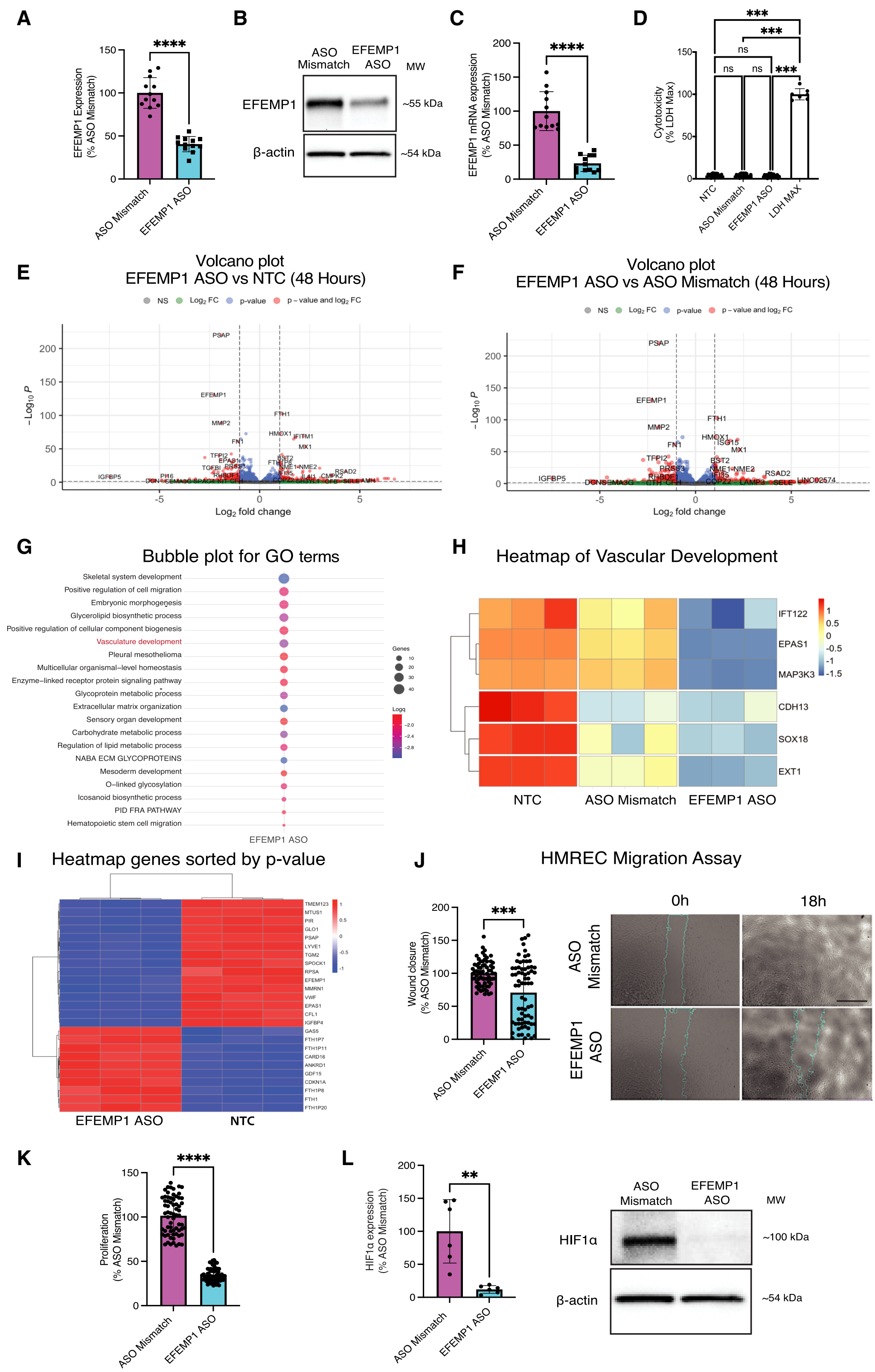

(legend on next page)

EFEMP1 knockdown inhibits growth in *ex vivo* choroidal explants derived from mouse and human tissue

To enhance the translational value of our findings, we first performed intravitreal injections of the EFEMP1 ASO in age-matched, 12-week-old C57BL/6J mice at doses including 1, 10, and 50 μg to validate the EFEMP1 knockdown *in vivo*. After 72 h, the eyes were enucleated, separating the anterior and posterior segments (Figure S8A). Posterior segments were then homogenized, and EFEMP1 mRNA levels were quantified at each of the doses tested. While the 1 μg dose showed no reduction in EFEMP1 mRNA expression relative to the vehicle control, significant decreases in EFEMP1 levels were obtained at the 10 and 50 μg doses. We then evaluated the duration of the knockdown effect. Mice injected with 15 μg of EFEMP1 ASO maintained significant EFEMP1 knockdown up to 30 days post-injection relative to vehicle controls (Figure S8B). We have shown strong EFEMP1 expression in retinal muller cells from adult C57BL/6 mice (Figure S9A). Retinal structure is preserved with no reactive gliosis (glial fibrillary acidic protein [GFAP] staining) in EFEMP1 knockout animals. GFAP expression is generally restricted to retinal astrocytes and nearly absent in Müller cells. Following retinal damage, disease, or stress, Müller cells become rapidly activated (reactive gliosis) and dramatically upregulate GFAP expression. These data suggest that EFEMP1 knockout is unlikely to have a drastic effect on muller cell health and overall retina structure (Figure S9B).

To test the antiangiogenic potential of the EFEMP1 ASO in mouse eyes, we next used mouse choroidal explants as a controlled *ex vivo* system to determine whether EFEMP1 knockdown suppresses angiogenic outgrowth in intact ocular tissue.[28] In this assay, the RPE/choroid complex is removed from the eye and cultured outside the animal while preserving local interactions among resident choroidal endothelial cells, stromal cells, ECM, and neighboring ocular tissue components that are not captured in monoculture. Sprouting from the edge of the explant therefore provides a tissue-level readout of angiogenic outgrowth in a reproducible experimental system. Mouse eyes were enucleated, the RPE/choroid was isolated, and 1 mm punches were generated from the tissue before embedding in a matrix containing basement membrane extract (Figure 4A). Choroidal explants were transfected with 50 nM of ASO mismatch or EFEMP1 ASO and bright field images were taken every other day over 4 days. The quantification of the total growth area indicated a significant reduction in the growth of EFEMP1 ASO-transfected choroids relative to controls (Figures 4B and 4C). Calcein imaging was also performed on the final day to allow for growth quantification in 3 dimensions while also demonstrating sprout viability (Figure 4D). These data indicated that sprouts consisted of live-cells and confirmed the inhibition in growth caused by the EFEMP1 assay. We then extended this approach to postmortem human choroidal explants as a complementary translational model. In this system, RPE/choroid tissue is isolated from donor eyes after death, embedded in basement membrane extract, and cultured to allow resident human choroidal cells to migrate and sprout into the surrounding matrix. Although the tissue is no longer connected to systemic circulation, this model preserves human ECM, resident choroidal cell populations, and donor-specific tissue features, while still allowing controlled *ex vivo* treatment with ASO mismatch or EFEMP1 ASO. (Figure 4E). Under the same conditions, explant outgrowth transfected with EFEMP1 ASO 50 nM was also significantly reduced in human tissue as determined by quantification using brightfield microscopy (Figures 4F and 4G). Successful transfection and knockdown of EFEMP1 in the human choroidal explants was also confirmed by western blotting (Figure 4H). Calcein imaging further substantiated a significant reduction in outgrowth of EFEMP1 ASO-transfected choroids relative to ASO mismatch controls (Figure 4I).

EFEMP1 is upregulated in AMD and EFEMP1 knockdown reduces AMD choroidal explant growth

To assess the levels of EFEMP1 and its binding partner TIMP3 in AMD, we dissected the RPE/choroid from enucleated eyes obtained postmortem from patient donors and compared expression in AMD and non-AMD derived tissue (for demographics, see Table S3). This provided a disease-relevant extension of the human explant model and enabled assessment of whether EFEMP1 knockdown suppresses outgrowth in both non-AMD and AMD RPE/choroid tissue. Immunofluorescence staining revealed clear increases in EFEMP1 protein staining (green) in the RPE of the AMD sample compared to the non-AMD (healthy control) eye. TIMP3 (magenta) staining is in close proximity to staining for EFEMP1 and is associated with cell junctions in the AMD eyes (Figure S10A). Western blotting of the RPE/choroid confirms the upregulation of EFEMP1 in AMD (Figure S10B). Bright field images show reduced growth of non-AMD and AMD choroidal explants at 0, 6, and 12 days after EFEMP1 ASO treatment (Figures 5A and 5B). We determined that

Figure 3. EFEMP1-directed ASOs reduced endothelial proliferation, migration and pathways relevant to vascular development

(A and B) Western blotting of ASO mismatch (50 nM) vs. EFEMP1 ASO (50 nM) in human microvascular endothelial cells (HMRECs) ($N = 3$, biological replicates). (C and D) qPCR of EFEMP1 knockdown comparing ASO mismatch (50 nM) vs. EFEMP1 ASO (50 nM) in HMRECs. (D) LDH assay comparing ASO mismatch and EFEMP1 ASO (50 nM) compared to LDH max (one-way ANOVA; $p < 0.001$). (E) Volcano plot of bulk RNA-seq comparing EFEMP1 ASO and NTC in HMRECs ($n = 3$ per group). (F) Volcano plot of bulk RNA-seq comparing EFEMP1 ASO and ASO mismatch ($n = 3$ per group). (G) Bubble plot for Gene Ontology (GO) terms enriched in EFEMP1 ASO endothelial cells. (H) Z score heatmap displays vascular development genes from EFEMP1 ASO, ASO mismatch and NTC in HMRECs. (I) Heatmap of the top DEGs from EFEMP1 ASO and NTC in HMRECs. (J and K) Bar graph of migration assay comparing ASO mismatch and EFEMP1 ASO (50 nM) and representative bright field images at 0 and 18 h ($N = 3$ biological replicates, $n = 50$, technical replicates, scale bars, 750 μm). (K) cyQUANT proliferation assay shows significant reduction in proliferation after treatment with EFEMP1 ASO (50 nM). (L) Bar graph of HIF1α protein expression and representative western blot ($n = 6$). Data were analyzed by Mann-Whitney U test or Kruskal-Wallis with Dunn's post hoc test for multiple comparisons.

**$p < 0.01$; ***$p < 0.001$; ****$p < 0.0001$.

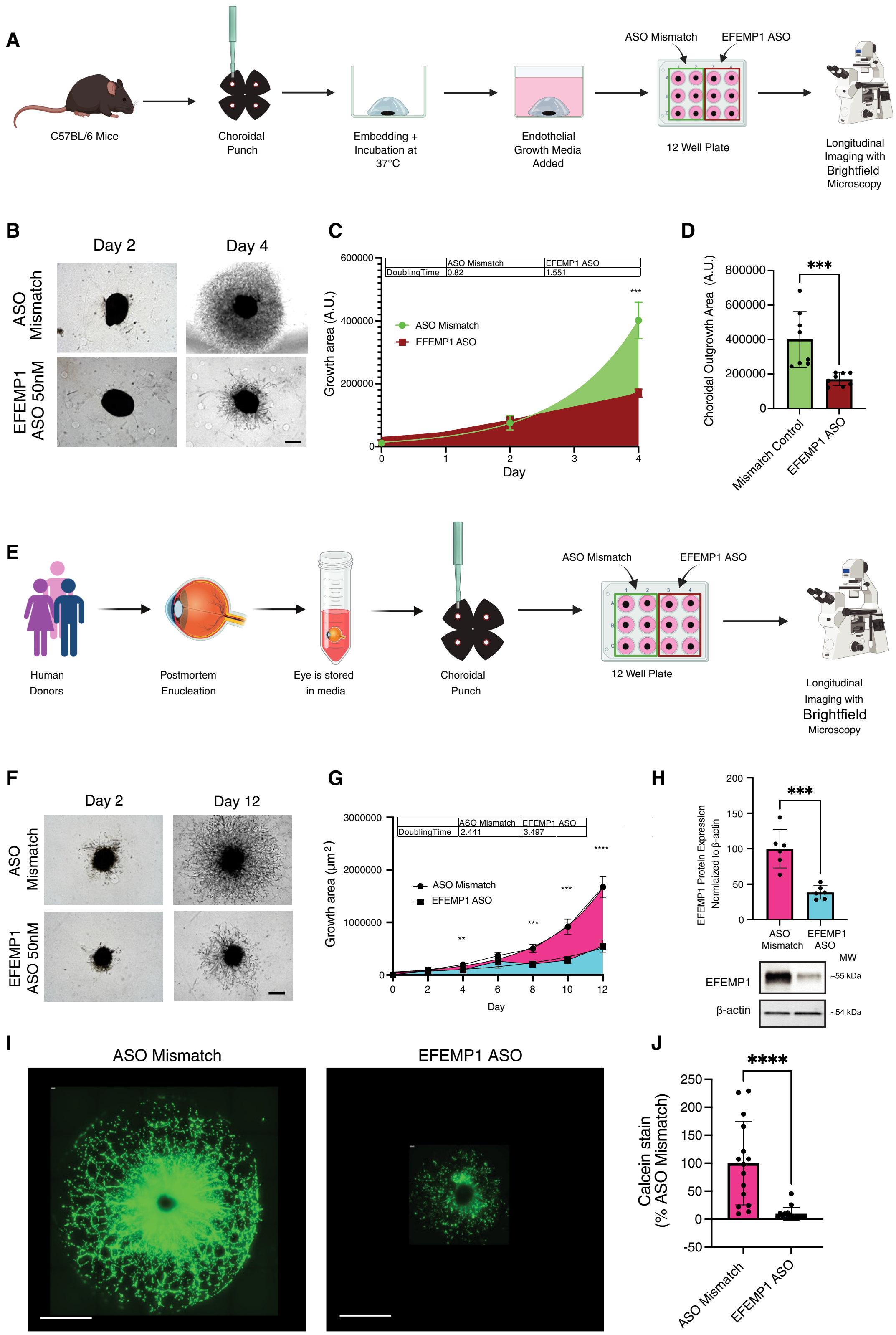

(legend on next page)

treatment with 50 nM EFEMP1 ASO significantly reduced the growth rates of AMD choroidal explants compared to AMD explants treated with ASO mismatch (Figure 5C). We compared area under the curve (Figure 5D) We assessed non-AMD and AMD choroidal explants using Calcein imaging and confirmed a significant reduction in outgrowth after EFEMP1 ASO treatment relative to ASO mismatch controls (Figures 5E and 5F).

DISCUSSION

The potential of EFEMP1-targeted ASOs therapy in the treatment of ADD and AMD represents an exciting and novel paradigm against these debilitating ocular conditions. ADD and wet AMD share abnormal CNV as a common pathological phenotype and subsequent retinal damage, which is primarily treated with anti-VEGF therapies, which aim to inhibit pathological blood vessel growth.[27] However, while anti-VEGF agents are effective, they often require frequent injections and may not fully address the underlying molecular mechanisms driving disease progression.[29] This has led to increasing interest in exploring alternative therapeutic strategies that target other key players in the pathogenesis of ADD and wet AMD, such as EFEMP1.

Furthermore, as a secreted ECM protein, EFEMP1 plays a critical role in the regulation of ECM remodeling, complement factor expression, and angiogenesis.[27] These processes are impacted during both ADD and AMD progression. We propose that targeting EFEMP1 may have several advantages over the current FDA-approved therapies such as pegcetacoplan and avacincapted pegol that target either C3 or C5 alone.[30,31] Previous literature reports indicated that EFEMP1 modulation impacts several complement factors implicated in AMD pathophysiology, including C3, CFH, and CFB.[2,32] We found highly significant upregulation of C3 in EFEMP1^{R345W} cells and downregulation of C3 in response to EFEMP1 knockdown. Furthermore, EFEMP1-targeted ASOs reduced CFH expression in HMRECs after 48 h. C3 expression, activation, and subsequent complement pathway activation are strongly associated with drusen formation and disease progression in AMD.[33] C3-convertase (C3bBb) is formed through complement factor B and factor D activity. This drives an amplification loop by cleaving more C3 into C3b. Importantly, this cascade can be halted by CFH-mediated recruitment of complement factor I, which cleaves C3b and inactivates it. EFEMP1 R345W mice develop sub-RPE deposits reminiscent of drusen after and introducing C3 or CFB knockout alleles into these mice prevents sub-RPE deposit formation,[3] indicating a pathological link between EFEMP1, complement activity, sub-RPE deposition development, and disease progression.

Interestingly, high-temperature requirement protein A1 (HTRA1) accumulates in the ECM of ARPE19 R345W cells by over 20-fold compared to ARPE19 wild-type (WT) cells. Variants of HTRA1 are associated with AMD risk, and alternations in HTRA1 function have been reported in other macular dystrophy's including late onset retinal degeneration (L-ORD).[34] HTRA1 was recently found to cleave EFEMP1; however, the implications of cleavage have not been explored. It was further reported that HTRA1 cleaves both EFEMP1 and EFEMP1 R345W with similar efficiency in cell-free systems; however, in the context of ADD EFEMP1 R345W accumulations are robust and may require greater levels of serine protease activity to break down EFEMP1 that has accumulated beneath the RPE.[35]

EFEMP1 modulation also impacts other factors expression including ECM remodeling factors such as MMP2 and COL3A1 that have recently received attention in the context of drusen formation for Sorsby's fundus dystrophy and AMD.[36] We show clear evidence that MMP2 is upregulated in ARPE19 R345W cells consistent with previous reports[2]; however, we now provide evidence that EFEMP1 knockdown reduces MMP2 expression significantly in HMRECs. We propose that EFEMP1 regulates MMP2 expression through altered Notch signaling levels as EFEMP1 contains a Delta, Serrate, Lagg (DSL) domain and Notch signaling is a known downstream effector of EFEMP1.[37] Supporting the relationship between Notch signaling and EFEMP1, GSEA revealed Notch signaling as an enriched pathway in ARPE19 R345W with an upregulation of Notch2, DLL1, and JAG1. Furthermore, HMRECs treated with the EFEMP1 ASO had altered Notch signaling with a downregulation in genes, including JAG1 and HES1.

In the context of angiogenesis, EFEMP1 knockdown drastically reduces endothelial cell proliferation and migration in HMRECs and choroidal explants from both mouse and human eyes. GO analysis highlighted vascular development to be downregulated after EFEMP1 ASO treatment at 48 h. We found that a major gene downregulated within the vascular development pathways is EPAS1 (HIF2α), which has previously been found to be upregulated in correlation with EFEMP1 upregulation. Further, EFEMP1

Figure 4. EFEMP1 knockdown reduces choroidal sprouting in both mouse and human tissue

(A) Schematic of experimental design for mouse choroidal explant isolation, embedding, treatment and imaging. (B) Bright field images of mouse choroidal explants at day 2 and day 4 after transfection with ASO mismatch control or EFEMP1-targeted ASO (50 nM). (C) Chart showing the growth rate of mouse choroidal explants across time ($N = 3$, biological replicates, $n = 8$, technical replicates). (D–F) Bar graph of mouse choroidal outgrowth area treated with ASO mismatch or EFEMP1 ASO (50 nM; $n = 8$). (E) Schematic of human choroidal explant isolation, embedding, treatment and imaging. (F) Bright field images of human choroidal explants at day 2 and day 12 after transfection with ASO mismatch control or EFEMP1-targeted ASO (50 nM). (G and H) Chart showing the growth rate of human choroidal explants across time ($N = 3$, biological replicates, $n = 12$, technical replicates) (H) Representative western blot of EFEMP1 expression compared to β-actin. (I) Representative immunofluorescence image of calcein imaging in human choroidal explants (images were rescaled and framed on a black background). (J) Bar graph of calcein staining quantification comparing ASO mismatch control or EFEMP1 targeted ASO (50 nM) ($N = 3$, biological replicates, $n = 12$, technical replicates). Data were analyzed by multiple paired t tests or by Mann-Whitney U test. ***$p < 0.001$; ****$p < 0.0001$.

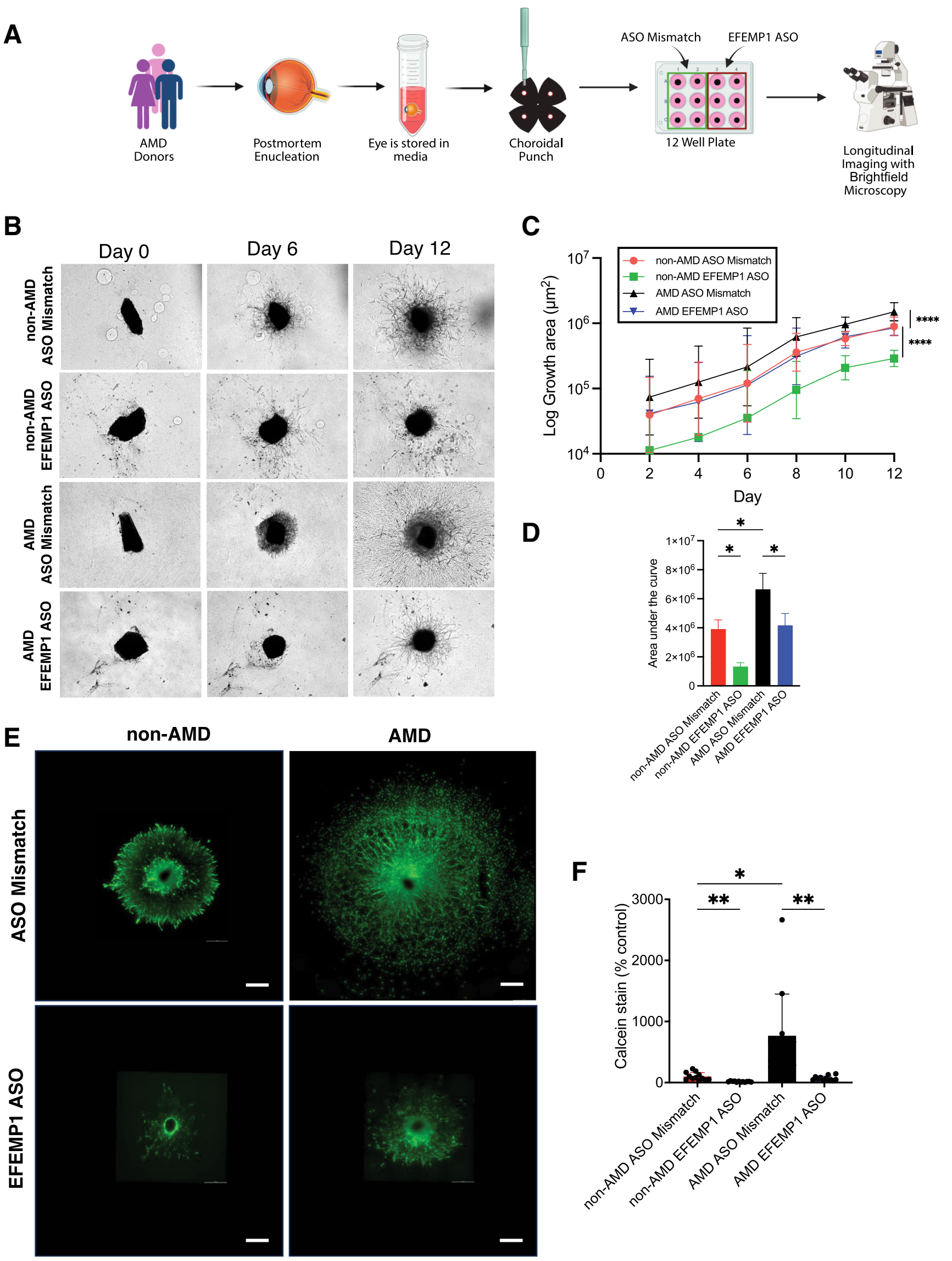

Figure 5. EFEMP1 ASOs reduce AMD choroidal explant growth rates

(A) Schematic of experimental design for AMD donor choroidal explant isolation, embedding, treatment and imaging. (B) Bright field images comparing non-AMD and AMD choroidal explants at 0, 6, and 12 days between ASO mismatch and EFEMP1 ASO (50 nM), scale bars, 200 μm. (C and D) Growth rates of non-AMD and AMD choroidal explants with ASO mismatch and EFEMP1 ASO (50 nM). (D) Bar graph comparing area under the curve from non-AMD and AMD choroidal explants with ASO mismatch and EFEMP1 ASO (50 nM). (E) Calcein immunoflurescence comparing ASO mismatch and EFEMP1 ASO (50 nM), (images were rescaled and framed on a black background), (scale bars, 1 mm). (F) Bar graph comparing calcein staining between ASO mismatch and EFEMP1 ASO (50 nM), ($N = 3$ biological; $n = 12$ technical). Data were analyzed by multiple paired t tests or by Mann-Whitney U test. $^{**}p < 0.01$; $^{***}p < 0.001$; $^{****}p < 0.0001$.

was suggested to be a downstream effector of HIF2α.[38] Our data suggest that EFEMP1 downregulation may be directly affecting HIF2α expression or alternatively via a feedback loop mechanism. Alterations in other genes associated with hypoxia after EFEMP1 ASO treatment was found by GSEA. Given the robust reduction in proliferation and migration, we also assessed the levels of HIF1α, a variant that is more ubiquitously expressed than HIF2α. We found that indeed HIF1α protein levels were significantly reduced at 48 h. Given that HIF1α levels were not altered at the transcriptomic level, we postulate that during normal oxygen levels, excess prolyl hydroxylase (PHD) enzymes are free to bind HIF1α due to the dramatic reduction in EPAS1 expression. PHD enzymes typically catalyze the hydroxylation of two conserved proline residues in both HIF-2α and HIF-1α.[39] Similarly, increased hydroxylation of HIF-1α in the absence of strong expression of HIF2α by Von Hippel-Lindau (pVHL) protein could be predicted. Typically, the binding of HIF-α proteins to pVHL results in poly-ubiquitination and degradation by the 26S proteasome.[40] Reductions in the levels of HIF1α and HIF2α have been widely reported to have potent anti-proliferative effects.[41–43] These data indicate that the anti-angiogenic effects of EFEMP1 knockdown may be beneficial in a range of ocular pathologies associated with aberrant angiogenesis including retinopathy of prematurity and diabetic retinopathy in addition to neovascular AMD.

EFEMP1 has been shown to be upregulated in the retinal and choroidal tissues of AMD patients.[27] Herein, we confirmed this upregulation of EFEMP1 in human RPE/choroid from donors with AMD, and report EFEMP1 ASOs can significantly reduce choroidal explant growth capacity from human eyes with or without either dry or wet AMD. The choroidal explant sprouting assay has been used with mouse choroidal tissue and found to be a robust and reproducible system. In this study, we adapted the methodology for human choroidal explants, which display a longer duration than the mouse system and exhibit more consistent results. This enabled testing of EFEMP1 ASO in both mouse and human tissue, increasing its translational value.

ASOs are short, synthetic nucleic acid sequences designed to bind to specific RNA targets and modulate gene expression.[44] By binding to the mRNA of EFEMP1, ASOs can reduce EFEMP1 expression through RNAseH1 recruiting promoting EFEMP1 mRNA degradation, potentially reducing the pathological effects associated with

its overexpression. This approach offers several advantages over traditional therapies. Unlike anti-VEGF agents, which target downstream effectors of angiogenesis,[29] EFEMP1-targeted ASOs address a key upstream factor involved in the regulation of angiogenic signaling pathways, potentially providing an opportunity for earlier intervention and a means of controlling CNV development. Furthermore, ASOs are able to offer a sustained therapeutic effect, reducing the need for frequent injections and improving patient compliance.[45]

Several challenges and considerations exist when evaluating the clinical use of EFEMP1-targeted ASOs in ADD and AMD. One significant issue is the delivery and stability of the ASOs within the retinal tissues. We have shown significant knockdown in the posterior segment of the eye; however, future studies should explore the tissue distribution of the ASO. Furthermore, cationic polymers or lipid nanoparticles could be assessed as potential carriers for ASO studies in the eye, to improve delivery of ASOs to retinal cells at therapeutic concentrations. Additionally, the safety profile of EFEMP1-targeted ASOs must be thoroughly assessed. While ASOs generally have a favorable safety profile compared to small molecule drugs, long-term effects, and potential off-target interactions, there have been reports of ASO-associated lens events in several clinical trials.[46] These effects may be chemistry or sequence-dependent, but careful attention will need to be given to any off-target effects *in vivo*.

An alternative approach could focus on an allele-specific knockdown of EFEMP1 R345W. However, for single missense variants that differ by a single nucleotide, this can be challenging as the mutation location may not be optimal for ASO binding activity and reduces the number of possible candidate sequences. It is also unclear if the knockdown of a single allele would be sufficient to reduce pathology during clinically relevant ADD once the drusen has begun to form. Given the high levels of EFEMP1, both intracellularly and extracellularly, it could be informative in future studies to understand the relative abundance of EFEMP1 and EFEMP1 R345W. In support of a biallelic approach, patients with biallelic variants in EFEMP1 that result in a near-complete loss of gene expression have been reported to have normal retinal structure, suggesting retinal knockdown of EFEMP1 may not be associated with pathology.[47] There have been reports of myopia; however, the causality has not been determined to date, this may be a developmental abnormality that may not occur in adult knockdown; however, further research will be needed to determine this. Furthermore, EFEMP1 knockout mice have been found to have normal retinal structure and have been found to protective from sub-RPE deposit formation.[8]

Several limitations of this study should be acknowledged, including the use of ARPE19 cells as a model for screening ASOs. ARPE19 cells provide versatility for *in vitro* genetic manipulation and production of a stable cell culture and act in this study as a research tool to screen a library of EFEMP1-targeted ASOs that would be challenging to perform in primary cells. ADD patient-derived iPSC-RPE would be considered the gold standard *in vitro* model and are not commercially available and were not possible to obtain for this study due to their rarity. However, it is unlikely that using these cells would impact our identification of potent EFEMP1-targeted ASOs that we have now validated *in vitro*, in human iPSC-RPE, primary endothelial cells, in mouse tissue *ex vivo*, and *in vivo* and finally in *ex vivo* human choroidal tissue from AMD donors. Long-term iPSC-RPE culture studies exploring the impact of EFEMP1 overexpression would be beneficial to understand its role in oxidative stress pathways. Future studies should now explore the potential of a combination of EFEMP1-targeted ASOs with anti-VEGF therapy, which may lead to prolonged efficacy while reducing frequency of administration. This is based on the notion that EFEMP1 overexpression may underpin several early pathways that modulate the development of new blood vessels. These data have highlighted a potential role of RPE cells in mediating neovascularization in ADD; future studies should investigate the cross talk between mutant RPE cells with endothelial cells.

In conclusion, we have identified and tested a potent EFEMP1-targeted ASO that offers a promising new avenue for treating ADD and potentially AMD. While preclinical data *in vitro*, *ex vivo*, and *in vivo* are encouraging, further research using *in vivo* models of ADD and AMD-related pathology will be required to assess the full potential of EFEMP1-targeted ASOs and determine the safety, efficacy, and optimal *in vivo* dose. If successful, this approach could add to the existing treatment options, potentially improving outcomes for patients with these debilitating diseases.

MATERIALS AND METHODS

Cell culture

ARPE19 cells were purchased from ATCC (CRL-2302). RPE media was prepared as previously described.[48] Briefly, minimum essential medium alpha (MEM α) (Thermo Fisher Scientific; 12571-063) was supplemented with 1:100 N2 Medium Supplement (Gibco; 17502048), 1:100 glutamine (Lonza; BEBP17-605E), 1:100 non-essential amino acid solution (Gibco; 11140050), 20 μg/L hydrocortisone (Millipore Sigma; H6909), 250 mg/L taurine (Millipore Sigma; T0625) and 0.013 μg/L of triiodo-thyronin (Millipore Sigma; T5516) with, or without 5% heat-inactivated fetal bovine serum (FBS) (HyClone; SH30071.03) were indicated. About 100 μg/mL Normocin (InvivoGen; ant-nr-1) was used as an antibiotic and antifungal agent. For bulk RNA-seq, qPCR and western blotting ARPE19 and ARPE19 R345W cells were grown and differentiated in 6-well transwells for 30 days.

iPSC-RPE (Cellular Dynamics International; R1101) were cultured in media composed of MEM α, 5% KnockOut SR* (Thermo Fisher Scientific; 10828-028), 55 nM Hydrocortisone (Millipore Sigma, H6909), 14 pg/mL Triiodo-L-thyronine (T3) (Millipore Sigma; T5516), 1% *N*-2 Supplement (Gibco; 17502048), and 250 μg/mL Taurine (Sigma; T0625) final concentration. For cell culture, plates are coated with 2.5 μg/mL vitronectin (STEMCELL Technologies; 07180), cells are plated at a concentration of 0.5×10^6 cells/mL. Media is replaced every 2 days RPE are matured per manufacturers guidelines.

Primary HMRECs were purchased at P3 (Cell Systems; ACBRI 18) and cultured at 37°C in 5% CO2 in endothelial cell growth basal medium (EBM-2) (Lonza, CC-3156) supplemented with the Endothelial Growth Media (EGM)-2 SingleQuot kit (Lonza CC-4176) consisting of 0.04% hydrocortisone, 0.4% human fibroblast growth factor B (hFGF-B), 0.1% VEGF, 0.1% recombinant analog of insulin-like growth factor (R3 IGF-1), 0.1% ascorbic acid, 0.1% human epidermal growth factor (hEGF), 0.1% gentamicin/amphotericin solution (GA-1000) and 0.1% heparin. The media was also supplemented with 2% FBS (Atlanta Biologicals) 1% penicillin/streptomycin (Thermo Fisher Scientific; 15140122), and 1% L-glutamine (Lonza; BEBP17-605E). Transfections were performed at P7 in media omitting antibiotics for 48 h prior to being returned to normal media.

CRISPR-Cas9 gene-editing of ARPE19s

CRISPR-Cas9 technology was utilized to introduce a specific point mutation at codon 345 of the EFEMP1 gene through a homology-directed repair approach. This mutation, R345W (arginine to tryptophan), has been associated with ADD, making it a valuable model for studying pathophysiological mechanisms in RPE cells.[49] The EFEMP1 gene at the precise location of interest using the sgRNA-seq 5′-GACC ACAAATGAATGCCGGG-3′ and the single-stranded oligodeoxynucleotide (ssODN): 5′–A*T*ATAAATGAGTGTGAGACCACAAAT GAATGCTGGGAGGATGAAATGTGTTGGAATTATCATG*G *C – 3′. The sgRNA-seq is then introduced into ARPE19 cells, a human retinal pigment epithelial cell line, along with the Cas9 endonuclease. Following successful editing, the mutant cells are selected and validated using techniques such as PCR and sequencing to confirm the presence of the EFEMP1 R345W mutation. These mutant ARPE19 cells can then be used to study the molecular and cellular effects of the mutation, providing insights into retinal disease mechanisms and potential therapeutic strategies.

ASOs synthesis

ASOs were synthesized using solid-phase phosphoramidite chemistry, incorporating phosphorothioate (PS) backbone and 2′-O-methoxyethyl (2′-MOE) sugar modifications. The ASOs were purified using reversed-phase high-performance liquid chromatography (HPLC) followed by desalting. ASOs utilized were endotoxin-free with greater than 80% purity and produced by Axolabs GmbH. The 20-nucleotide 5–10–5 gapmer ASOs (EFEMP1 ASO sequence: 5′-CTGTCAGCATC TCCAGGTCC-3′) and ASO mismatch (5′-CTGTCAGCCTCTTCAG GTCC-3′) were synthesized with a fully PS backbone. The central ten DNA nucleotides were flanked by five 2′MOE-modified ribonucleotides at each terminus. This configuration enables RNase H-mediated cleavage of the target RNA and confers enhanced nuclease resistance and metabolic stability. All 247 sequences and associated data can be accessed in patent (methods and compositions for targeting Efemp1: WO2023154964A1).

RNA-seq library preparation and sequencing

RNA-seq analyses were performed for each condition with three biological replicates, each consisting of 100,000 cells. Total RNA was extracted from using the RNeasy Plus Mini Kit (QIAGEN; 74134), following the manufacturer's instructions. RNA quality was assessed using a Tapestation and samples with an RNA integrity number (RIN) > 8 were used for sequencing. RNA libraries were constructed by Simches Research Center, Next Generation Sequencing Core, Massachusetts General Hospital (Boston, MA, USA) using a NEBNext Ultra II Directional RNA Library Prep Kit (Ipswich, MA, USA). The library was sequenced on the Illumina NextSeq 2000 sequencer platform. Sequences consisted of 50 bp paired-end reads to a minimum depth of 30 million reads per sample.

RNA-seq data analysis

Raw read sequences were analyzed for quality using FastQC[50] and MultiQC.[51] Adapters and low-quality bases were trimmed using Cutadapt 3.4[52] and Trim Galore 0.6.5-1 with the parameters -q 20 –phred33 –length 20 Babraham Bioinformatics-Trim Galore! Available online: https://www.bioinformatics.babraham.ac.uk/projects/trim_galore accessed in August 2024. Human genome GRCh38 was indexed using Hisat2 (2.1.0–4),[53] incorporating splice junctions from Gencode GTF gencode.v.46.primary_assembly.annotation.gtf file.[54] Stringtie 2.1.5[55] and gencode GTF annotation gencode. v.46.primary_assembly.annotation.gtf was used to generate gene counts. DESeq2 1.34.0[56] was used to determine differential expression. DEGs were defined by an adjusted p value $\leq$0.05, and $\log_2$ fold change (LFC) $\geq$ 1.5. For DEGs, the p value was adjusted using Benjamin-Hochberg correction individually for each pairwise comparison. Metascape, was used for GO analysis.[57] All figures were generated in the R 4.4.1 environment.

GSEA

The normalized count matrix obtained from DESeq2 was used for GSEA v.4.3.2.[51,58] GSEA was performed using 1,000 permutations and gene set permutations with gene set size filters: min = 15 and max = 500. Both the Hallmark gene set and Reactome C2 gene set were used for analysis.

Lentiviral processing and transduction

iPSc-derived RPE cells were plated and 24 h after seeding, the cells were incubated (MOI:5, 10, and 15) with lentiviral constructs under a CMV promoter the transductions were carried out in the presence of 8 μg/mL polybrene to enhance cellular uptake. After the transduction mix was added to the cells. Ater 24 h of treatment, the transduction is replaced with fresh media. Transduction efficiency was assessed using the EVOS M7000 imaging system (Thermo Fisher Scientific, Waltham, MA, USA) using the green fluorescent protein (GFP) channel at 20× magnification.

RNA isolation and RT-qPCR

Unless otherwise stated, cells for quantitative reverse-transcription PCR (RT-qPCR) analysis were treated for 48 h before being lysed. RNA was extracted using RNeasy Micro Kits (QIAGEN; 74004) according to the manufacturer's instructions. Transcription into complementary DNA was performed using the iScript cDNA synthesis kit (Bio-Rad; 1708890), following the manufacturers'

protocol, and probed using SsoAdvanced Universal SYBR Green Supermix (Bio-Rad; 1725274) in 384-well plates (Bio-Rad, HSP3805). Logarithm validated premixed primers for selected genes (Table S4) were purchased from Integrated DNA Technologies (Coralville, IA, USA). Quantification Cycle (C_q) values were determined for control and experimental samples. Changes in mRNA expression were normalized to housekeeping genes including beta2 microtubulin (B2M), mRNA expression using the $2^{-\Delta\Delta Cq}$ calculations using CFX Maestro Software 2.3 (Bio-Rad; 12013758). RNA was extracted and purified using a RNeasy Plus Micro kit (QIAGEN; 74034). Briefly, eye posterior chamber in RLT buffer supplemented with 3% of β-mercaptoethanol (Millipore Sigma; 444203) was lysed with a hand homogenizer. Samples were centrifuged at maximum speed and supernatant was collected for RNA purification as per manufacturer instructions. RNA concentration was measured by Nanodrop.

Western blotting

A total of 20 μg of cell lysates were prepared in 4 μL of 1 M DTT (Millipore Sigma; 646563) and 10 μL Laemmli buffer (Boston BioProducts; BP-110R) to a final volume of 40 μL and denatured with heat for 5 min at 95°C. Samples were fractionated for 70 V for 15 min and further for 100 V for 1 h using Criterion precast 4%–20% gels (Bio-Rad; 5671093) and Tris-Glycine-Sodium Dodecyl Sulfate (Tris-Glycine-SDS) running buffer (Bio-Rad; 610771EDU). For multiplexing applications, proteins were transferred to polyvinylidene fluoride membranes (PVDF) (Thermo Fisher Scientific; IB23001), using the iBlot 2 dry transfer system (Thermo Fisher Scientific; IB21002S). Total protein levels for this application were evaluated using Revert 700 Total Protein Stain (Thermo Fisher Scientific; NC1683155) according to the manufacturer's instructions. Membranes were blocked for 1 h with Intercept Blocking Tris-Buffered Saline (TBS) Buffer (LI-CORbio; 927–60,001) for 2 h. Membranes were then incubated in primary antibodies in blocking buffer for either 2 h at room temperature or 18 h at 4°C (Table S5). Blots were washed thrice with TBS-Tween 20 (TBS-T) buffer for 10 min (Thermo Fisher Scientific; 28360) and incubated with secondary antibodies in blocking buffer for 1 h at room temperature (Table S5). Immunoreactive bands were detected using the Odyssey Infrared Imaging System (LI-CORbio; Lincoln, NE, USA) and visualized on the Image Studio Acquisition Software (v.2.1; LI-CORbio). For blots analyzed using chemiluminescence, blots were transferred to PVDF using the *trans*-blot Turbo transfer system (Bio-Rad; 1704150) and *trans*-blot Turbo RTA Midi PVDF Transfer Kit (Bio-Rad, 1704273) according to the manufacturer's instructions. Membranes were reversibly stained to assess protein loading (Thermo Fisher Scientific; 24585), blocked in 5% milk in TBS for 1 h at repetition time (RT) and incubated overnight at 4°C in the appropriate primary antibodies in blocking buffer (Table S5). Blots were incubated with the corresponding HRP-conjugated secondary antibodies (Table S5) at RT and washed as described above. Blots were developed using Clarity ECl Western Substrate (Bio-Rad; 1705061) and visualized using an iBright FL1500 imaging system (Thermo Fisher Scientific; A44241).

Immunocytochemistry

iPSC-RPE cells were seeded on glass slides (Mat-Tek; CCS-4), 40,000 cells per well. Cells were reverse transfected with EFEMP1-targeted ASOs, in serum free media and incubated for 2 h. The media was exchanged with 500 μL growth media and incubated for 24 h at 37°C. Cells were washed with ice-cold PBS, fixed in 4% paraformaldehyde (PFA) for 5 min, washed in PBS and incubated in permeabilization and blocking buffer (PB) consisting of 1× PBS, 1% Normal Donkey Serum (Millipore Sigma; D9663) and 0.05% Triton X-100 (Millipore Sigma; T8787) overnight. Cells were then incubated with primary antibody (Table S5) diluted in blocking buffer overnight at 4°C. Samples were washed in PBS and incubated with the corresponding secondary antibody (Table S5) for 2 h at room temperature, and nuclei were stained with DAPI (1:5,000) for 15 min. Finally, samples were mounted with Vectashield mounting media (Vector Laboratories; H-1000). Images were acquired with a Leica SP8 confocal microscope (Leica Camera AG, Wetzlar, Germany).

Watershed cell segmentation

Image processing was performed using MATLAB R2021a. The watershed transform was applied to Gaussian filtered images, which we also imposed local minima on to avoid over-segmentation. Images of the cell membranes were smoothed using a Gaussian filter with a sigma of 3 pixels. Local minima were generated via the H-minima transform (using the MATLAB function imextendedmin with a minimum depth of 4 intensity units).

Human choroidal explants

The Schepens Eye Research Institute Institutional Review Board reviewed the experiments conducted on postmortem human eye material and deemed them exempt from institutional review board (IRB) approval (REDCap ID#910). This exemption was granted as the research does not involve human subjects. The postmortem donor eye globes were procured from the Minnesota Lions Gift of Sight Eye Bank (Saint Paul, MN, USA). Cultrex Basement Membrane Extract (BME) (R&D Systems; 3432-010-01) was thawed on ice for 24 h prior to the experiment. Postmortem human eyes, provided by the Lions Gift of Sight Eye Bank, were obtained within 24 h and immediately dissected. Eyes were cut circumferentially about 1.0 mm posterior to the limbus and the cornea, lens, iris, retina, conjunctiva, and sclera were removed leaving the RPE/choroid behind. The remaining RPE/choroid was transferred to explant culture medium composed of media composed of MCDB 131 (Thermo Fisher Scientific; 10372-019), EGM 2 SingleQuot Kit supplement and Growth factors (Lonza; CC-4176), 12% FBS (Atlanta Biologicals; S11150), 1% Penicillin/Streptomycin (Lonza; 17-602E), and 1% L-glutamine (Lonza; BE17-605E/U1). Using a 1 mm biopsy punch (Thermo Fisher Scientific; 12-460-401), 1 mm circular punches were generated circumferentially, equidistant from the optic disk. Punches were then embedded in 30 μL domes of Cultrex BME in 24-well tissue culture plates (CellTreat; 229124) and allowed to set for 45 min at 37°C, 5% CO_2. Around 500 μL of media was gently added to each explant, incubating at 37°C, 5% CO_2. Upon sprouting, growth medium was exchanged for media omitting antibiotics.

Transfections were performed using 25 nM ASO mismatch control (M2) or EFEMP1 ASO diluted in optiMEM reduced serum media (ThermoFisher Scientific; 31985070). Diluted constructs were packaged using Lipofectamine RNAiMAX Transfection reagent (Thermo Fisher Scientific; 13778150) in optiMEM, and administered to the explants for 48 h before the media was exchanged with normal growth medium. Following transfection, the outgrowth of the explants was monitored by bright field microscopy using a bright-field microscope (Olympus CKX53, Olympus, Tokyo, Japan). Media was changed and bright field images were taken every other day for 12 consecutive days. Transfections were repeated between days 6 and 7 to maintain the knockdown effect. To further observe 3D outgrowth and demonstrate viability, explants were also stained with 1 mM Calcein AM cell-permeable dye (Thermo Fisher Scientific; C34852) on day 12. Stained explants were observed using contiguous rectangular regions at 10× magnification with a confocal microscope (Leica SP8, Leica, Wetzlar, Germany). Images from each region were taken at a depth of ∼6–8 μm. Bright field and fluorescent images were quantified using FIJI-ImageJ2 software (National Institutes of Health, Bethesda, MD, USA) and the SWIFT-Choroid computerized quantification method.[28]

Mouse choroidal explants

Mouse choroidal explants were prepared using an established *ex vivo* choroidal sprouting assay with minor modifications.[28,59] Eyes were enucleated from C57BL/6J mice and immediately transferred to ice-cold sterile PBS. Under a dissecting microscope, the anterior segment, lens, retina, and vitreous were carefully removed to isolate the posterior eyecup containing the RPE/choroid/sclera complex. The RPE/choroid was separated and cut into 1 mm circular punches using a sterile biopsy punch. Mouse explants were then treated as described earlier.

Intravitreal injections

Experiments utilizing live vertebrates were reviewed and approved by the Institutional Animal Care and Use Committee (Schepens Eye Research Institute) and carried out according to the National Institutes of Health Guide for the Care and use of Laboratory Animals. All studies were performed in compliance with ARRIVE guidelines. Mice were anesthetized by intraperitoneal (i.p.) injection of a ketamine/xylazine mixture. A single intravitreal injection of either ASO mismatch or EFEMP1 ASO animals was administered. Animals were euthanized at predefined timepoints and samples were collected for further analysis.

Statistics

Western blots were quantified using ImageJ software v.13 (National Institutes of Health). Results are expressed as mean ± standard error of the mean (SEM). Statistical analyses were performed using GraphPad Prism v.10.1.2 software, with p values < 0.05 defined as statistically significant. Residuals of raw data were initially tested for normality to determine whether to apply parametric or nonparametric tests. Parametric data were analyzed overall with the Student's t test or one-way or two-way ANOVA followed by Tukey's test for multiple comparisons unless stated otherwise. Rank correlation was measured by Pearsons's rank correlation coefficient. Nonparametric data were analyzed by Mann-Whitney U test, Kruskal-Wallis or Friedman's test conducted with Dunn's post hoc test for multiple comparisons. Rank correlation for nonparametric data was performed using Spearman's rank correlation coefficient.

DATA AND CODE AVAILABILITY

The bioinformatics data discussed in this publication have been deposited in NCBIs Gene Expression Omnibus[60] and are accessible through GEO Series accession number GSE289976 (https://www.ncbi.nlm.nih.gov/geo/query/acc.cgi?acc=GSE289976). All code or data will be made available upon reasonable request to the authors.

ACKNOWLEDGMENTS

We would like to thank the Massachusetts Lions Eye Research Fund for supporting this research through the young investigator program awarded to M.O.H. We would also like to acknowledge the Schepens Eye Research Institute (SERI) morphology core for their services (supported by National Eye Institute core grant P30EYE003790) and thank the Enterprise Research Infrastructure & Services (ERIS) at Mass General Brigham for their in-depth support and for the provision of computational resources.

AUTHOR CONTRIBUTIONS

Project conceptualization was performed by A.P., V.V., C.L., and M.O.H.; methodology for experimental approaches was established by W.P.M., S.A.-A., P.P.-C., A.L., S.A., C.L., and M.O.H.; investigations were carried out by W.P.M., S.A.-A., P.P.-C., R.L., A.U., J.S.-S., T.E.V., C.M., D.S., M.S., J.V., A.M.-T., X.H., Y.A.-R., B.A.K., K.W.B., A.L.G., A.V., R.S., and M.O.H.; visualization of experimental endpoints was performed M.O.H., W.P.M., S.A.-A., P.P.-C., R.L., A.U., J.S.-S., T.E.V., D.S., M.S., J.V., B.A.K., K.W.B., A.L.G., A.V., R.S., A.L., and M.O.H.; funding was acquired by M.O.H.; project administration was the responsibility of M.O.H. and C.L.; research was supervised by M.O.H.; the manuscript was written by W.P.M., S.A.-A., P.P.-C., and M.O.H.; the manuscript was reviewed and edited by W.P.M., S.A.-A., P.P.-C., C.M., A.L., L.A.K., J.F.A.-V., A.P., S.A., V.V., C.L., and M.O.H.

DECLARATION OF INTERESTS

A.P., S.A., and C.L. are co-founders and shareholders of Aldebaran Therapeutics.

SUPPLEMENTAL INFORMATION

Supplemental information can be found online at https://doi.org/10.1016/j.omtn.2026.103024.

REFERENCES

1. Garland, D.L., Fernandez-Godino, R., Kaur, I., Speicher, K.D., Harnly, J.M., Lambris, J.D., Speicher, D.W., and Pierce, E.A. (2014). Mouse genetics and proteomic analyses demonstrate a critical role for complement in a model of DHRD/ML, an inherited macular degeneration. Hum. Mol. Genet. *23*, 52–68. https://doi.org/10.1093/hmg/ddt395.

2. Fernandez-Godino, R., Bujakowska, K.M., and Pierce, E.A. (2018). Changes in extracellular matrix cause RPE cells to make basal deposits and activate the alternative complement pathway. Hum. Mol. Genet. *27*, 147–159. https://doi.org/10.1093/hmg/ddx392.

3. Fu, L., Garland, D., Yang, Z., Shukla, D., Rajendran, A., Pearson, E., Stone, E.M., Zhang, K., and Pierce, E.A. (2007). The R345W mutation in EFEMP1 is pathogenic and causes AMD-like deposits in mice. Hum. Mol. Genet. *16*, 2411–2422. https://doi.org/10.1093/hmg/ddm198.

4. Marmorstein, L.Y., McLaughlin, P.J., Peachey, N.S., Sasaki, T., and Marmorstein, A.D. (2007). Formation and progression of sub-retinal pigment epithelium deposits in Efemp1 mutation knock-in mice: a model for the early pathogenic course of macular degeneration. Hum. Mol. Genet. *16*, 2423–2432. https://doi.org/10.1093/hmg/ddm199.

5. Marmorstein, L.Y., Munier, F.L., Arsenijevic, Y., Schorderet, D.F., McLaughlin, P.J., Chung, D., Traboulsi, E., and Marmorstein, A.D. (2002). Aberrant accumulation of

EFEMP1 underlies drusen formation in Malattia Leventinese and age-related macular degeneration. Proc. Natl. Acad. Sci. USA *99*, 13067–13072. https://doi.org/10.1073/pnas.202491599.

6. Stone, E.M., Lotery, A.J., Munier, F.L., Héon, E., Piguet, B., Guymer, R.H., Vandenburgh, K., Cousin, P., Nishimura, D., Swiderski, R.E., et al. (1999). A single EFEMP1 mutation associated with both Malattia Leventinese and Doyne honeycomb retinal dystrophy. Nat. Genet. *22*, 199–202. https://doi.org/10.1038/9722.

7. de Guimarães, T.A.C., Kalitzeos, A., Mahroo, O.A., van der Spuy, J., Webster, A.R., and Michaelides, M. (2024). A Long-Term Retrospective Natural History Study of EFEMP1-Associated Autosomal Dominant Drusen. Investig. Ophthalmol. Vis. Sci. *65*, 31. https://doi.org/10.1167/iovs.65.6.31.

8. Stanton, J.B., Marmorstein, A.D., Zhang, Y., and Marmorstein, L.Y. (2017). Deletion of Efemp1 Is Protective Against the Development of Sub-RPE Deposits in Mouse Eyes. Investig. Ophthalmol. Vis. Sci. *58*, 1455–1461. https://doi.org/10.1167/iovs.16-20955.

9. Wyatt, M.K., Tsai, J.Y., Mishra, S., Campos, M., Jaworski, C., Fariss, R.N., Bernstein, S.L., and Wistow, G. (2013). Interaction of complement factor h and fibulin3 in age-related macular degeneration. PLoS One *8*, e68088. https://doi.org/10.1371/journal.pone.0068088.

10. Nesargikar, P.N., Spiller, B., and Chavez, R. (2012). The complement system: history, pathways, cascade and inhibitors. Eur. J. Microbiol. Immunol. *2*, 103–111. https://doi.org/10.1556/EuJMI.2.2012.2.2.

11. Klenotic, P.A., Munier, F.L., Marmorstein, L.Y., and Anand-Apte, B. (2004). Tissue inhibitor of metalloproteinases-3 (TIMP-3) is a binding partner of epithelial growth factor-containing fibulin-like extracellular matrix protein 1 (EFEMP1). Implications for macular degenerations. J. Biol. Chem. *279*, 30469–30473. https://doi.org/10.1074/jbc.M403026200.

12. Weber, B.H., Vogt, G., Pruett, R.C., Stöhr, H., and Felbor, U. (1994). Mutations in the tissue inhibitor of metalloproteinases-3 (TIMP3) in patients with Sorsby's fundus dystrophy. Nat. Genet. *8*, 352–356. https://doi.org/10.1038/ng1294-352.

13. Sohn, E.H., Wang, K., Thompson, S., Riker, M.J., Hoffmann, J.M., Stone, E.M., and Mullins, R.F. (2015). Comparison of drusen and modifying genes in autosomal dominant radial drusen and age-related macular degeneration. Retina *35*, 48–57. https://doi.org/10.1097/iae.0000000000000263.

14. Curcio, C.A. (2018). Soft Drusen in Age-Related Macular Degeneration: Biology and Targeting Via the Oil Spill Strategies. Investig. Ophthalmol. Vis. Sci. *59*, AMD160–AMD181. https://doi.org/10.1167/iovs.18-24882.

15. Curcio, C.A., and Johnson, M. (2013). Structure, function, and pathology of Bruch's membrane. Retina *1*, 466–481.

16. Curcio, C.A., Presley, J.B., Malek, G., Medeiros, N.E., Avery, D.V., and Kruth, H.S. (2005). Esterified and unesterified cholesterol in drusen and basal deposits of eyes with age-related maculopathy. Exp. Eye Res. *81*, 731–741. https://doi.org/10.1016/j.exer.2005.04.012.

17. Fritsche, L.G., Igl, W., Bailey, J.N.C., Grassmann, F., Sengupta, S., Bragg-Gresham, J.L., Burdon, K.P., Hebbring, S.J., Wen, C., Gorski, M., et al. (2016). A large genome-wide association study of age-related macular degeneration highlights contributions of rare and common variants. Nat. Genet. *48*, 134–143. https://doi.org/10.1038/ng.3448.

18. Sura, A.A., Chen, L., Messinger, J.D., Swain, T.A., McGwin, G., Jr., Freund, K.B., and Curcio, C.A. (2020). Measuring the Contributions of Basal Laminar Deposit and Bruch's Membrane in Age-Related Macular Degeneration. Investig. Ophthalmol. Vis. Sci. *61*, 19. https://doi.org/10.1167/iovs.61.13.19.

19. Heier, J.S., Lad, E.M., Holz, F.G., Rosenfeld, P.J., Guymer, R.H., Boyer, D., Grossi, F., Baumal, C.R., Korobelnik, J.F., Slakter, J.S., et al. (2023). Pegcetacoplan for the treatment of geographic atrophy secondary to age-related macular degeneration (OAKS and DERBY): two multicentre, randomised, double-masked, sham-controlled, phase 3 trials. Lancet *402*, 1434–1448. https://doi.org/10.1016/s0140-6736(23)01520-9.

20. Khanani, A.M., Patel, S.S., Staurenghi, G., Tadayoni, R., Danzig, C.J., Eichenbaum, D.A., Hsu, J., Wykoff, C.C., Heier, J.S., Lally, D.R., et al. (2023). Efficacy and safety of avacincaptad pegol in patients with geographic atrophy (GATHER2): 12-month results from a randomised, double-masked, phase 3 trial. Lancet *402*, 1449–1458. https://doi.org/10.1016/s0140-6736(23)01583-0.

21. Witkin, A.J., Jaffe, G.J., Srivastava, S.K., Davis, J.L., and Kim, J.E. (2024). Retinal Vasculitis After Intravitreal Pegcetacoplan: Report From the ASRS Research and Safety in Therapeutics (ReST) Committee. J. Vitreoretin. Dis. *8*, 9–20. https://doi.org/10.1177/24741264231220224.

22. Gait, M.J., and Agrawal, S. (2022). Introduction and History of the Chemistry of Nucleic Acids Therapeutics. Methods Mol. Biol. *2434*, 3–31. https://doi.org/10.1007/978-1-0716-2010-6_1.

23. Roehr, B. (1998). Fomivirsen approved for CMV retinitis. J. Int. Assoc. Physicians AIDS Care *4*, 14–16.

24. Agrawal, S. (2020). RNA Therapeutics Are Stepping Out of the Maze. Trends Mol. Med. *26*, 1061–1064. https://doi.org/10.1016/j.molmed.2020.08.007.

25. Ruchi, R., Gupta, K., Verma, R., Manautou, J., Zhong, X.B., and Bahal, R. (2026). The impact of antisense oligonucleotide (ASO) therapeutics on the future of rare disease drug discovery. Expert Opin. Drug Discov. *21*, 369–373. https://doi.org/10.1080/17460441.2026.2652440.

26. Duggan, M.R., Yang, Z., Cui, Y., Dark, H.E., Wen, J., Erus, G., Hohman, T.J., Chen, J., Lewis, A., Moghekar, A., et al. (2024). Proteomic analyses reveal plasma EFEMP1 and CXCL12 as biomarkers and determinants of neurodegeneration. Alzheimer's Dement. *20*, 6486–6505. https://doi.org/10.1002/alz.14142.

27. Cheng, L., Chen, C., Guo, W., Liu, K., Zhao, Q., Lu, P., Yu, F., and Xu, X. (2020). EFEMP1 Overexpression Contributes to Neovascularization in Age-Related Macular Degeneration. Front. Pharmacol. *11*, 547436. https://doi.org/10.3389/fphar.2020.547436.

28. Shao, Z., Friedlander, M., Hurst, C.G., Cui, Z., Pei, D.T., Evans, L.P., Juan, A.M., Tahiri, H., Duhamel, F., Chen, J., et al. (2013). Choroid sprouting assay: an ex vivo model of microvascular angiogenesis. PLoS One *8*, e69552. https://doi.org/10.1371/journal.pone.0069552.

29. Wang, L., Liu, W.Q., Broussy, S., Han, B., and Fang, H. (2023). Recent advances of anti-angiogenic inhibitors targeting VEGF/VEGFR axis. Front. Pharmacol. *14*, 1307860. https://doi.org/10.3389/fphar.2023.1307860.

30. Wong, R.S.M., Navarro-Cabrera, J.R., Comia, N.S., Goh, Y.T., Idrobo, H., Kongkabpan, D., Gómez-Almaguer, D., Al-Adhami, M., Ajayi, T., Alvarenga, P., et al. (2023). Pegcetacoplan controls hemolysis in complement inhibitor-naive patients with paroxysmal nocturnal hemoglobinuria. Blood Adv. *7*, 2468–2478. https://doi.org/10.1182/bloodadvances.2022009129.

31. Howard, J.F., Jr., Bresch, S., Genge, A., Hewamadduma, C., Hinton, J., Hussain, Y., Juntas-Morales, R., Kaminski, H.J., Maniaol, A., Mantegazza, R., et al. (2023). Safety and efficacy of zilucoplan in patients with generalised myasthenia gravis (RAISE): a randomised, double-blind, placebo-controlled, phase 3 study. Lancet Neurol. *22*, 395–406. https://doi.org/10.1016/s1474-4422(23)00080-7.

32. Crowley, M.A., Garland, D.L., Sellner, H., Banks, A., Fan, L., Rejtar, T., Buchanan, N., Delgado, O., Xu, Y.Y., Jose, S., et al. (2023). Complement factor B is critical for sub-RPE deposit accumulation in a model of Doyne honeycomb retinal dystrophy with features of age-related macular degeneration. Hum. Mol. Genet. *32*, 204–217. https://doi.org/10.1093/hmg/ddac187.

33. Garland, D.L., Pierce, E.A., and Fernandez-Godino, R. (2021). Complement C5 is not critical for the formation of sub-RPE deposits in Efemp1 mutant mice. Sci. Rep. *11*, 10416. https://doi.org/10.1038/s41598-021-89978-8.

34. Chekuri, A., Zientara-Rytter, K., Soto-Hermida, A., Borooah, S., Voronchikhina, M., Biswas, P., Kumar, V., Goodsell, D., Hayward, C., Shaw, P., et al. (2019). Late-onset retinal degeneration pathology due to mutations in CTRP5 is mediated through HTRA1. Aging Cell *18*, e13011. https://doi.org/10.1111/acel.13011.

35. Lin, M.K., Yang, J., Hsu, C.W., Gore, A., Bassuk, A.G., Brown, L.M., Colligan, R., Sengillo, J.D., Mahajan, V.B., and Tsang, S.H. (2018). HTRA1, an age-related macular degeneration protease, processes extracellular matrix proteins EFEMP1 and TSP1. Aging Cell *17*, e12710. https://doi.org/10.1111/acel.12710.

36. Dalvi, S., Roll, M., Chatterjee, A., Kumar, L.K., Bhogavalli, A., Foley, N., Arduino, C., Spencer, W., Reuben-Thomas, C., Ortolan, D., et al. (2024). Human iPSC-based disease modeling studies identify a common mechanistic defect and potential therapies for AMD and related macular dystrophies. Dev. Cell *59*, 3290–3305.e9. https://doi.org/10.1016/j.devcel.2024.09.006.

37. Hiddingh, L., Tannous, B.A., Teng, J., Tops, B., Jeuken, J., Hulleman, E., Boots-Sprenger, S.H., Vandertop, W.P., Noske, D.P., Kaspers, G.J.L., et al. (2014).

EFEMP1 induces γ-secretase/Notch-mediated temozolomide resistance in glioblastoma. Oncotarget *5*, 363–374. https://doi.org/10.18632/oncotarget.1620.

38. Kwak, J.H., Lee, N.H., Lee, H.Y., Hong, I.S., and Nam, J.S. (2016). HIF2α/EFEMP1 cascade mediates hypoxic effects on breast cancer stem cell hierarchy. Oncotarget *7*, 43518–43533. https://doi.org/10.18632/oncotarget.9846.

39. Nguyen, T.L., and Durán, R.V. (2016). Prolyl hydroxylase domain enzymes and their role in cell signaling and cancer metabolism. Int. J. Biochem. Cell Biol. *80*, 71–80. https://doi.org/10.1016/j.biocel.2016.09.026.

40. Chua, Y.L., Dufour, E., Dassa, E.P., Rustin, P., Jacobs, H.T., Taylor, C.T., and Hagen, T. (2010). Stabilization of hypoxia-inducible factor-1alpha protein in hypoxia occurs independently of mitochondrial reactive oxygen species production. J. Biol. Chem. *285*, 31277–31284. https://doi.org/10.1074/jbc.M110.158485.

41. Xu, R., Wang, F., Yang, H., and Wang, Z. (2022). Action Sites and Clinical Application of HIF-1α Inhibitors. Molecules *27*, 3426. https://doi.org/10.3390/molecules27113426.

42. Patel, S.A., and Simon, M.C. (2008). Biology of hypoxia-inducible factor-2alpha in development and disease. Cell Death Differ. *15*, 628–634. https://doi.org/10.1038/cdd.2008.17.

43. Loboda, A., Jozkowicz, A., and Dulak, J. (2010). HIF-1 and HIF-2 Transcription Factors - Similar but Not Identical. Mol. Cells *29*, 435–442. https://doi.org/10.1007/s10059-010-0067-2.

44. Collotta, D., Bertocchi, I., Chiapello, E., and Collino, M. (2023). Antisense oligonucleotides: a novel Frontier in pharmacological strategy. Front. Pharmacol. *14*, 1304342. https://doi.org/10.3389/fphar.2023.1304342.

45. Cideciyan, A.V., Jacobson, S.G., Ho, A.C., Swider, M., Sumaroka, A., Roman, A.J., Wu, V., Russell, R.C., Viarbitskaya, I., Garafalo, A.V., et al. (2023). Durable vision improvement after a single intravitreal treatment with antisense oligonucleotide in CEP290-LCA: Replication in two eyes. Am. J. Ophthalmol. Case Rep. *32*, 101873. https://doi.org/10.1016/j.ajoc.2023.101873.

46. Russell, S.R., Drack, A.V., Cideciyan, A.V., Jacobson, S.G., Leroy, B.P., Van Cauwenbergh, C., Ho, A.C., Dumitrescu, A.V., Han, I.C., Martin, M., et al. (2022). Intravitreal antisense oligonucleotide sepofarsen in Leber congenital amaurosis type 10: a phase 1b/2 trial. Nat. Med. *28*, 1014–1021. https://doi.org/10.1038/s41591-022-01755-w.

47. Driver, S.G.W., Jackson, M.R., Richter, K., Tomlinson, P., Brockway, B., Halliday, B.J., Markie, D.M., Robertson, S.P., and Wade, E.M. (2020). Biallelic variants in EFEMP1 in a man with a pronounced connective tissue phenotype. Eur. J. Hum. Genet. *28*, 445–452. https://doi.org/10.1038/s41431-019-0546-7.

48. Fernandez-Godino, R., and Pierce, E.A. (2018). C3a triggers formation of sub-retinal pigment epithelium deposits via the ubiquitin proteasome pathway. Sci. Rep. *8*, 9679. https://doi.org/10.1038/s41598-018-28143-0.

49. Zhou, M., Weber, S.R., Zhao, Y., Chen, H., Barber, A.J., Grillo, S.L., Wills, C.A., Wang, H.G., Hulleman, J.D., and Sundstrom, J.M. (2020). Expression of R345W-Fibulin-3 Induces Epithelial-Mesenchymal Transition in Retinal Pigment Epithelial Cells. Front. Cell Dev. Biol. *8*, 469. https://doi.org/10.3389/fcell.2020.00469.

50. Davis, M.P.A., van Dongen, S., Abreu-Goodger, C., Bartonicek, N., and Enright, A.J. (2013). Kraken: A set of tools for quality control and analysis of high-throughput sequence data. Methods *63*, 41–49. https://doi.org/10.1016/j.ymeth.2013.06.027.

51. Ewels, P., Magnusson, M., Lundin, S., and Käller, M. (2016). MultiQC: summarize analysis results for multiple tools and samples in a single report. Bioinformatics *32*, 3047–3048. https://doi.org/10.1093/bioinformatics/btw354.

52. Kim, D., Paggi, J.M., Park, C., Bennett, C., and Salzberg, S.L. (2019). Graph-based genome alignment and genotyping with HISAT2 and HISAT-genotype. Nat. Biotechnol. *37*, 907–915. https://doi.org/10.1038/s41587-019-0201-4.

53. Pertea, M., Pertea, G.M., Antonescu, C.M., Chang, T.-C., Mendell, J.T., and Salzberg, S.L. (2015). StringTie enables improved reconstruction of a transcriptome from RNA-seq reads. Nat. Biotechnol. *33*, 290–295. https://doi.org/10.1038/nbt.3122.

54. Love, M.I., Huber, W., and Anders, S. (2014). Moderated estimation of fold change and dispersion for RNA-seq data with DESeq2. Genome Biol. *15*, 550. https://doi.org/10.1186/s13059-014-0550-8.

55. Subramanian, A., Tamayo, P., Mootha, V.K., Mukherjee, S., Ebert, B.L., Gillette, M.A., Paulovich, A., Pomeroy, S.L., Golub, T.R., Lander, E.S., and Mesirov, J.P. (2005). Gene set enrichment analysis: a knowledge-based approach for interpreting genome-wide expression profiles. Proc. Natl. Acad. Sci. USA *102*, 15545–15550. https://doi.org/10.1073/pnas.0506580102.

56. Mootha, V.K., Lindgren, C.M., Eriksson, K.F., Subramanian, A., Sihag, S., Lehar, J., Puigserver, P., Carlsson, E., Ridderstråle, M., Laurila, E., et al. (2003). PGC-1alpha-responsive genes involved in oxidative phosphorylation are coordinately downregulated in human diabetes. Nat. Genet. *34*, 267–273. https://doi.org/10.1038/ng1180.

57. Zhou, Y., Zhou, B., Pache, L., Chang, M., Khodabakhshi, A.H., Tanaseichuk, O., Benner, C., and Chanda, S.K. (2019). Metascape provides a biologist-oriented resource for the analysis of systems-level datasets. Nat. Commun. *10*, 1523. https://doi.org/10.1038/s41467-019-09234-6.

58. Frankish, A., Diekhans, M., Ferreira, A.M., Johnson, R., Jungreis, I., Loveland, J., Mudge, J.M., Sisu, C., Wright, J., Armstrong, J., et al. (2019). GENCODE reference annotation for the human and mouse genomes. Nucleic Acids Res. *47*, D766–D773. https://doi.org/10.1093/nar/gky955.

59. Tomita, Y., Shao, Z., Cakir, B., Kotoda, Y., Fu, Z., and Smith, L.E.H. (2020). An Ex Vivo Choroid Sprouting Assay of Ocular Microvascular Angiogenesis. J. Vis. Exp. https://doi.org/10.3791/61677.

60. Edgar, R., Domrachev, M., and Lash, A.E. (2002). Gene Expression Omnibus: NCBI gene expression and hybridization array data repository. Nucleic Acids Res. *30*, 207–210. https://doi.org/10.1093/nar/30.1.207.

OMTN, Volume *37*

Supplemental information

Development of an antisense oligonucleotide targeting EFEMP1 in models relevant to macular degeneration

William P. Miller, Said Arevalo-Alquichire, Paula Perez-Corredor, Rose Lin, Anil Upreti, Jeysson Sanchez-Suarez, Tim E. Vanderleest, Claudia Marino, Donovan Spencer, Matthew Scribner, Jebgy Vargas, Andres Muriel-Torres, Xinyao Hu, Yvonne Adu-Rutledge, Bryan A. Kaplan, Karim W. Barake, Audrey L. Gunawan, Aruvi Vijikumar, Reeya Shrestha, Anton Lennikov, Leo A. Kim, Joseph F. Arboleda-Velasquez, Andrew Peterson, Sudhir Agrawal, Vinod Vathipadiekal, Carlos Loya, and Michael O'Hare

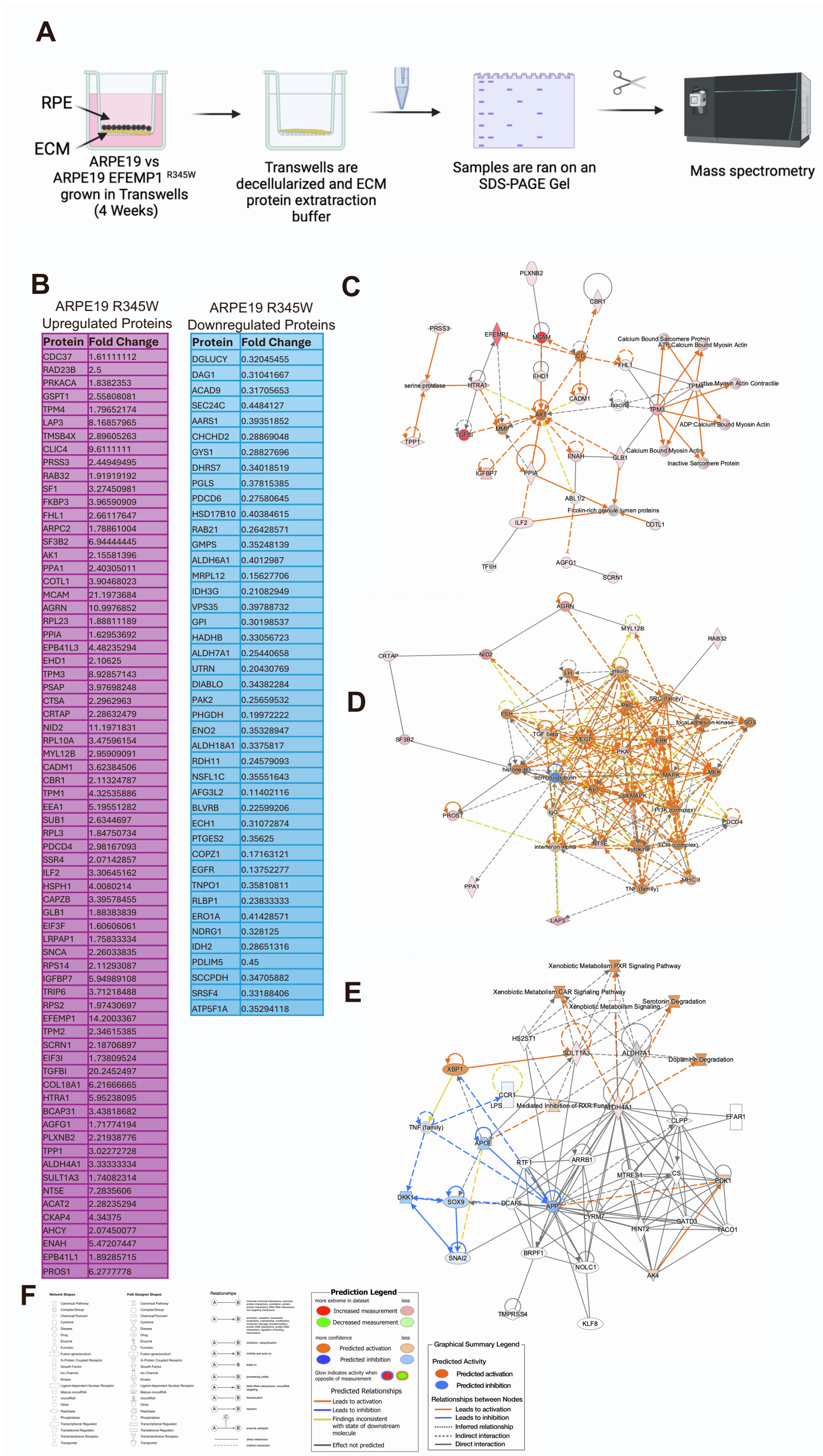

ARPE19 R345W Upregulated Proteins

Protein	Fold Change
CDC37	1.61111112
RAD23B	2.5
PRKACA	1.8382353
GSPT1	2.55808081
TPM4	1.79652174
LAP3	8.16857965
TMSB4X	2.89605263
CLIC4	9.6111111
PRSS3	2.44949495
RAB32	1.91919192
SF1	3.27450981
FKBP3	3.96590909
FHL1	2.66117647
ARPC2	1.78861004
SF3B2	6.94444445
AK1	2.15581396
PPA1	2.40305011
COTL1	3.90468023
MCAM	21.1973684
AGRN	10.9976852
RPL23	1.88811189
PPIA	1.62953692
EPB41L3	4.48235294
EHD1	2.10625
TPM3	8.92857143
PSAP	3.97698248
CTSA	2.2962963
CRTAP	2.28632479
NID2	11.1971831
RPL10A	3.47596154
MYL12B	2.95909091
CADM1	3.62384506
CBR1	2.11324787
TPM1	4.32535886
EEA1	5.19551282
SUB1	2.6344697
RPL3	1.84750734
PDCD4	2.98167093
SSR4	2.07142857
ILF2	3.30645162
HSPH1	4.0080214
CAPZB	3.39578455
GLB1	1.88383839
EIF3F	1.60606061
LRPAP1	1.75833334
SNCA	2.26033835
RPS14	2.11293087
IGFBP7	5.94989108
TRIP6	3.71218488
RPS2	1.97430697
EFEMP1	14.2003367
TPM2	2.34615385
SCRN1	2.18706897
EIF3I	1.73809524
TGFBI	20.2452497
COL18A1	6.21666665
HTRA1	5.95238095
BCAP31	3.43818682
AGFG1	1.71774194
PLXNB2	2.21938776
TPP1	3.02272728
ALDH4A1	3.33333334
SULT1A3	1.74082314
NT5E	7.2835606
ACAT2	2.28235294
CKAP4	4.34375
AHCY	2.07450077
ENAH	5.47207447
EPB41L1	1.89285715
PROS1	6.2777778

ARPE19 R345W Downregulated Proteins

Protein	Fold Change
DGLUCY	0.32045455
DAG1	0.31041667
ACAD9	0.31705653
SEC24C	0.4484127
AARS1	0.39351852
CHCHD2	0.28869048
GYS1	0.28827696
DHRS7	0.34018519
PGLS	0.37815385
PDCD6	0.27580645
HSD17B10	0.40384615
RAB21	0.26428571
GMPS	0.35248139
ALDH6A1	0.4012987
MRPL12	0.15627706
IDH3G	0.21082949
VPS35	0.39788732
GPI	0.30198537
HADHB	0.33056723
ALDH7A1	0.25440658
UTRN	0.20430769
DIABLO	0.34382284
PAK2	0.25659532
PHGDH	0.19972222
ENO2	0.35328947
ALDH18A1	0.3375817
RDH11	0.24579093
NSFL1C	0.35551643
AFG3L2	0.11402116
BLVRB	0.22599206
ECH1	0.31072874
PTGES2	0.35625
COPZ1	0.17163121
EGFR	0.13752277
TNPO1	0.35810811
RLBP1	0.23833333
ERO1A	0.41428571
NDRG1	0.328125
IDH2	0.28651316
PDLIM5	0.45
SCCPDH	0.34705882
SRSF4	0.33188406
ATP5F1A	0.35294118

Figure S1: Proteomic analysis of extracellular matrix from ARPR19 and ARPE19 R345W cells. A) Schematic of the experimental design for extracellular matrix processing of ARPE19 and

ARPE19 R345W cells for mass spectrometry analysis. **B**) Lists of proteins over 1.5-fold increased (red) in ARPE19 R345W and downregulated below 0.5-fold (blue). (n=2 per group) **C-E)** Ingenuity Pathway Analysis (IPA) networks of interest **F)** Key for IPA analysis.

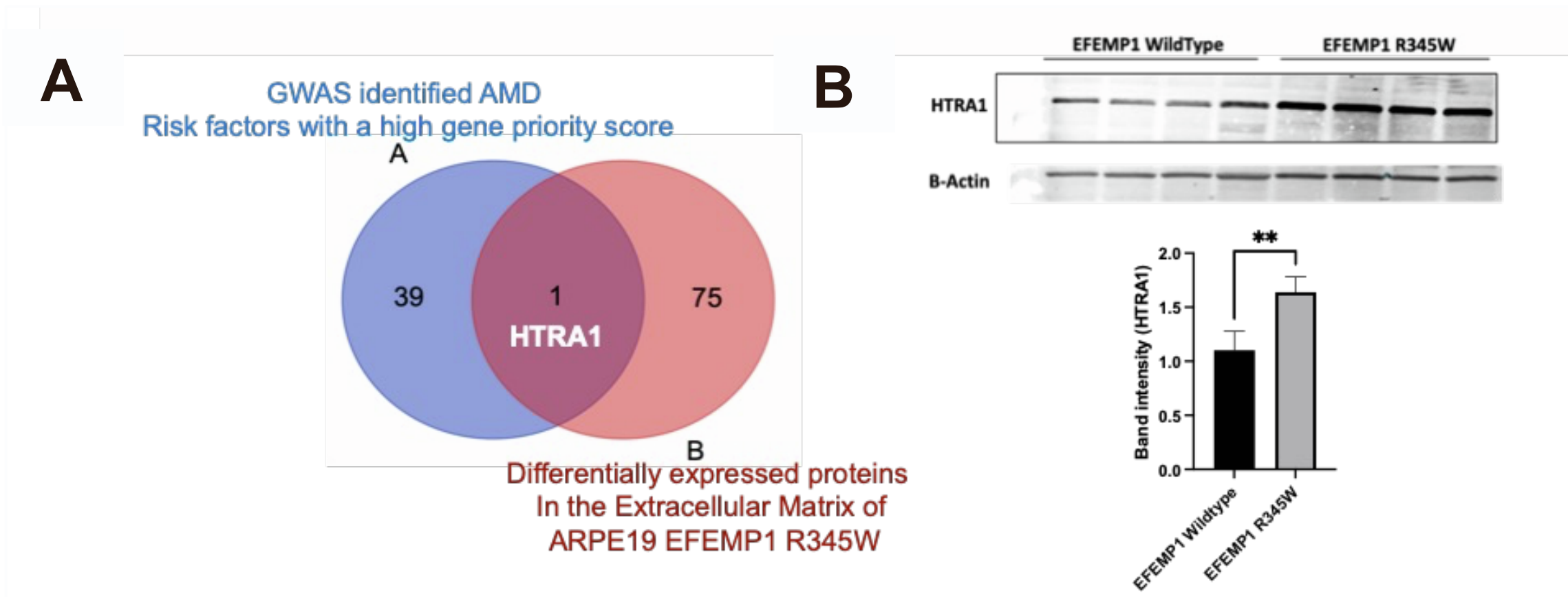

Figure S2: HTRA1 is upregulated in the extracellular matrix of ARPE19 R345W cells. A) Venn diagrham of GWAS identified risk factors with a high gene prioirty score compared with top changing proteins in the extracellular matrix of the ARPE19 R345W cells; HTRA1 is the only common target shared. **C)** HTRA1 protein levels are changing significantly in ARPE19 R345W cells (n=4), Students T-test, p<0.01 **.

A

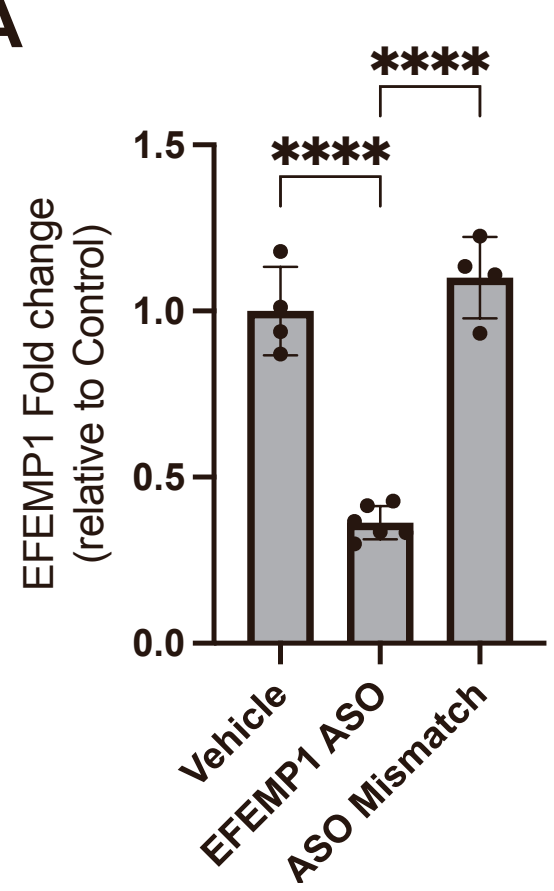

Figure S3: A) Antisense oligonucleotide with two mismatched nucleotides shows no effect on EFEMP1 expression in iPSC-RPE cells (n=4-6), one-way ANOVA, $p<0.0001$ ****.

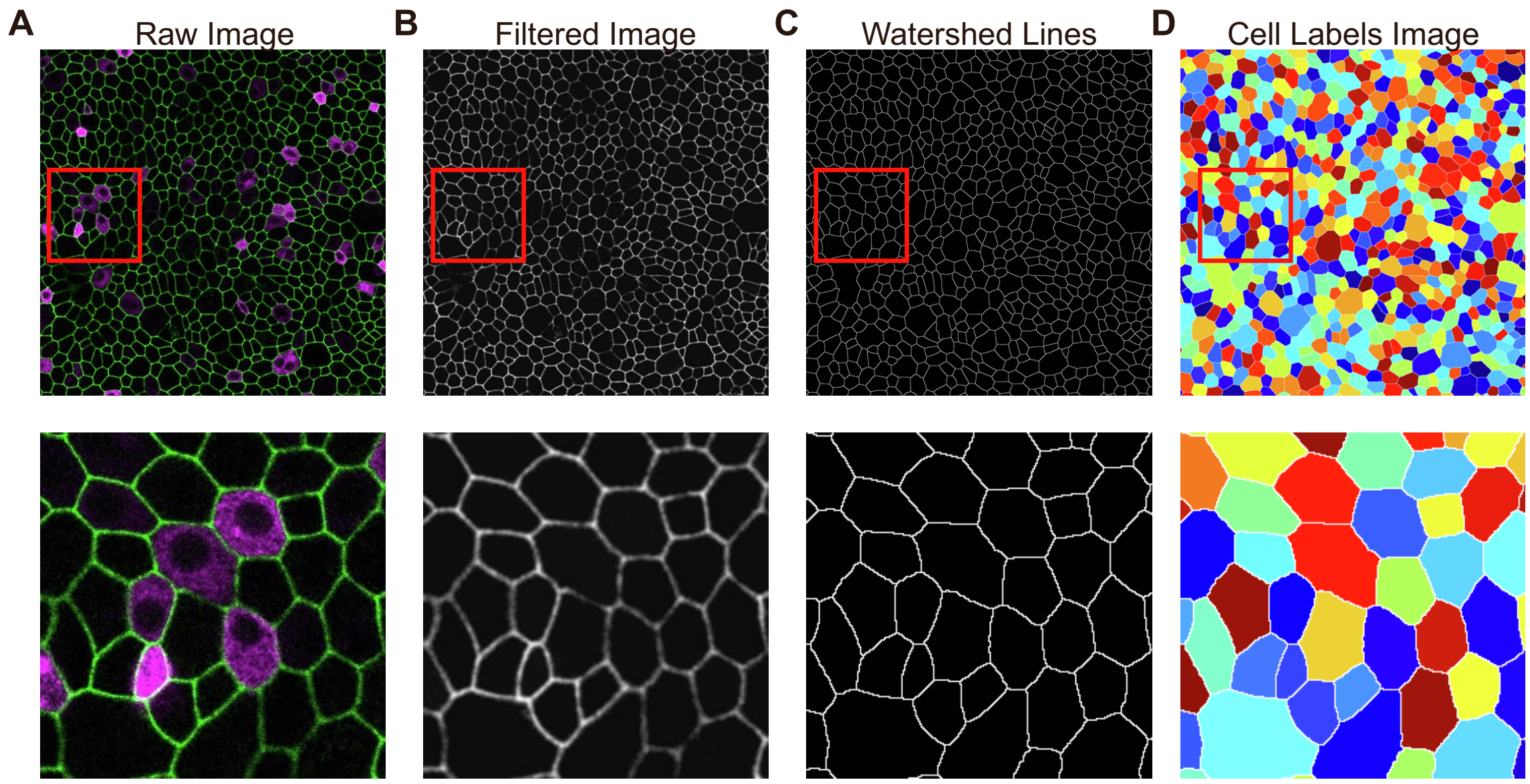

Figure S4: Image segmentation methodology. A) Raw 2-channel image of both Zo-1 (green) and EFEMP1 (magenta). **B)** The raw Zo-1 image was Gaussian filtered and local minima were suppressed using an h-minima transform. **C)** The watershed transform was applied to the filtered image to find the boundary lines of the cells (binary image). **D)** The watershed result was used to uniquely label each cell, shown here through assigning each cell a unique color. **A'-D')** Zoomed-in images of **(A-D)** for the ROI outlined by the red boxes.

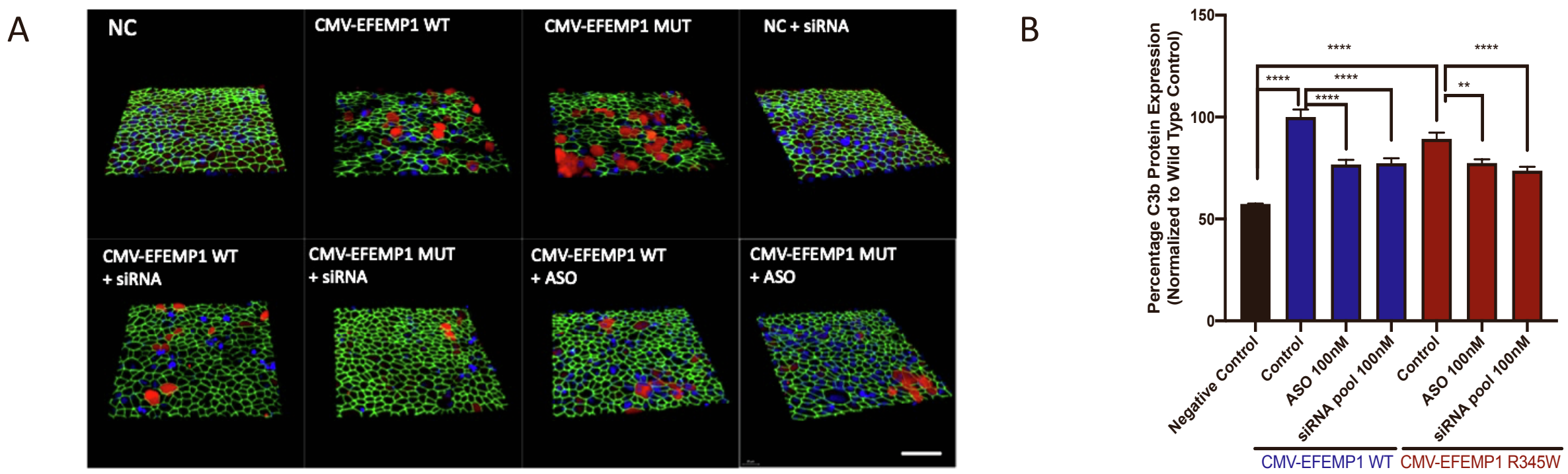

Figure S5: Representative 3D confocal images in iPSC-RPE showing after Lentiviral overexpression of either wild type EFEMP1 and EFEMP1R345W (N=4). A) C3/C3b increased significantly after overexpression of EFEMP1 (x63). iPSC-RPE cells overexpressing EFEMP1 were treated with an ASO Gapmer 100nM or a pool of EFEMP1 siRNAs 100nM to measure knockdown of EFEMP1 and the effect on C3/C3b. C3/C3b: marked in red Scale bar 40μm B) Data was analyzed using a one-way ANOVA. (**) $p < 0.01$, (****) $p < 0.0001$

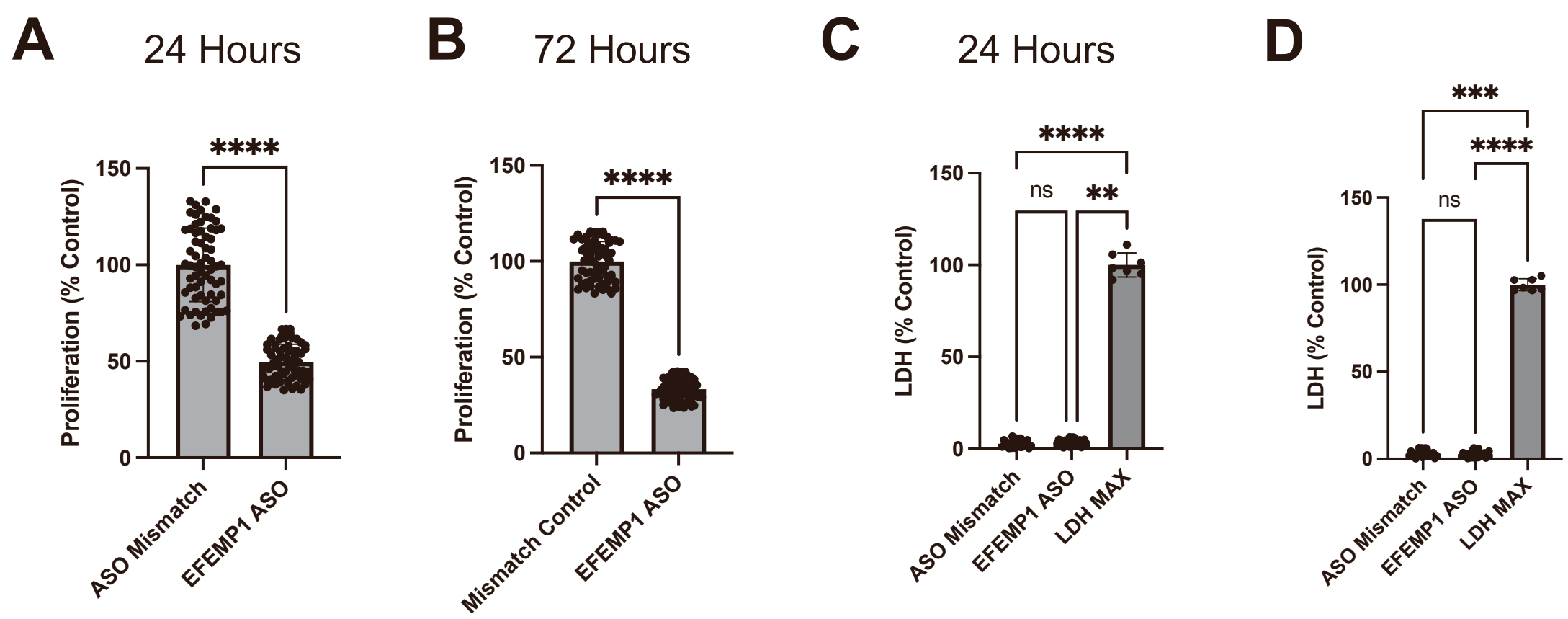

Figure S6: Proliferation in HMRECs at **A)** 24 and **B)** 72 hours after EFEMP1 ASO transfection measured by cyQUANT proliferation assay (n=24), Mann-Whitney test, p<0.0001 ****. LDH assay shows no cytoxicty of EFEMP1 ASO at **C)** 24 hours and **D)** 72 hours. (n=8-24), Kushal Wallis test, p<0.01 **, p<0.001***, p<0.0001 ****.

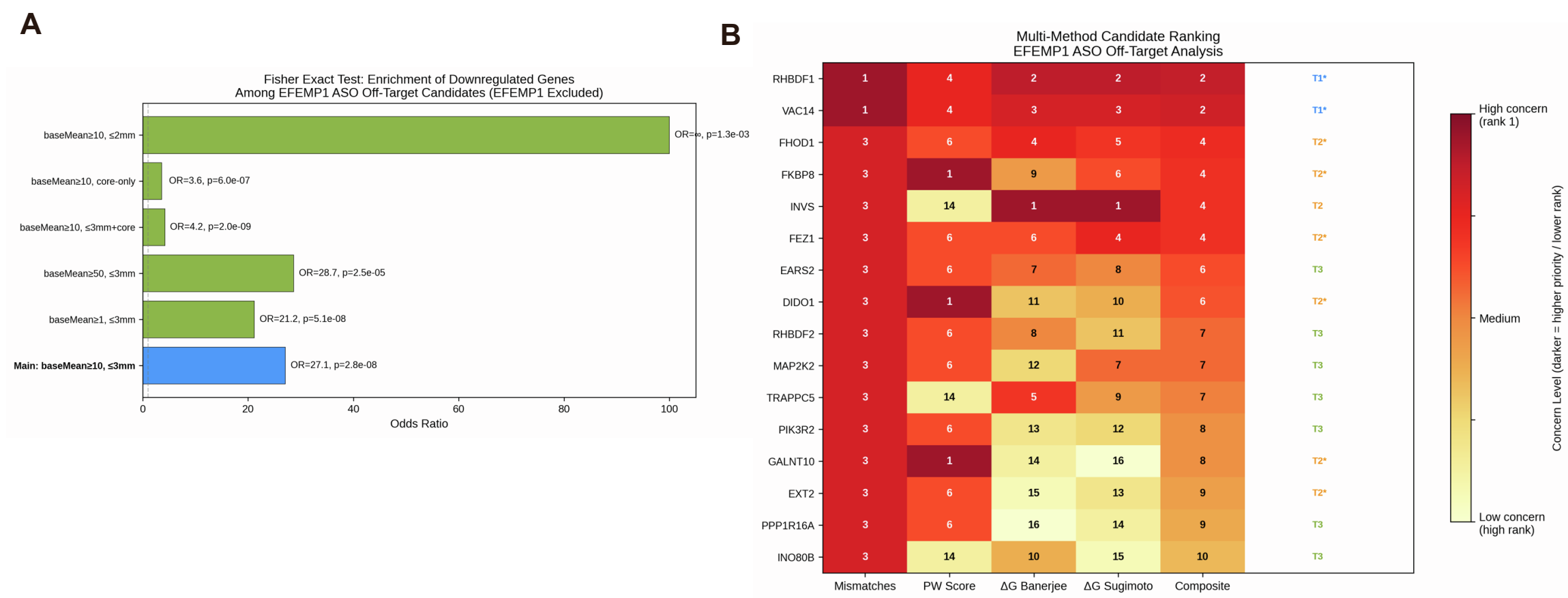

Figure S7: In silico assessment of potential EFEMP1 ASO off-target effects.

A) Fisher exact test enrichment analysis evaluating whether predicted EFEMP1 ASO off-target candidate sets were overrepresented among genes significantly downregulated after EFEMP1 ASO treatment compared with ASO mismatch control. Candidate sets were defined by full 20-mer sequence similarity, central gapmer-core matching, and combined criteria. EFEMP1 was excluded from all off-target enrichment analyses because it represents the intended on-target transcript. The primary expressed-gene background was defined as genes with DESeq2 baseMean ≥10 and non-missing differential-expression statistics. **B**) Multi-method prioritization of candidate off-target genes based on full-length mismatch count, gap/wing position-weighted mismatch score, predicted DNA:RNA duplex free energy, and RNA-seq differential expression. RHBDF1 and VAC14 were the highest-priority candidates because they had only two mismatches to the EFEMP1 ASO target sequence, relatively stable predicted DNA:RNA duplexes, and significant downregulation. These computational predictions identify candidate sequence-dependent off-targets but do not prove direct RNase-H-mediated cleavage.

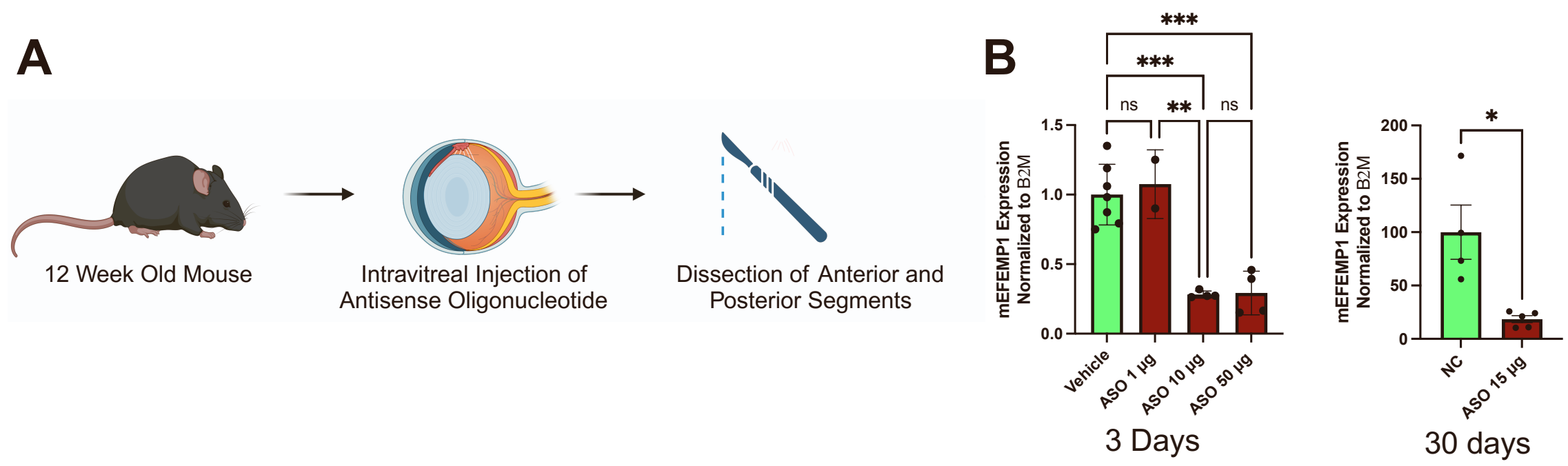

Figure S8: A) Schematic of ASO intravitreal injection in C57BL6/J mice with EFEMP1 targeted ASO and dissection of the eye into anterior and posterior segments for molecular analysis. **B)** qPCR showing EFEMP1 mRNA expression in the mouse posterior segment at 3 days after IVT injection of EFEMP1 targeted ASOs at 1, 10 and 50µg or after 30 days at 15µg. (n=2-6) one-way ANOVA or Students T-test, p<0.05 *, p<0.01 **, p<0.001 ***.

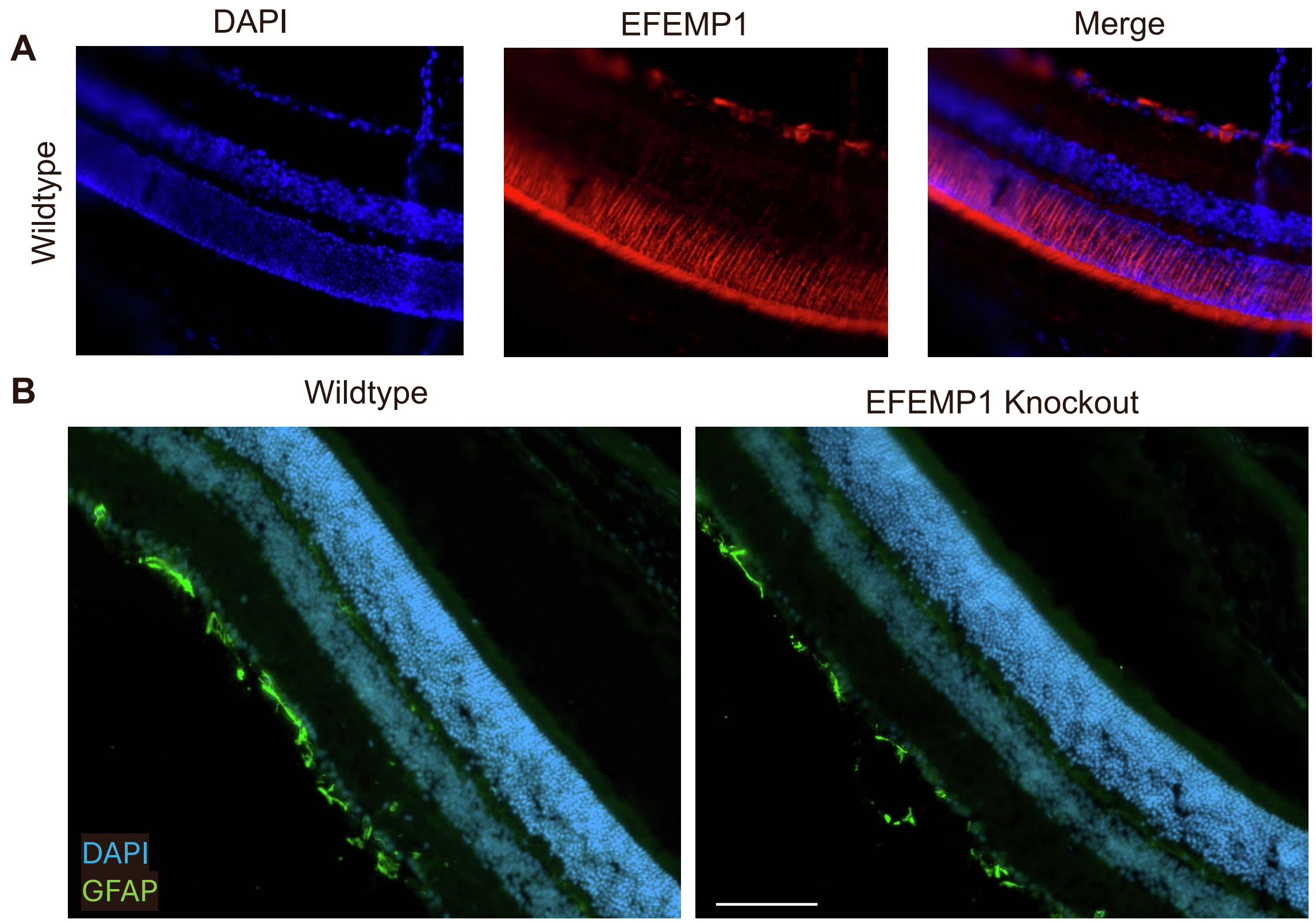

Figure S9: A) Representative images showing EFEMP1 expression in muller cells of retina in C57BL6J mice using Leica Sp8 confocal imaging**. B)** Representative images of Glial Fibrillary Acidic Protein (GFAP; green) and DAPI (blue) staining in the retina of wild type and EFEMP1 knockout mice. (Scale 100μM)

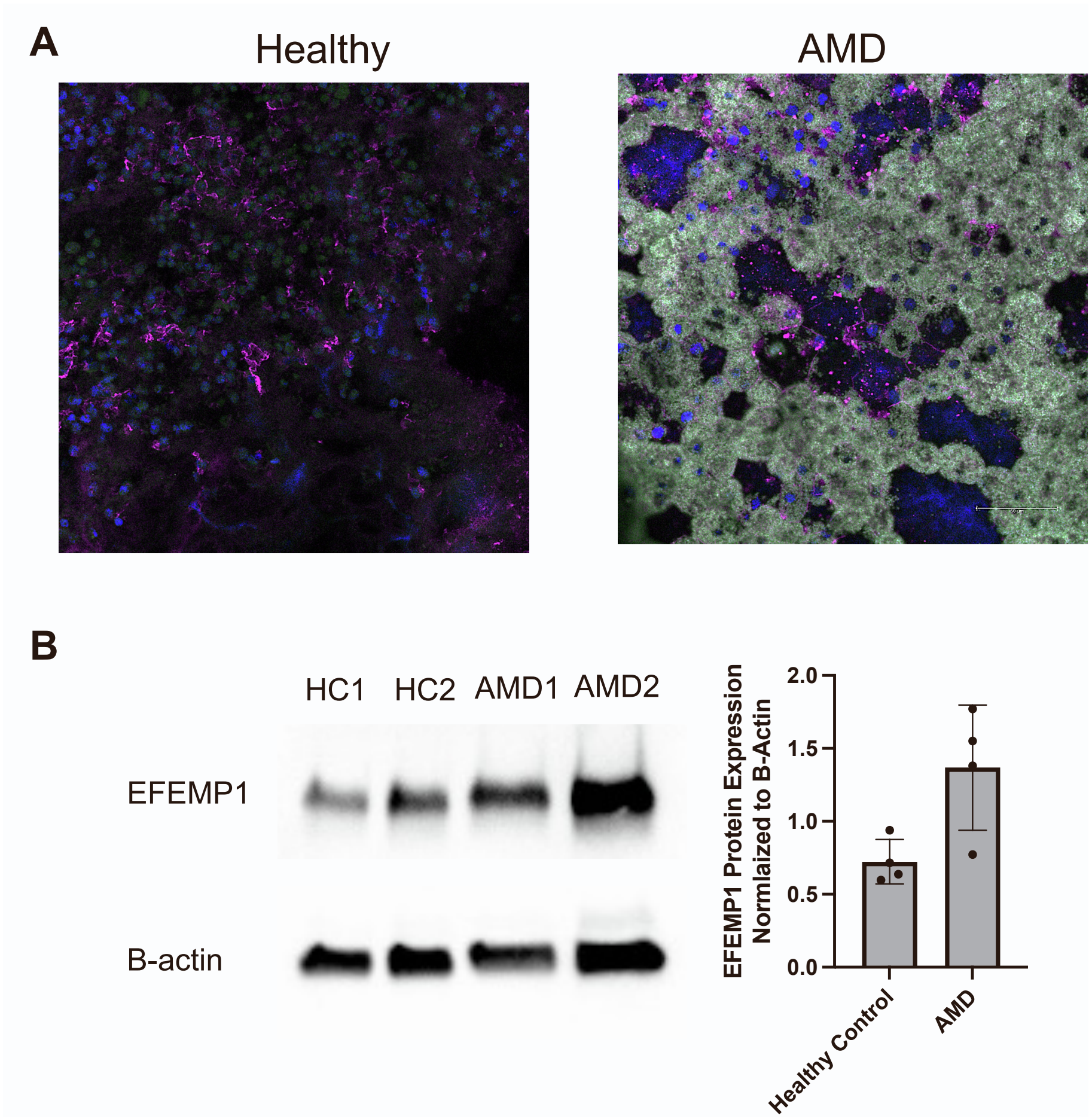

Figure S10: EFEMP1 is overexpressed in AMD tissue. A) Immunofluroscence staining of healthy donor RPE/choroids and AMD RPE/choroids with antibodys against EFEMP1 (Green), TIMP3 (magenta) and DAPI (blue). Scale Bar: 50μm **B)** Western blotting of healthy and AMD RPE/choroid lystaes showing increased EFEMP1 levels in AMD eyes (n=4).

Table S1: ARPE19 vs ARPE19 R345W GO terms**.**

Table S2: HMREC GO terms.

Table S3. Post-mortem RPE/Choroid Tissue Donor Demographics

Patient #	Age	Gender	Cause of Death	Past Ocular History
**1**	53	M	Anoxic Brain Injury	Healthy
**2**	66	F	COPD	Healthy
**3**	58	M	Cardiac arrest	Healthy
**4**	68	F	End stage COPD	Healthy
**5**	80	F	Heart Failure	AMD
**6**	85	M	Metastatic small cell ancer	AMD
**7**	80	F	Heart Failure	AMD

Table S4. Summary of forward and reverse primer sequences.

Target	Species	Forward	Reverse
Efemp1	Mouse	5' CAT GAG CAT CCG ATC TGA CA 3'	5' GGC TTC TTT GTC GTA GGT AGA 3'
B2m (House keeping)	Mouse	5' TGG TCT TTC TGG TGC TTG TC 3'	5' GGG TGG AAC TGT GTT ACG TAG 3'
EFEMP1	Human	5' CAA CCC TTC CCA CCG TAT C 3'	5' CAC ACT TGG TCT GCT CTA CAG 3'
C3	Human	5' GCC TTT GTT CTC ATC TCG CT 3'	5' AGT CTC CTG CTT TAG TGA TGC 3'
B2m (House keeping)	Human	5' GGA CTG GTC TTT CTA TCT CTT GT 3'	5' ACC TCC ATG ATG CTG CTT AC 3'

Table S5. Summary of primary and secondary antibodies sources and dilutions.

Target	Primary Antibody	Company (Cat No.)	Dilution (application)
EFEMP1	Rabbit mAb	Abcam (ab246547)	1:250 (ICC), 1:1000 (WB)
EFEMP1	Mouse mAb	Santa Cruz (sc-33722)	1:200 (IF)
β-actin	Mouse mAb	Cell Signalling (3700S)	1:1000 (WB)
β-actin	Rabbit mAb	Cell Signalling (4970S)	1:1000 (WB)
HIF1α	Rabbit mAb	Cell Signalling (36169S)	1:1000 (WB)
TIMP3	Rabbit pAb	ThermoFisher Scientific (710404)	1:200 (IF)
**Secondary Antibody**		**Company (Cat No.)**	**Dilution (application)**
IRDye 800CW anti-mouse		Licor (926-32212)	1:2000 (WB)
IRDye 680R anti-rabbit		Licor (926-88071)	1:2000 (WB)
Anti-rabbit IgG HRP-conjugated		R&D Systems (HAF008)	1:2000 (WB)
Donkey anti-rabbit AlexaFluor 647		ThermoFisher Scientfic (A-31573)	1:1000 (ICC/IF)
Donkey anti-mouse AlexaFluor 594		ThermoFisher Scientfic (A-21203)	1:1000 (ICC/IF)

Supplemental Materials and Methods:

Extracellular matrix processing: We compared the extracellular matrix composition of ARPE19 and ARPE19 R345W after 4 weeks of differentiation in transwells. The transwells were decellularized by incubating them with sterile 0.5% Triton X-100 (Sigma; T8787-50ML) + 20 mM NH_4OH in PBS (Thermofischer; AM9624) 1.5 ml was added to the bottom chamber and 0.5 ml to the apical chamber) for 5 min at 37 °C, followed by three washes with PBS. The extracellular matrix was collected in M-PER (Thermofischer; 78503) buffer and then prepared in native gel buffer and ran on a poly-acrylamide gel followed by silver staining (Thermofischer; 24612).

Mass spectrometry: Proteins from the extracellular matrix were extracted using M-PER Mammalian Protein Extraction Reagent (Thermo Fisher Scientific; 78501) according to the manufacturer's instructions. Samples in solution were subsequently processed and analyzed as a fee-for-service at the Taplin Biological Mass Spectrometry Facility (Harvard Medical School, Boston, MA, USA). Prior to mass spectrometric analysis, detergent was removed from protein samples using detergent removal spin columns. Peptide and protein identification was then performed by searching acquired spectra against a reference database of known peptide sequences using Sequest (Thermo Fisher Scientific), with standard parameters for precursor and fragment ion mass tolerance, and a false discovery rate (FDR) threshold of ≤1% applied at the peptide and protein levels.

In silico EFEMP1 ASO off-target analysis: Potential sequence-dependent off-target effects of the EFEMP1 ASO were evaluated using an in silico transcriptome-complementarity analysis. The EFEMP1 ASO sequence, 5′-CTGTCAGCATCTCCAGGTCC-3′, was analyzed against GENCODE v46 transcript sequences. Because the ASO binds RNA in an antiparallel orientation, the corresponding sense transcript target motif, 5′-GGACCTGGAGATGCTGACAG-3′, was used as the query sequence for transcriptome scanning. Transcript sequences were represented in DNA alphabet for computational analysis. For each

transcript, a 20-nucleotide sliding window was advanced one nucleotide at a time across the sense transcript sequence, and the number of mismatches between each window and the query motif was counted. Each transcript was assigned the minimum mismatch count observed across all windows. Gene-level candidate status was then assigned using the best, lowest mismatch count observed across any transcript associated with that gene. EFEMP1 was excluded from all off-target enrichment analyses because it represents the intended on-target transcript.

Three candidate sets were defined. Set A included genes with full 20-mer complementarity allowing ≤2 mismatches. Set B included genes with full 20-mer complementarity allowing ≤3 mismatches. Set C included genes with an exact match to the central 10-mer gapmer core, independent of flanking sequence context. Set A and Set B were considered the primary sequence-similarity candidate sets because they preserve near-complementarity across the full ASO target sequence. Set C was included as a lower-confidence sensitivity set because exact matching to a 10-mer sequence can occur more frequently by chance, especially when the flanking wing regions contain multiple mismatches. However, Set C was retained because the central DNA gap is the region expected to support RNase-H-mediated cleavage in a gapmer ASO.

To account for the 5-10-5 gapmer architecture of the EFEMP1 ASO, a position-weighted mismatch score was calculated for each candidate. The ASO was modeled as containing a 5-nucleotide 5′ wing, a 10-nucleotide central DNA gap, and a 5-nucleotide 3′ wing. The central DNA gap, corresponding to positions 6–15 using 1-indexed coordinates, is expected to support RNase-H-mediated cleavage of the target RNA, whereas the flanking wing regions primarily contribute to duplex stability and nuclease resistance. The position-weighted score was calculated as:

PW score = (gap mismatches × 3.0) + (wing mismatches × 1.0)

where gap positions were defined as positions 6–15 and wing positions as positions 1–5 and 16–20. The 3:1 weighting was used as a heuristic prioritization scheme to reflect the greater expected functional

importance of gap-region complementarity for RNase-H-mediated cleavage. Lower position-weighted scores were interpreted as higher-priority predicted off-target candidates.

DNA:RNA duplex free energy was estimated to rank predicted off-target candidates by the expected stability of ASO:RNA hybrid formation. This analysis was used as a secondary prioritization approach and not as definitive evidence of direct off-target cleavage. Two published nearest-neighbor parameter sets were used. Banerjee et al. 2020 was used as the primary DNA:RNA thermodynamic model because it was measured under lower-salt conditions closer to physiological ionic strength. Sugimoto et al. 1995 was used as a sensitivity analysis because it is a classic DNA:RNA hybrid thermodynamic reference set. Mismatch-associated free-energy penalties were incorporated from Watkins et al. 2011 where applicable. For mismatch classes or sequence contexts not represented in the available parameter set, approximate mismatch penalties were applied and interpreted cautiously.

The ASO was modeled as a DNA strand hybridized antiparallel to the RNA transcript sequence. Watson-Crick RNA:DNA base pairs were defined as A:T, U:A, G:C, and C:G. For each candidate site, predicted DNA:RNA duplex ΔG was calculated at 37°C. Relative ΔG was calculated as:

$\Delta\Delta G = \Delta G_{\text{off-target}} - \Delta G_{\text{on-target}}$

More negative ΔG values indicate more stable predicted DNA:RNA duplexes, whereas smaller ΔΔG values indicate off-target candidates closer in predicted binding stability to the intended EFEMP1 target site. Candidate genes were prioritized based on concordance across full-length mismatch count, position-weighted mismatch score, DNA:RNA ΔG, and observed RNA-seq downregulation.

To test whether predicted off-target candidates were enriched among downregulated genes, Fisher exact tests were performed using expressed genes as the background. The primary expressed-gene background was defined as genes with DESeq2 baseMean ≥ 10 and non-missing differential-expression statistics. Genes were classified as significantly downregulated if adjusted P value <0.05 and log2 fold change ≤ -0.58 in EFEMP1 ASO-treated HMRECs compared with ASO mismatch-treated controls.

Sensitivity analyses were performed using alternative expressed-gene thresholds and candidate definitions, including full 20-mer $\leqslant$2 mismatches, full 20-mer $\leqslant$3 mismatches, core-only exact matches, and combined candidate sets.
